# Supplementary material for: Empowering alcohols as carbonyl surrogates for Grignard-type reactions
Source: Nat Commun. 2020 Nov 26;11:6022. doi: 10.1038/s41467-020-19857-9 (PMC7691337; doi:10.1038/s41467-020-19857-9)
Supplement: Supplementary file 1 — Supplementary Information [file 41467_2020_19857_MOESM1_ESM.pdf]

Supplementary Information for

Empowering Alcohols as Carbonyl Surrogates for Grignard-Type Reactions

Chen-Chen Li, Haining Wang, Malcolm M. Sim, Zihang Qiu, Zhang-Pei Chen, Rustam Z. Khaliullin and Chao-Jun Li*

Department of Chemistry and FQRNT Centre for Green Chemistry and Catalysis, McGill University, 801 Sherbrooke Street West, Montreal, Quebec H3A 0B8, Canada. * Email: cj.li@mcgill.ca

Table of Content

I. General experimental information 2

II. Experimental procedures 3

i. Preparation of hydrazone or hydrazone solution 3

ii. General procedure for Table 1-2, Scheme 2-4 4

III. Synthesis of active species 7

i. Preparation of Ru-PNP-1 7

ii. Preparation of Ru-PNP-2 7

IV. Mechanistic study 9

i. DFT study for the dehydrogenation step 9

ii. Proposed mechanism 11

V. Spectroscopic data of products 12

VI. NMR spectra of products 25

VII. Supplementary references 99

I. General experimental information

Reaction Setup: All reactions were carried out in flame-dried V-shaped microwave reaction vials which were covered by aluminum seals with PTFE-faced silicone septa, under an atmosphere of nitrogen unless otherwise stated. All reaction temperatures corresponded to oil bath temperatures. All air and moisture-sensitive catalysts, ligands, and reagents were stored and charged in MBRAUN UNIlab Pro Glove Box Workstation unless otherwise stated.

Purifications: All work-up and purification procedures were carried out with reagent-grade solvents. Analytical thin-layer chromatography (TLC) was performed using E. Merck silica gel 60 F254 pre-coated plates (0.25 mm). Flash column chromatography was performed with E. Merck silica gel P60 (40–63 µm particle size, 230–400 mesh) (SiO_2). Unless otherwise specified, "SiO_2" refers to P60 grade silica gel. Visualization was accomplished with UV light and/or iodine (I_2) or Vanillin solution. Retention factor (R_f) values reported were measured using a 10 × 2 cm TLC plate in a developing chamber containing the solvent system (10 mL) described. Automated flash column chromatography was performed on Biotage Isolera™ Spektra Systems with ACI™.

Solvents: 2-methyl-tetrahydrofuran (2-Me-THF), ordered from Sigma Aldrich without any purification. Solvents for filtration, transfers and chromatography, were dichloromethane (CH_2Cl_2) (ACS grade, amylene stabilized), acetone (ACS grade), ethyl acetate (EtOAc) (Fisher, ACS grade), hexane (Fisher, ACS grade), pentane (ACS grade), methanol (ACS grade).

Chemicals: In the model study, benzaldehyde (Aldrich) were distilled prior to use. Other chemicals that are commercially available and used without further purification: 2-penten-1-ol (Aldrich), $Ru(PPh_3)_4Cl_2$ (Aldrich), $Ru(PPh_3)_3Cl_2$ (Aldrich), dcypf (Aspira), potassium phosphate (Aldrich), hydrazine hydrate (Reagent Grade, 64–65% wt, Aldrich), mesitylene (Aldrich), 1,3,5-trimethoxylbenzene (Aldrich), anhydrous sodium sulfate. All liquid carbonyls were distilled, and solid ones were recrystallized prior to use. The **PNP** pincer ligands (**L1**, **L2**, **L3**) were purchased from Aldrich. All the alcohol substrates are commercially available (Aldrich, Oakwood & Combi Block)

NMR Spectroscopy: Nuclear magnetic resonance (^{1}H and ^{13}C NMR) spectra were recorded on a Bruker AV500 equipped with a 60-position Sample Xpress sample changer (^{1}H, 500 MHz; ^{13}C, 125 MHz), a Varian MERCURY plus-500 spectrometer (^{1}H, 500 MHz; ^{13}C, 125 MHz) or Bruker AV400 spectrometer (^{1}H, 400 MHz; ^{13}C, 100 MHz). Chemical shifts for both ^{1}H NMR and ^{13}C NMR spectra are expressed in parts per million (ppm) units downfield from TMS, with the solvent residue peak as the chemical shift standard ($CDCl_3$: δ 7.28 ppm in ^{1}H NMR; δ 77.00 ppm in ^{13}C NMR). Data are reported as following: chemical shift, multiplicity (s = singlet, d = doublet, dd = doublet of doublets, t = triplet, td = triplet of doublets, q = quartet, quin = quintet, sep = septet, m = multiplet), coupling constants *J* (Hz), and integration.

Mass Spectrometry: Mass spectrometry (MS) was performed by the McGill Chemistry Department Mass Spectrometry Facility. High resolution mass spectra were recorded using electrospray ionization (ESI+) and/or atmospheric pressure chemical ionization APCI (+/-), performed either on "Exactive Plus Orbitrap" a ThermoScientific high resolution accurate mass (HR/AM) FT mass spectrometer, or a Bruker Daltonics Maxis Impact quadrupole-time of flight (QTOF) mass spectrometer.

Characterization of Products: For the products, most of which are known compounds, we report the virtual states, NMR spectra and HRMS data.

DFT calculation: All the calculations were carried out at the B3LYP/6-31G(d,p) level (LANL2DZ for Ru), using the Gaussian 16, Rev A.03 suite of programs.[1] Harmonic frequencies were calculated at the same level to characterize the stationary points and to determine the zeropoint energies (ZPE). Intrinsic reaction coordinate (IRC) studies were performed in ambiguous cases to confirm the relation of the transition states with the corresponding minima.

II. Experimental procedures

i. Preparation of hydrazone or hydrazone solution

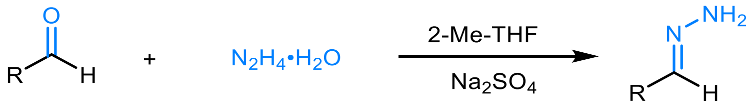

0 **1**

Procedure A: For Table 1: 2-Me-THF (4 mL) was added first into a small bottle with a stir bar. Then, hydrazine monohydrate (0.35 mL, 7 mmol) was added into the bottle. After that, **0** (5 mmol)* was added dropwise into the stirred solution and the mixture was stirred for 5 min. Next, proper amount of anhydrous Na_2SO_4 was added to remove water. After stirring for another 3h, the so-formed solution was ready to use.

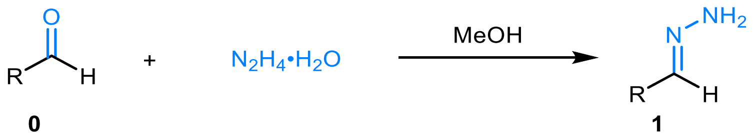

Procedure B: For Table 2, Figure 2, Figure 3: MeOH (5 mL) was added first into a small bottle with a stir bar. Then, hydrazine monohydrate (0.75 mL, 15 mmol) was added into the bottle. After that, **0** (10.0 mmol) was added dropwise into the stirred solution and the mixture was stirred for 5 min. After stirring for 3 h, ***a.*** if the solution is a homogeneity, the so-formed solution was concentrated by vacuum to dryness. Next, the crude hydrazone was frozen-dried under vacuum for three times to remove excess amount of hydrazine hydrate. The so formed hydrazone was directly used without further purification. ***b.*** if precipitates formed from the solution, the solid was filtered and washed with small potion of MeOH and then dried under vacuum, after which the hydrazone can be used directly without further purifications.

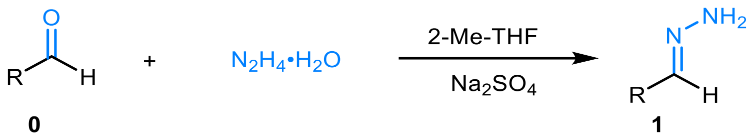

Procedure C: For Figure 2, 3ks, 3ls: 2-Me-THF (5 mL) was added first into a small bottle with a stir bar. Then, hydrazine monohydrate (0.4 mL, 8 mmol) was added into the bottle. After that, **0** (6 mmol) was added dropwise into the stirred solution and the mixture was stirred for 5 min. Next, proper amount of anhydrous Na_2SO_4 was added to remove water. After stirring for another 3h, the so-formed solution was ready to use.

ii. General procedure for Table 1-2, Scheme 2-4

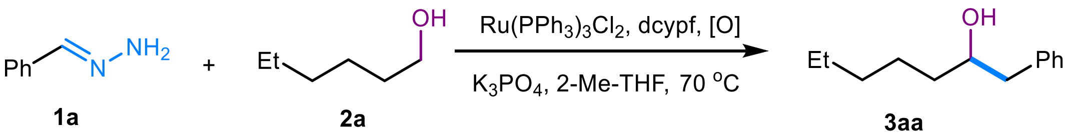

General procedure for Table 1: $Ru(PPh_3)_2Cl_2$ (0.01 mmol), dcypf (0.01 mmol), K_3PO_4 (0.22 mmol), oxidant (0.4 mmol, 2 equiv)* were added into a V-shaped reaction tube with a stir bar in the glovebox. Then, the reaction tube was sealed and moved out of the glovebox. After that, **1a** solution (prepared through ***Procedure A*** , 0.22 mL, 0.25 mmol) was added first and followed by the addition of **2a** (25.0 µL, 0.2 mmol). The mixture was stirred for 24h. Then, 1,3,5-trimethoxylbenzene (11.2 mg, 0.067 mmol) was added in the mixture as standard. Then, the solution was filtered by celite and concentrated to dryness. The crude mixture was diluted by $CDCl_3$ to run the ^1H NMR test to determine the ^1H NMR yield.

*For entry 2, the reaction tube was sealed before exposed to air for 5 min. For entry 3, after removing reaction tube out of the glovebox, it was charged with O_2 via 3 times vacuum-refill by oxygen balloon.

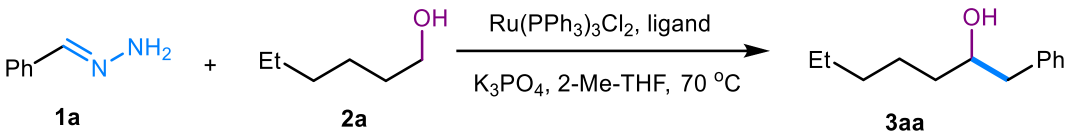

General procedure for Table 2: $Ru(PPh_3)_2Cl_2$ (0.01 mmol), ligand (0.01 mmol), K_3PO_4 (0.4 mmol) were added into a V-shaped reaction tube with a stir bar in the glovebox. Then, the reaction tube was sealed and moved out of the glovebox. After that, 0.5 mL 2-Me-THF was added and followed by the addition of corresponding amount of **1a** (prepared through ***Procedure B***) and **2a** (25.0 µL, 0.2 mmol). The mixture was stirred for 24h under N_2 at 70 °C. After completion, the solution was filtered by celite and concentrated to dryness. Then, 1,3,5-trimethoxylbenzene (11.2 mg, 0.067 mmol) was added in the mixture as standard. The crude mixture was diluted by $CDCl_3$ to run the ^1H NMR test to determine the ^1H NMR yield.

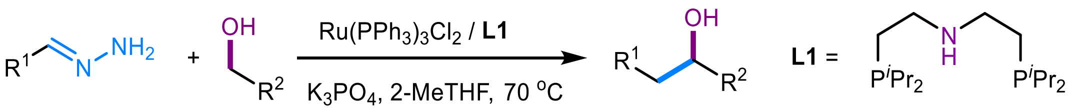

General procedure for Figure 3, Figure 4: $Ru(PPh_3)_3Cl_2$ (0.01 mmol), **L1** (0.01 mmol) and K_3PO_4 (0.4 mmol) and solid substrates were added into a V-shaped reaction tube with a stir bar in the glovebox. Then, the reaction tube was sealed and moved out of the glovebox. After that, 0.5 mL 2-Me-THF was added first followed by the addition of liquid substrates. The mixture was stirred under 70 °C for 24h. The reaction mixture was filtered through a celite plug with 2-3 mL CH_2Cl_2. The solvent was removed by a

rotary evaporator and the residue was purified by column chromatography on silica gel (using hexane and ethyl acetate as eluent) to give the pure product.

General procedure for Figure 3, 3as, 3at, 3au, 3hv and 3hw: **Ru-PNP-3** (0.01 mmol), and K_3PO_4 (0.4 mmol) and solid substrates were added into a V-shaped reaction tube with a stir bar in the glovebox. Then, the reaction tube was sealed and moved out of the glovebox. After that, 0.5 mL 2-Me-THF was added first followed by the addition of liquid substrates. The mixture was stirred under 100 ^oC for 24h. The reaction mixture was filtered through a celite plug with 2-3 mL CH_2Cl_2. The solvent was removed by a rotary evaporator and the residue was purified by column chromatography on silica gel (using hexane and ethyl acetate as eluent) to give the pure product.

General procedure for Figure 3ax-3az: **Ru-PNP-1** (0.01 mmol), K_3PO_4 (0.4 mmol) and solid alcohol (0.2 mmol, if applicable) were added into a V-shaped reaction tube with a stir bar in the glovebox. Then, the reaction tube was sealed and moved out of the glovebox. After that, 0.5 mL 2-Me-THF was added first followed by the addition of **1a** (0.6 mmol, 3 equiv.) and liquid alcohols (0.2 mmol, if applicable). The mixture was stirred under 70 ^oC for 24h. The reaction mixture was filtered through a celite plug with 2-3 mL CH_2Cl_2. The solvent was removed by a rotary evaporator and the residue was purified by column chromatography on silica gel (using hexane and ethyl acetate as eluent) to give the pure product. Specifically, for **3ay,** the mixture was diluted with $CDCl_3$ and run the ^1H NMR test to determine trace amount of desired product.

Procedure for Figure 3 (3ks, 3ls): $Ru(PPh_3)_3Cl_2$ (0.01 mmol), **L3** (0.006 mmol) and K_3PO_4 (0.4 mmol) were added into a V-shaped reaction tube with a stir bar in the glovebox. Then, the reaction tube was sealed and moved out of the glovebox. After that, **1** solution (prepared through ***Procedure B***, 0.55 mL, 0.6 mmol) was added. The mixture was stirred at 100 ^oC for 24h. The reaction mixture was filtered through a celite plug with 2-3 mL CH_2Cl_2. The solvent was removed by a rotary evaporator and the residue was added mesitylene as internal standard. The mixture was diluted with $CDCl_3$ and run the ^1H NMR test to determine trace amount of desired product based on the standard spectrum from literature.[2, 3]

III. Synthesis of active species

i. Preparation of Ru-PNP-1

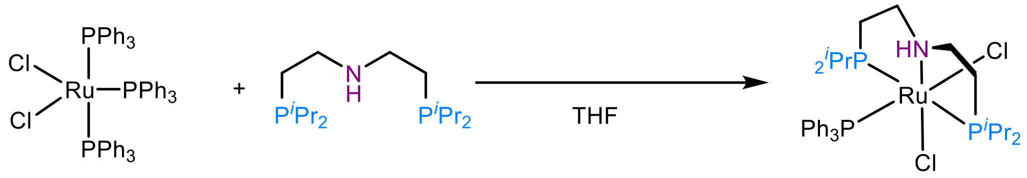

To a solution of $Ru(PPh_3)_3Cl_2$ (958 mg, 1 mmol) in THF (8 mL) was dropwise added bis(2-(diisopropylphosphino)ethyl)amine (3.5 mL, 10 w% in THF, 1 mmol). The mixture was stirred at room temperature for 2h. Then, most of THF was evaporated by rotavapor followed by the addition of pentane (25 mL) while stirring. At this period, solid was precipitated. It was placed at 4 °C for 1h. Then, the solid was collected by filtration which was washed by ether and dried under vacuum to give **Ru-PNP-1** as a light brown solid. The characterization was reported by previous literature.[4]

ii. Preparation of Ru-PNP-2

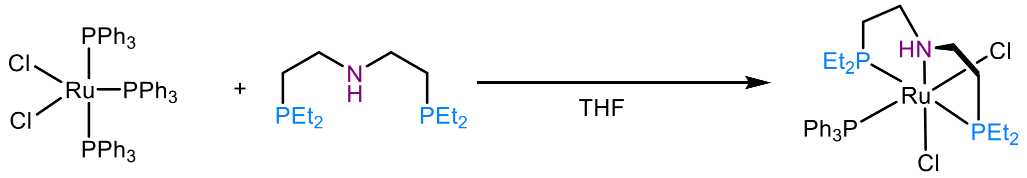

To a solution of $Ru(PPh_3)_3Cl_2$ (958 mg, 1 mmol) in THF (8 mL) was dropwise added bis(2-(diethylphosphino)ethyl)amine (250 mg, 1 mmol). The mixture was stirred at room temperature for 2h. Then, most of THF was evaporated by rotavapor followed by the addition of pentane (25 mL) while stirring. At this period, solid was precipitated. It was placed at 4 °C for 1h. Then, the solid was collected by filtration which was washed by ether and dried under vacuum to give **Ru-PNP-2** as a green to yellow solid.

^{1}H NMR (500 MHz, C_6D_6) δ 8.32 –8.10 (m, 6H), 7.13 – 7.00 (m, 9H), 3.83 – 3.61 (m, 1H), 2.96 – 2.79 (m, 2H), 2.31 – 2.13 (m, 2H), 2.00 – 1.82 (m, 4H), 1.73 – 1.57 (m, 2H), 1.52 – 1.31 (m, 4H), 1.04 – 0.86 (m, 8H), 0.82 (p, J = 7.1 Hz, 6H). **^{13}C NMR** (126 MHz, C_6D_6) δ 143.6 (d, J = 36.5 Hz), 136.1 (d, J = 9.5 Hz), 129.1, 127.7 (d, J = 8.5 Hz), 48.9, 26.3, 14.4 (t, J = 10.8 Hz), 13.4 (t, J = 10.3 Hz), 9.5, 9.0. **^{31}P NMR** (203 MHz, C_6D_6) δ 42.7 (t, J = 28.1 Hz), 33.7 (d, J = 28.0 Hz). **HRMS:** (APCI, *m/z*): calcd. for $C_{30}H_{44}ClNP_3Ru[M-Cl]^+$ 648.1410, found: 648.1413.

^{1}**HNMR** spectrum

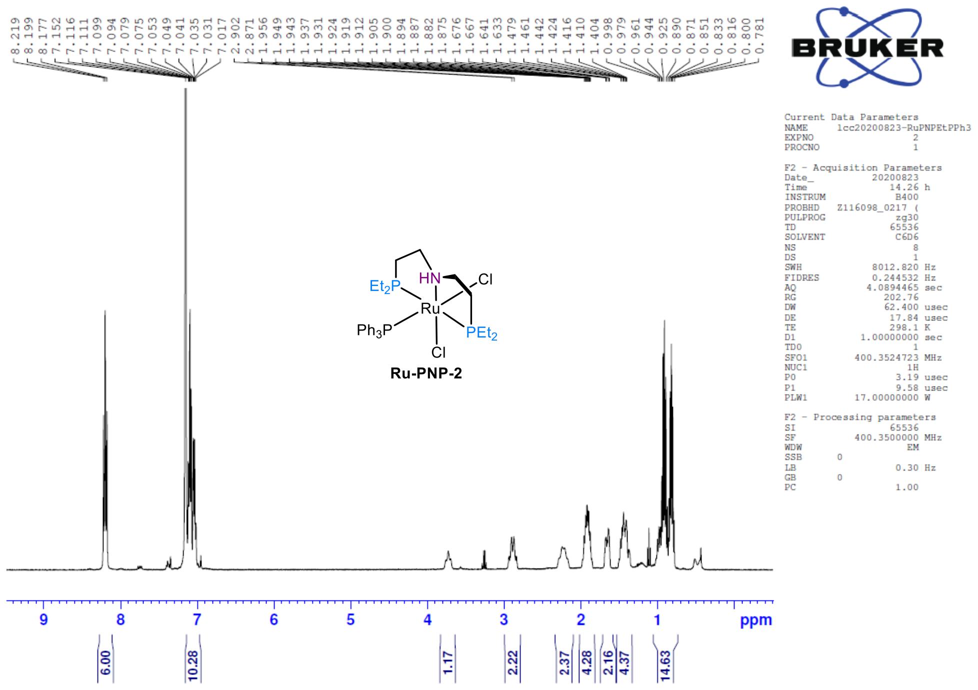

^{13}**C NMR** spectrum

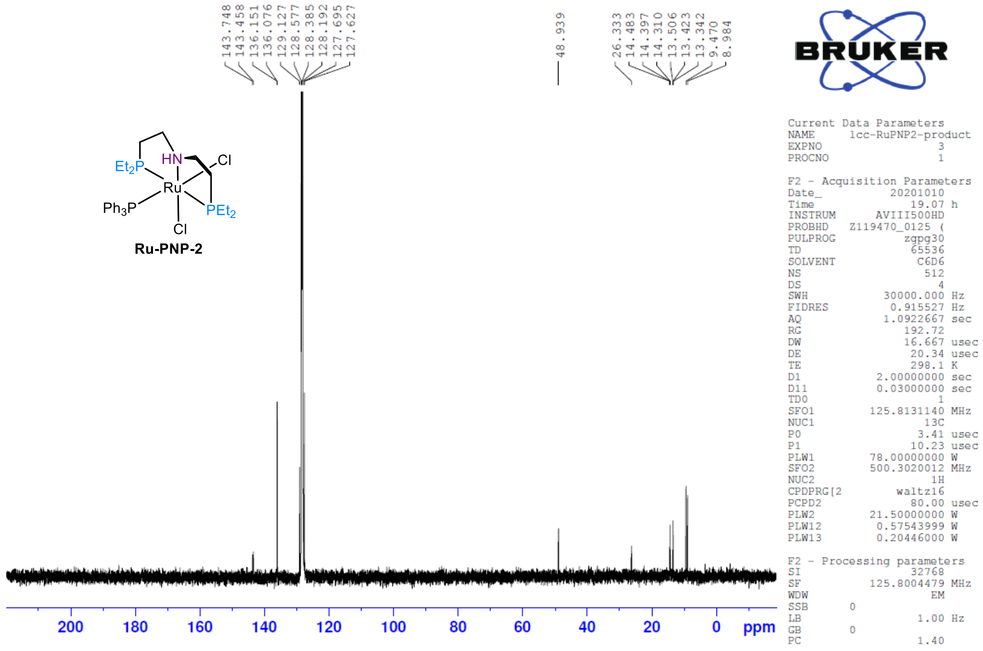

^{31}PNMR spectrum

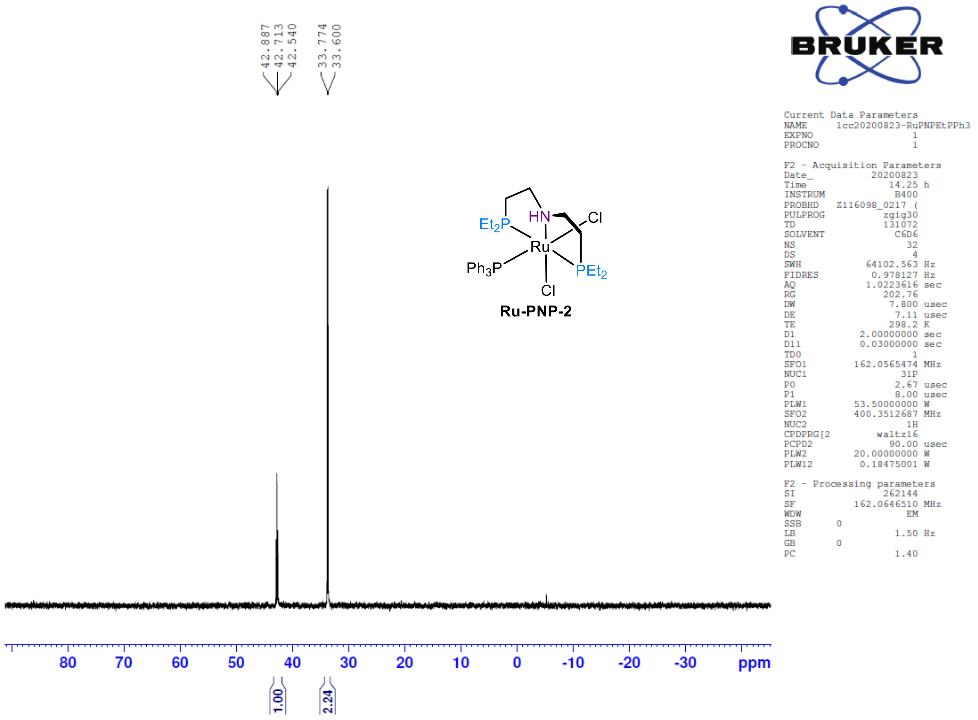

IV. Mechanistic study

i. DFT study for the dehydrogenation step

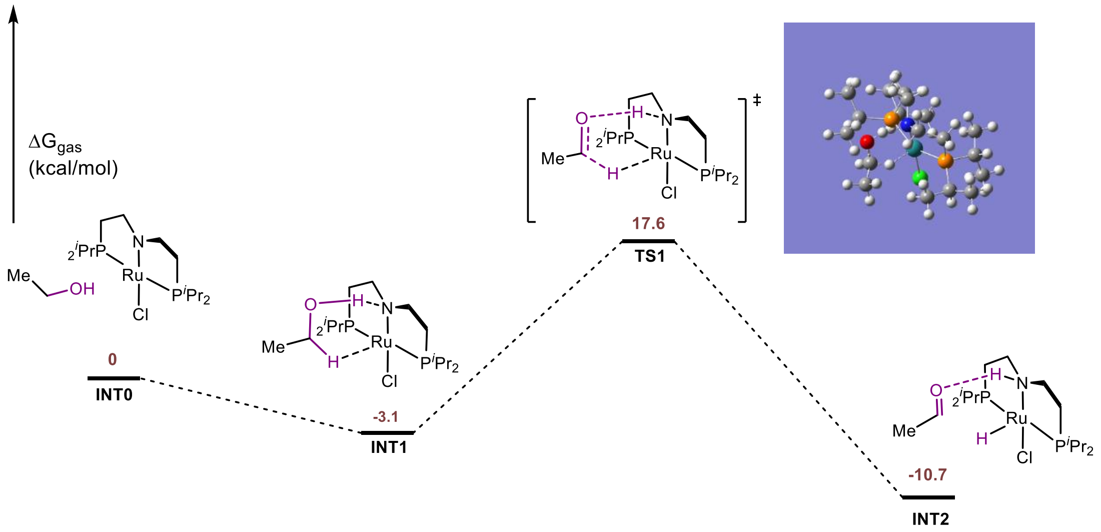

Supplementary Figure 1. Energy diagram of the dehydrogenation step based on DFT calculation. DFT calculation was conducted by Gaussian 16. The IRC study clearly shows the possibility of proposed six-membered ring transition state for dehydrogenation step.

Since the first step (dehydrogenation) is the key step of this dehydrogenative Grignard reaction, we mainly did the calculation for the first step. The result shows that the six-membered-ring transition state we proposed is reasonable in this case (Supplementary Figure 1). Furthermore, we also predicted a four-membered-ring transition state which is the corresponding to the classic *β*-hydride elimination process and the preliminary calculation shows that the free energy of transition state is 54.3 kcal/mol higher than the six-membered-ring one and the intermediate does not show a perfect match with transition state possibly due to the geometry mismatch (thus we did not show on the diagram), which suggested that the six-membered ring transition state is a more favorable transition state in this case. For the 1,2 addition step, we already did several studies in previous work done by us and others.[2,5]

ii. Proposed mechanism

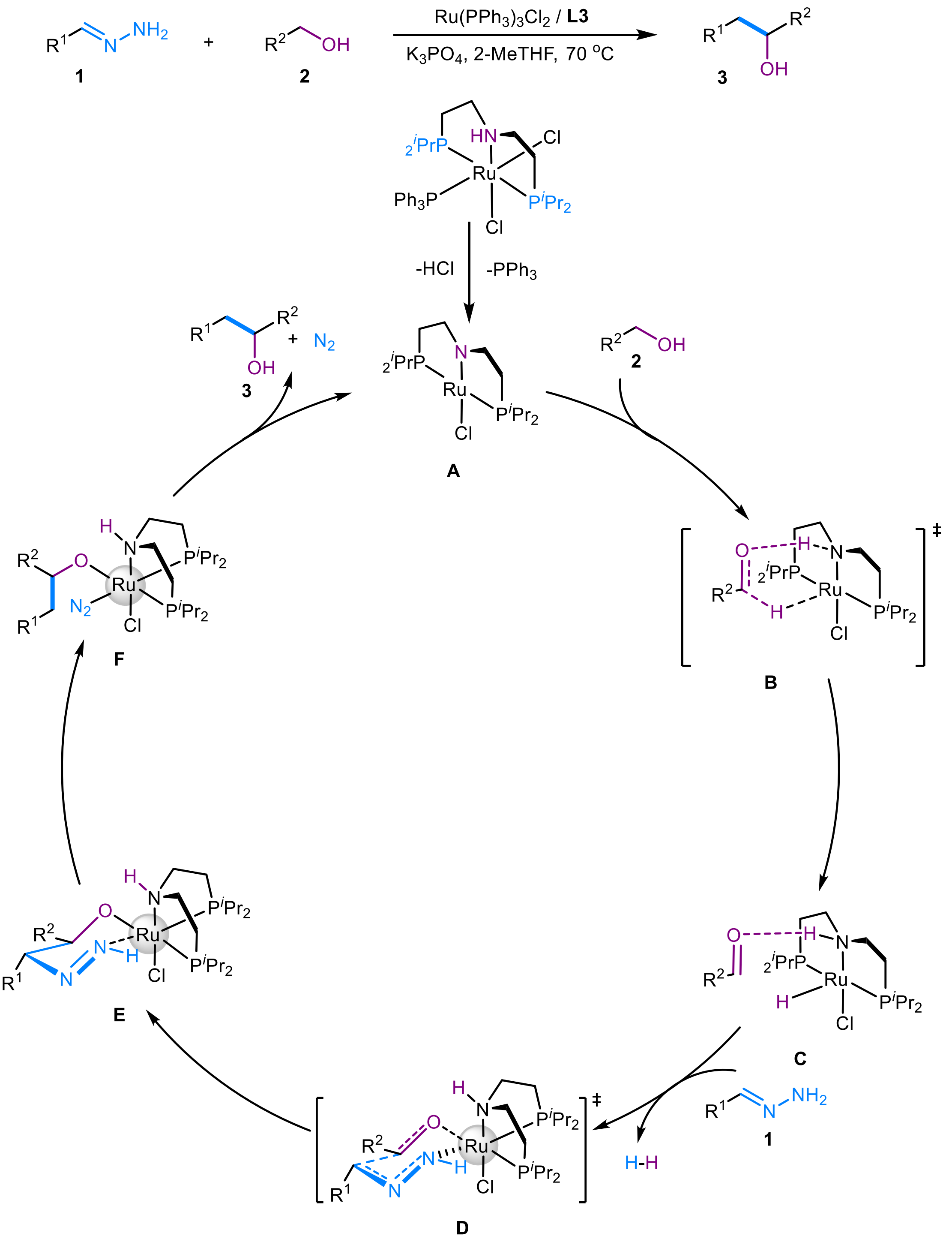

Supplementary Figure 2. Proposed mechanism for alcohol surrogated Grignard reaction. The proposed mechanism starts from the tetracoordinated ruthenium complex. The whole process includes a dehydrogenation step and a C-C bond formation step which are all experience six-membered-ring transition states.

In this proposed mechanism, the intermediate **A** was proposed refer to the previous literature about a similar structure study.[6] In that case, it is shown that the PNP

ruthenium complex would form a square planar structure with the presence of base. The dehydrogenation process **A** to **C**, it is also supported by the DFT calculation and other related literature.[7] For the hydrogen releasing step **C** to **D**, it also has the literature support which suggested that the hydride would protonated to hydrogen gas while nitrogen can serve as an internal base to assist the coordination of hydrazine.[8,9] For the 1,2-addition step, it is already illustrated in our previous work.[2,5]

V. Spectroscopic data of products

Note: references of the characterization data of some known compounds are marked before our characterization data.

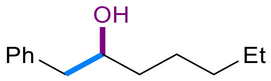

3aa

(Generated following the corresponding general procedure, isolated by column chromatography on silica gel with hexane: EtOAc = 95:5 as eluent, light brown oil, 28 mg, yield: 73%): **^{1}H NMR** (500 MHz, $CDCl_3$) δ 7.38 – 7.31 (m, 2H), 7.31 – 7.16 (m, 3H), 3.90 – 3.79 (m, 1H), 2.86 (dd, *J* = 13.6, 4.2 Hz, 1H), 2.68 (dd, *J* = 13.5, 8.4 Hz, 1H), 1.73 – 1.46 (m, 4H), 1.46 – 1.21 (m, 5H), 0.93 (t, *J* = 6.9 Hz, 3H). **^{13}C NMR** (126 MHz, $CDCl_3$) δ 138.6, 129.4, 128.5, 126.4, 72.7, 44.0, 36.8, 31.8, 25.4, 22.6, 14.0. **HRMS:** (ESI, *m*/*z*): calcd. for $C_{13}H_{20}ONa[M+Na]^+$ 215.1406, found: 215.1404.

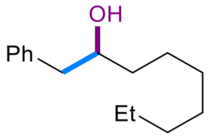

3ab

(Generated following the corresponding general procedure, isolated by column chromatography on silica gel with hexane: EtOAc = 95:5 as eluent, light brown oil, 31.5 mg, yield: 70%) **^{1}H NMR** (500 MHz, $CDCl_3$) δ 7.38 – 7.31 (m, 2H), 7.31 – 7.16 (m, 3H), 3.90 – 3.79 (m, 1H), 2.86 (dd, *J* = 13.6, 4.2 Hz, 1H), 2.67 (dd, *J* = 13.6, 8.4 Hz, 1H), 1.70 – 1.46 (m, 4H), 1.46 – 1.18 (m, 9H), 1.00 – 0.84 (m, 3H). **^{13}C NMR** (126 MHz, $CDCl_3$) δ 138.6, 129.4, 128.5, 126.4, 72.7, 44.0, 36.8, 31.8, 29.6, 29.3, 25.7, 22.6, 14.1. **HRMS:** (ESI, *m*/*z*): calcd. for $C_{15}H_{24}ONa[M+Na]^+$ 243.1719, found: 243.1717.

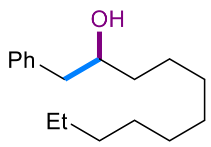

3ac

(Generated following the corresponding general procedure, isolated by column chromatography on silica gel with hexane: EtOAc = 95:5 as eluent, light brown oil, 33 mg, yield: 66%) **^{1}H NMR** (500 MHz, $CDCl_3$) δ 7.41 – 7.30 (t, *J* = 7.4 Hz, 2H), 7.30 – 7.18 (m, 3H), 3.90 – 3.79 (m, 1H), 2.86 (dd, *J* = 13.5, 4.2 Hz, 1H), 2.67 (dd, *J* = 13.5, 8.4 Hz, 1H), 1.68 – 1.46 (m, 4H), 1.45 – 1.16 (m, 13H), 0.92 (t, *J* = 6.9 Hz, 3H). **^{13}C NMR** (126 MHz, $CDCl_3$) δ 138.6, 129.4, 128.5, 126.4, 72.7, 44.0, 36.8, 31.9, 29.6, 29.6, 29.6, 29.3, 25.7, 22.7, 14.1. **HRMS:** (ESI, *m*/*z*): calcd. for $C_{17}H_{28}ONa[M+Na]^+$ 271.2032, found: 271.2038.

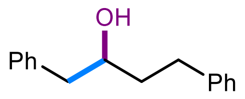

3ad

(Generated following the corresponding general procedure, isolated by column chromatography on silica gel with hexane: EtOAc = 94:6 as eluent, colorless oil, 31 mg, yield: 70%) **^{1}H NMR** (500 MHz, $CDCl_3$) δ 7.43 – 7.18 (m, 10H), 3.94 – 3.83 (m, 1H), 2.97 – 2.84 (m, 2H), 2.83 – 2.68 (m, 2H), 1.97 – 1.80 (m, 2H), 1.60 (s, 1H). **^{13}C NMR** (126 MHz, $CDCl_3$) δ 142.0, 138.3, 129.4, 128.6, 128.4, 128.4, 126.5, 125.8, 71.9, 44.1, 38.4, 32.1. **HRMS:** (ESI, *m*/*z*): calcd. for $C_{16}H_{18}ONa[M+Na]^+$ 249.1250, found: 249.1247.

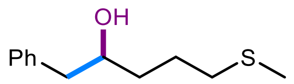

3ae

(Generated following the corresponding general procedure, isolated by column chromatography on silica gel with hexane: EtOAc = 93:7 as eluent, white solid, 28 mg, yield: 67%) **^{1}H NMR** (500 MHz, $CDCl_3$) δ 7.40 – 7.30 (m, 2H), 7.30 – 7.18 (m, 3H), 3.93 – 3.82 (m, 1H), 2.86 (dd, *J* = 13.7, 4.3 Hz, 1H), 2.69 (dd, *J* = 13.2, 8.5 Hz, 1H), 2.55 (t, *J* = 7.0 Hz, 2H), 2.13 (s, 3H), 1.92 – 1.80 (m, 1H), 1.78 – 1.54 (m, 4H). **^{13}C NMR** (126 MHz, $CDCl_3$) δ 138.3, 129.4, 128.6, 126.5, 72.2, 44.2, 35.8, 34.2, 25.3, 15.5. **HRMS:** (ESI, *m*/*z*): calcd. for $C_{12}H_{18}OSNa[M+Na]^+$ 233.0971, found: 233.0967.

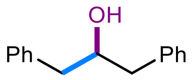

3af

(Generated following the corresponding general procedure, isolated by column chromatography on silica gel with hexane: EtOAc = 94:6 as eluent, colorless oil, 29 mg, yield: 68%) **^{1}H NMR** (500 MHz, $CDCl_3$) δ 7.41 – 7.31 (m, 4H), 7.31 – 7.20 (m, 6H), 4.15 – 4.05 (m, 1H), 2.90 (dd, *J* = 13.5, 4.7 Hz, 2H), 2.80 (dd, *J* = 13.5, 8.2 Hz, 2H), 1.71 (s, 1H). **^{13}C NMR** (126 MHz, $CDCl_3$) δ 138.4, 129.4, 128.5, 126.5, 73.5, 43.3. **HRMS:** (ESI, *m*/*z*): calcd. for $C_{15}H_{16}ONa[M+Na]^+$ 235.1093, found: 235.1092.

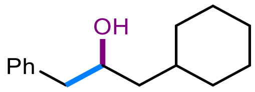

3ag

(Generated following the corresponding general procedure, isolated by column chromatography on silica gel with hexane: EtOAc = 94:6 as eluent, colorless oil, 30 mg, yield: 69%) **^{1}H NMR** (500 MHz, $CDCl_3$) δ 7.42 – 7.30 (m, 2H), 7.30 – 7.18 (m, 3H), 4.00 – 3.92 (m, 1H), 2.84 (dd, *J* = 13.6, 4.2 Hz, 1H), 2.66 (dd, *J* = 13.6, 8.4 Hz, 1H), 1.88 – 1.78 (m, 1H), 1.78 – 1.64 (m, 4H), 1.64 – 1.43 (m, 3H), 1.43 – 1.11 (m, 4H), 1.05 – 0.82 (m, 2H). **^{13}C NMR** (126 MHz, $CDCl_3$) δ 138.6, 129.4, 128.5, 126.4, 70.0, 44.7, 44.6, 34.1, 32.8, 26.6, 26.3, 26.2. **HRMS:** (ESI, *m*/*z*): calcd. for $C_{15}H_{22}ONa[M+Na]^+$ 241.1563, found: 241.1556.

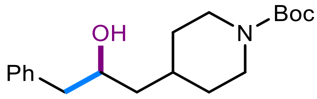

3ah

(Generated following the corresponding general procedure, isolated by column chromatography on silica gel with hexane: EtOAc = 80:20 as eluent, colorless oil, 45 mg, yield: 71%) **^{1}H NMR** (500 MHz, $CDCl_3$) δ 7.39 – 7.30 (m, 2H), 7.30 – 7.16 (m, 3H), 4.08 (s, 2H), 4.00 – 3.84 (m, 1H), 2.82 (dd, *J* = 13.5, 4.2 Hz, 1H), 2.77 – 2.62 (m, 3H), 1.90 – 1.56 (m, 4H), 1.56 – 1.31 (m, 11H), 1.24 – 0.96 (m, 3H). **^{13}C NMR** (126 MHz, $CDCl_3$) δ 154.8, 138.2, 129.3, 128.6, 126.5, 79.2, 69.7, 44.8, 43.5, 32.9, 32.5, 28.4. **HRMS:** (ESI, *m*/*z*): calcd. for $C_{19}H_{29}O_3NNa[M+Na]^+$ 342.2040, found: 342.2041.

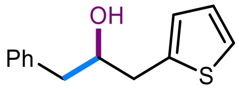

3ai

(Generated following the corresponding general procedure, isolated by column chromatography on silica gel with hexane: EtOAc = 94:6 as eluent, colorless oil, 35 mg, yield: 80%) **^{1}H NMR** (500 MHz, $CDCl_3$) δ 7.44 – 7.32 (m, 2H), 7.32 – 7.24 (m, 3H), 7.24 – 7.18 (m, 1H), 7.05 – 6.97 (m, 1H), 6.97 – 6.87 (m, 1H), 4.18 – 4.02 (m, 1H), 3.11 (dd, *J* = 14.7, 4.4 Hz, 1H), 3.01 (dd, *J* = 14.7, 7.8 Hz, 1H), 2.92 (dd, *J* = 13.6, 4.8 Hz, 1H), 2.80 (dd, *J* = 13.6, 8.0 Hz, 1H), 1.92 (s, 1H). **^{13}C NMR** (126 MHz, $CDCl_3$) δ 140.4, 138.1, 129.4, 128.5, 126.9, 126.5, 126.0, 124.2, 73.2, 43.0, 37.2. **HRMS:** (ESI, *m*/*z*): calcd. for $C_{13}H_{14}OSNa[M+Na]^+$ 241.0658, found: 241.0650.

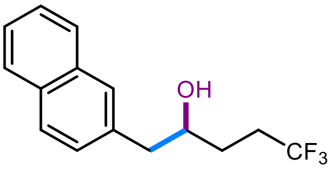

3hj

(Generated following the corresponding general procedure, isolated by column chromatography on silica gel with hexane: EtOAc = 93:7 as eluent, white solid, 32 mg, yield: 63%) **^{1}H NMR** (500 MHz, $CDCl_3$) δ 8.00 – 7.79 (m, 3H), 7.69 (s, 1H), 7.59 – 7.44 (m, 2H), 7.40 – 7.31 (m, 1H), 4.02 – 3.87 (m, 1H), 3.02 (dd, *J* = 13.6, 4.1 Hz, 1H), 2.86 (dd, *J* = 13.6, 8.7 Hz, 1H), 2.51 – 2.34 (m, 1H), 2.31 – 2.13 (m, 1H), 1.97 – 1.85 (m, 1H), 1.85 – 1.72 (m, 1H), 1.67 (s, 1H). **^{13}C NMR** (126 MHz, $CDCl_3$) δ 135.1, 133.6, 132.4, 130.7, 128.5, 128.0, 127.7, 127.5, 127.5, 127.4 (q, *J* = 276.6 Hz), 126.3, 125.8, 71.1, 44.3, 30.4 (q, *J* = 28.9 Hz), 29.0 (q, *J* = 2.7 Hz). **^{19}F NMR** (471 MHz, $CDCl_3$) δ -66.3. **HRMS:** (ESI, *m*/*z*): calcd. for $C_{15}H_{15}OF_3Na[M+Na]^+$ 291.0967, found: 291.0972.

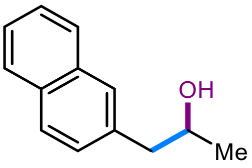

3hk

(Generated following the corresponding general procedure, isolated by column chromatography on silica gel with hexane: EtOAc = 90:10 as eluent, white solid, 24 mg, yield: 67%) **^{1}H NMR** (500 MHz, $CDCl_3$) δ 7.95 – 7.77 (m, 3H), 7.70 (s, 1H), 7.56 – 7.44 (m, 2H), 7.43 – 7.33 (m, 1H), 4.20 – 4.08 (m, 1H), 2.98 (dd, *J* = 13.5, 4.8 Hz, 1H), 2.89 (dd, *J* = 13.5, 8.0 Hz, 1H), 1.67 (s, 1H), 1.32 (d, *J* =6.3 Hz, 3H). **^{13}C NMR** (126 MHz, $CDCl_3$) δ 136.0, 133.5, 132,2, 128.1, 127.8, 127.7, 127.6, 127.5, 126.1, 125.5, 68.7, 45.9, 22.8. **HRMS:** (ESI, *m*/*z*): calcd. for $C_{13}H_{14}ONa[M+Na]^+$ 209.0937, found: 209.0935.

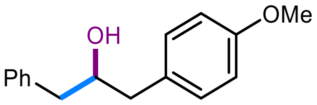

3al

(Generated following the corresponding general procedure, isolated by column chromatography on silica gel with hexane: EtOAc = 92:8 as eluent, colorless oil, 30 mg, yield: 64%) **^{1}H NMR** (500 MHz, $CDCl_3$) δ 7.40 – 7.31 (m, 2H), 7.31 – 7.22 (m, 3H), 7.22 – 7.13 (m, 2H), 6.94 – 6.84 (m, 2H), 6.97 – 6.87 (m, 1H), 4.11 – 3.99 (m, 1H), 3.82 (s, 3H), 2.94 – 2.68 (m, 4H), 1.68 (s, 1H). **^{13}C NMR** (126 MHz, $CDCl_3$) δ 158.3, 138.5, 130.3, 129.4, 128.5, 126.4, 113.9, 73.6, 55.2, 43.3, 42.4. **HRMS:** (ESI, *m*/*z*): calcd. for $C_{16}H_{18}O_2Na[M+Na]^+$ 265.1199, found: 265.1197.

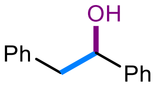

3am

(Generated following the corresponding general procedure, isolated by column chromatography on silica gel with hexane: EtOAc = 95:5 as eluent, white solid, 24 mg, yield: 61%)[2] **^{1}H NMR** (500 MHz, $CDCl_3$) δ 7.42 – 7.37 (m, 4H), 7.37 – 7.30 (m, 3H), 7.30 – 7.26 (m, 1H), 7.26 – 7.20 (m, 2H), 4.93 (dd, *J* = 8.5, 4.9 Hz, 1H), 3.11 – 2.99 (m, 2H), 2.02 (s, 1H). **^{13}C NMR** (126 MHz, $CDCl_3$) δ 143.8, 138.0, 129.5, 128.5, 128.4, 127.6, 126.6, 125.9, 75.3, 46.1. **HRMS:** (ESI, *m*/*z*): calcd. for $C_{14}H_{14}ONa[M+Na]^+$ 221.0937, found: 221.0942.

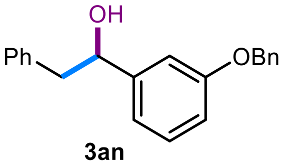

3an

(Generated following the corresponding general procedure, isolated by column chromatography on silica gel with hexane: EtOAc = 92:8 as eluent, white solid, 33 mg, yield: 54%) **^{1}H NMR** (500 MHz, $CDCl_3$) δ 7.56 – 7.41 (m, 4H), 7.41 – 7.18 (m, 7H), 7.11 – 7.04 (m, 1H), 7.04 – 6.89 (m, 2H), 5.09 (s, 2H), 4.91 (dd, *J* = 8.5, 4.8 Hz, 1H), 3.11 – 2.98 (m, 2H), 2.07 (s, 1H). **^{13}C NMR** (126 MHz, $CDCl_3$) δ 158.9, 145.5, 138.0, 137.0, 129.5, 129.4, 128.5, 128.5, 127.9, 127.5, 126.6, 118.5, 114.0, 112.3, 75.2, 69.9, 46.0. **HRMS:** (ESI, *m*/*z*): calcd. for $C_{21}H_{20}O_2Na[M+Na]^+$ 327.1356, found: 327.1360.

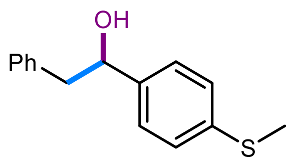

3ao

(Generated following the corresponding general procedure, isolated by column chromatography on silica gel with hexane: EtOAc = 93:7 as eluent, white solid, 26 mg, yield: 53%) **^{1}H NMR** (500 MHz, $CDCl_3$) δ 7.36 – 7.24 (m, 7H), 7.24 – 7.19 (m, 2H), 4.88 (dd, J = 8.1, 5.1 Hz, 1H), 3.09 – 2.95 (m, 2H), 2.51 (s, 3H), 2.01 (s, 1H). **^{13}C NMR** (126 MHz, $CDCl_3$) δ 140.7, 137.8, 137.5, 129.5, 128.5, 126.6, 126.4, 74.9, 45.9, 15.9. **HRMS:** (ESI, *m*/*z*): calcd. for $C_{15}H_{16}OSNa[M+Na]^+$ 267.0814, found: 267.0813.

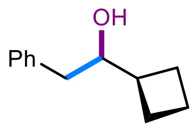

3ap

(Generated following the corresponding general procedure, isolated by column chromatography on silica gel with hexane: EtOAc = 95:5 as eluent, colorless oil, 24 mg, yield: 69%): **^{1}H NMR** (500 MHz, $CDCl_3$) δ 7.39 – 7.31 (m, 2H), 7.31 – 7.18 (m, 3H), 3.83 – 3.70 (m, 1H), 2.79 (dd, J = 13.7, 3.9 Hz, 1H), 2.56 (dd, J = 13.7, 8.4 Hz, 1H), 2.48 – 2.34 (m, 1H), 2.12 – 1.90 (m, 4H), 1.90 – 1.79 (m, 2H), 1.58 (s, 1H). **^{13}C NMR** (126 MHz, $CDCl_3$) δ 138.6, 129.4, 128.4, 126.3, 76.4, 40.9, 40.7, 24.4, 24.2, 17.8. **HRMS:** (ESI, *m*/*z*): calcd. for $C_{12}H_{16}ONa[M+Na]^+$ 199.1093, found: 199.1095.

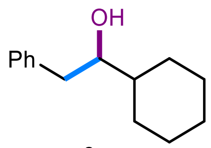

3aq

(Generated following the corresponding general procedure, isolated by column chromatography on silica gel with hexane: EtOAc = 95:5 as eluent, colorless oil, 21 mg, yield: 50%)[1] **^{1}H NMR** (500 MHz, $CDCl_3$) δ 7.44 – 7.31 (m, 2H), 7.31 – 7.16 (m, 3H), 3.65 – 3.56 (m, 1H), 2.91 (dd, J = 13.6, 3.4 Hz, 1H), 2.56 (dd, J = 13.6, 9.6 Hz, 1H), 2.00 – 1.90 (m, 1H), 1.88 – 1.75 (m, 3H), 1.75 – 1.65 (m, 1H), 1.60 – 1.37 (m, 2H), 1.37 – 1.03 (m, 5H). **^{13}C NMR** (126 MHz, $CDCl_3$) δ 139.2, 129.4, 128.5, 126.3, 76.9, 43.2, 40.8, 29.3, 28.0, 26.5, 26.3, 26.1. **HRMS:** (ESI, *m*/*z*): calcd. for $C_{14}H_{20}ONa[M+Na]^+$ 227.1406, found: 227.1415.

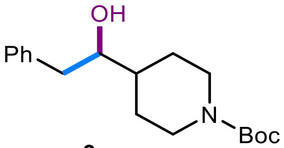

3ar

(Generated following the corresponding general procedure, isolated by column chromatography on silica gel with hexane: EtOAc = 82:18 as eluent, white solid, 31 mg, yield: 50%): **^{1}H NMR** (500 MHz, $CDCl_3$) δ 7.38 – 7.30 (m, 2H), 7.30 – 7.18 (m, 3H), 4.19 (s, 2H), 3.68 – 3.56 (m, 1H), 2.91 (dd, *J* = 13.5, 3.3 Hz, 1H), 2.80 – 2.54 (m, 3H), 1.97 – 1.85 (m, 1H), 1.80 – 1.54 (m, 3H), 1.54 – 1.21 (m, 12H). **^{13}C NMR** (126 MHz, $CDCl_3$) δ 154.8, 138.5, 129.3, 128.6, 126.5, 79.3, 75.9, 43.5, 41.6, 40.8, 28.4. **HRMS:** (ESI, *m*/*z*): calcd. for $C_{18}H_{27}O_3NNa[M+Na]^+$ 328.1883, found: 328.1887.

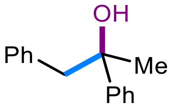

3as

(Generated following the corresponding general procedure, isolated by column chromatography on silica gel with hexane: EtOAc = 96:4 as eluent, colorless oil, 23 mg, yield: 54%)[2] **^{1}H NMR** (500 MHz, $CDCl_3$) δ 7.47 – 7.41 (m, 2H), 7.41 – 7.33 (m, 2H), 7.33 – 7.19 (m, 4H), 7.08 – 6.98 (m, 2H), 3.17 (d, *J* = 13.4 Hz, 1H), 3.06 (d, *J* = 13.4 Hz, 1H), 1.90 (s, 1H), 1.60 (s, 3H). **^{13}C NMR** (126 MHz, $CDCl_3$) δ 147.5, 136.7, 130.6, 128.0, 126.6, 124.9, 74.4, 50.5, 29.4. **HRMS:** (ESI, *m*/*z*): calcd. for $C_{15}H_{16}ONa[M+Na]^+$ 235.1093, found: 235.1096.

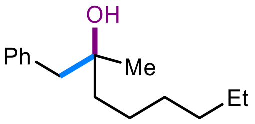

3at

(Generated following the corresponding general procedure, isolated by column chromatography on silica gel with hexane: EtOAc = 96:4 as eluent, colorless oil, 10 mg, yield: 23%) **^{1}H NMR** (500 MHz, $CDCl_3$) δ 7.37 – 7.30 (m, 2H), 7.30 – 7.19 (m, 3H), 2.78 (dd, *J* = 29.4, 13.3 Hz, 2H), 1.55 – 1.40 (m, 4H), 1.40 – 1.24 (m, 7H), 1.16 (s, 3H), 0.92 (t, *J* = 6.9 Hz, 3H). **^{13}C NMR** (126 MHz, $CDCl_3$) δ 137.6, 130.5, 128.2, 126.4, 72.5, 48.0, 41.9, 31.9, 29.8, 26.5, 24.0, 22.6, 14.1. **HRMS:** (ESI, *m*/*z*): calcd. for $C_{15}H_{24}ONa[M+Na]^+$ 243.1719, found: 243.1728.

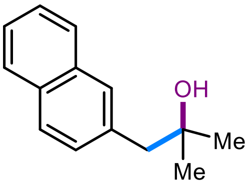

3hv

(Generated following the corresponding general procedure, isolated by column chromatography on silica gel with hexane: EtOAc = 95:5 as eluent, white solid, 34 mg, yield: 85%) **^{1}H NMR** (500 MHz, $CDCl_3$) δ 7.91 – 7.78 (m, 3H), 7.71 (s, 1H), 7.56 – 7.45 (m, 2H), 7.41 (dd, *J* = 8.4, 1.8 Hz, 1H), 2.97 (s, 2H), 1.56 (s, 1H), 1.31 (s, 6H). **^{13}C NMR** (126 MHz, $CDCl_3$) δ 135.4, 133.3, 132.2, 129.0, 128.8, 127.6, 127.6, 126.0, 125.4, 70.9, 49.8, 29.2. **HRMS:** (ESI, *m*/*z*): calcd. for $C_{14}H_{16}ONa[M+Na]^+$ 223.1093, found: 223.1094.

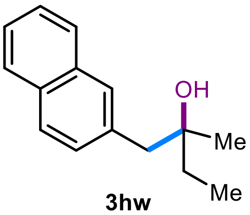

3hw

.

(Generated following the corresponding general procedure, isolated by column chromatography on silica gel with hexane: EtOAc = 95:5 as eluent, white solid, 26 mg, yield: 61%) **^{1}H NMR** (500 MHz, $CDCl_3$) δ 7.92 – 7.77 (m, 3H), 7.71 (s, 1H), 7.57 – 7.45 (m, 2H), 7.41 (dd, *J* = 8.4, 1.8 Hz, 1H), 2.95 (q, *J* = 13.3 Hz, 2H), 1.59 (q, *J* = 7.5 Hz, 2H), 1.47 (s, 1H), 1.21 (s, 3H), 1.04 (t, *J* = 7.5 Hz, 3H). **^{13}C NMR** (126 MHz, $CDCl_3$) δ 135.3, 133.3, 132.2, 129.1, 128.9, 127.6, 126.0, 125.4, 72.9, 47.6, 34.3, 26.0, 8.3. **HRMS:** (ESI, *m*/*z*): calcd. for $C_{15}H_{18}ONa[M+Na]^+$ 237.1250, found: 237.1254.

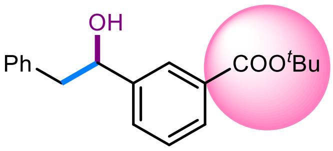

3ax

(Generated following the corresponding general procedure, isolated by column chromatography on silica gel with hexane: EtOAc = 90:10 as eluent, colorless oil, 35 mg, yield: 59%) **^{1}H NMR** (500 MHz, $CDCl_3$) δ 8.00 (s, 1H), 7.93 (d, *J* = 7.7 Hz, 1H), 7.53 (d, *J* = 7.6 Hz, 1H), 7.41 (t, *J* = 7.7 Hz, 1H), 7.37 – 7.14 (m, 5H), 4.97 (dd, *J* = 8.7, 4.7 Hz, 1H), 3.16 – 2.89 (m, 2H), 2.16 (s, 1H), 1.63 (s, 9H). **^{13}C NMR** (126 MHz, $CDCl_3$) δ 165.7, 144.0, 137.7, 132.1, 129.9, 129.5, 128.6, 128.5, 128.2, 126.8, 126.7,

81.1, 74.9, 46.0, 28.2. **HRMS:** (ESI, *m*/*z*): calcd. for $C_{19}H_{22}O_3Na[M+Na]^+$ 321.1461, found: 321.1470.

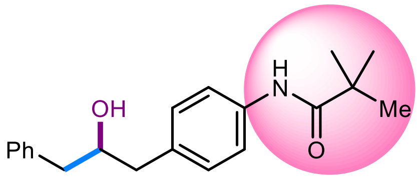

3az

(Generated following the corresponding general procedure, isolated by column chromatography on silica gel with hexane: EtOAc = 65:35 as eluent, white solid, 30 mg, yield: 48%) **^{1}H NMR** (500 MHz, $CDCl_3$) δ 7.56 – 7.45 (m, 2H), 7.44 – 7.30 (m, 3H), 7.31 – 7.13 (m, 5H), 4.12 – 3.98 (m, 1H), 2.85 (ddd, *J* = 14.2, 9.7, 4.7 Hz, 2H), 2.81 – 2.65 (m, 2H), 1.73 (s, 1H), 1.34 (s, 9H). **^{13}C NMR** (126 MHz, $CDCl_3$) δ 176.5, 138.4, 136.4, 134.2, 129.8, 129.4, 128.5, 126.4, 120.2, 73.5, 43.2, 42.7, 39.5, 27.6. **HRMS:** (ESI, *m*/*z*): calcd. for $C_{20}H_{25}O_2NNa[M+Na]^+$ 334.1777, found: 334.1775.

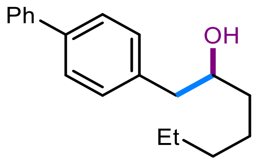

3ba

(Generated following the corresponding general procedure, isolated by column chromatography on silica gel with hexane: EtOAc = 94:6 as eluent, white solid, 40 mg, yield: 75%): **^{1}H NMR** (500 MHz, $CDCl_3$) δ 7.70 – 7.56 (m, 4H), 7.54 – 7.43 (m, 2H), 7.42 – 7.29 (m, 3H), 4.00 – 3.77 (m, 1H), 2.91 (dd, *J* = 13.6, 4.2 Hz, 1H), 2.73 (dd, *J* = 13.6, 8.4 Hz, 1H), 1.73 – 1.50 (m, 4H), 1.50 – 1.19 (m, 5H), 0.95 (t, *J* = 6.9 Hz, 3H). **^{13}C NMR** (126 MHz, $CDCl_3$) δ 140.9, 139.3, 137.7, 129.8, 128.7, 127.2, 127.1, 127.0, 72.7, 43.6, 36.9, 31.8, 25.4, 22.6, 14.0. **HRMS:** (ESI, *m*/*z*): calcd. for $C_{19}H_{24}ONa[M+Na]^+$ 291.1719, found: 291.1730.

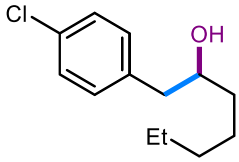

3ca

(Generated following the corresponding general procedure, isolated by column chromatography on silica gel with hexane: EtOAc = 94:6 as eluent, white solid, 29 mg,

yield: 64%): **^{1}H NMR** (500 MHz, $CDCl_3$) δ 7.37 – 7.25 (m, 2H), 7.21 – 7.12 (m, 2H), 3.88 – 3.72 (m, 1H), 2.81 (dd, *J* = 13.7, 4.3 Hz, 1H), 2.65 (dd, *J* = 13.7, 8.3 Hz, 1H), 1.67 – 1.44 (m, 4H), 1.44 – 1.21 (m, 5H), 0.92 (t, *J* = 6.9 Hz, 3H). **^{13}C NMR** (126 MHz, $CDCl_3$) δ 137.2, 132.2, 130.7, 128.6, 72.5, 43.3, 36.8, 31.8, 25.4, 22.6, 14.0. **HRMS:** (ESI, *m/z*): calcd. for $C_{13}H_{19}OClNa[M+Na]^+$ 249.1017, found: 249.1019.

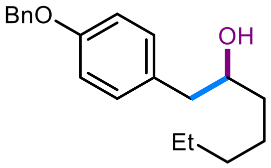

3da

(Generated following the corresponding general procedure, isolated by column chromatography on silica gel with hexane: EtOAc = 94:6 as eluent, white solid, 38 mg, yield: 64%): **^{1}H NMR** (500 MHz, $CDCl_3$) δ 7.54 – 7.32 (m, 5H), 7.17 (d, *J* = 8.5 Hz, 2H), 6.97 (d, *J* = 8.5 Hz, 2H), 5.09 (s, 1H), 3.88 – 3.74 (m, 1H), 2.81 (dd, *J* = 13.7, 4.2 Hz, 1H), 2.62 (dd, *J* = 13.7, 8.4 Hz, 1H), 1.71 – 1.46 (m, 4H), 1.46 – 1.23 (m, 5H), 0.94 (t, *J* = 6.9 Hz, 3H). **^{13}C NMR** (126 MHz, $CDCl_3$) δ 157.5, 137.1, 130.9, 130.3, 128.5, 127.9, 127.4, 114.9, 72.7, 70.0, 43.1, 36.7, 31.8, 25.4, 22.6, 14.0. **HRMS:** (ESI, *m/z*): calcd. for $C_{20}H_{26}O_2Na[M+Na]^+$ 321.1825, found: 321.1825.

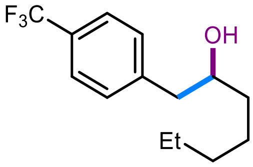

3ea

(Generated following the corresponding general procedure, isolated by column chromatography on silica gel with hexane: EtOAc = 94:6 as eluent, colorless oil, 26 mg, yield: 50%): **^{1}H NMR** (500 MHz, $CDCl_3$) δ 7.59 (d, *J* = 8.0 Hz, 2H), 7.36 (d, *J* = 8.0 Hz, 2H), 3.95 – 3.78 (m, 1H), 2.89 (dd, *J* = 13.6, 4.1 Hz, 1H), 2.75 (dd, *J* = 13.6, 8.3 Hz, 1H), 1.67 – 1.46 (m, 4H), 1.46 – 1.22 (m, 5H), 0.92 (t, *J* = 6.9 Hz, 3H). **^{13}C NMR** (126 MHz, $CDCl_3$) δ 143.0, 129.7, 128.7 (q, *J* = 32.4 Hz), 125.3 (q, *J* = 3.2 Hz), 124.3 (q, *J* = 271.2 Hz), 72.5, 43.7, 37.0, 31.8, 25.3, 22.6, 14.0. **^{19}F NMR** (471 MHz, $CDCl_3$) δ -62.4. **HRMS:** (ESI, *m/z*): calcd. for $C_{14}H_{19}OF_3Na[M+Na]^+$ 283.1280, found: 283.1277.

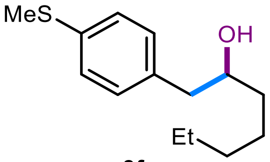

3fa

(Generated following the corresponding general procedure, isolated by column chromatography on silica gel with hexane: EtOAc = 93:7 as eluent, colorless oil, 31 mg, yield: 65%): **^{1}H NMR** (500 MHz, $CDCl_3$) δ 7.24 (d, *J* = 8.2 Hz, 2H), 7.16 (d, *J* = 8.2 Hz, 2H), 3.87 – 3.75 (m, 1H), 2.81 (dd, *J* = 13.6, 4.3 Hz, 1H), 2.63 (dd, *J* = 13.6, 8.3 Hz, 1H), 2.50 (s, 3H), 1.63 – 1.44 (m, 4H), 1.44 – 1.20 (m, 5H), 0.92 (t, *J* = 6.9 Hz, 3H). **^{13}C NMR** (126 MHz, $CDCl_3$) δ 136.1, 135.6, 129.9, 127.0, 72.6, 43.4, 36.8, 31.8, 25.4, 22.6, 16.1, 14.0. **HRMS:** (ESI, *m*/*z*): calcd. for $C_{14}H_{22}OSNa[M+Na]^+$ 261.1284, found: 261.1284.

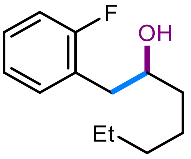

3ga

(Generated following the corresponding general procedure, isolated by column chromatography on silica gel with hexane: EtOAc = 95:5 as eluent, colorless oil, 26 mg, yield: 62%): **^{1}H NMR** (500 MHz, $CDCl_3$) δ 7.33 – 7.16 (m, 2H), 7.16 – 6.97 (m, 2H), 3.96 – 3.80 (m, 1H), 2.92 (dd, *J* = 13.8, 4.4 Hz, 1H), 2.71 (dd, *J* = 13.8, 8.2 Hz, 1H), 2.50 (s, 3H), 1.66 – 1.46 (m, 4H), 1.46 – 1.22 (m, 5H), 0.92 (t, *J* = 6.9 Hz, 3H). **^{13}C NMR** (126 MHz, $CDCl_3$) δ 161.4 (d, *J* = 244.7 Hz), 131.8 (d, *J* = 4.7 Hz), 128.2 (d, *J* = 8.1 Hz), 125.7 (d, *J* = 15.6 Hz), 124.0 (d, *J* = 3.5 Hz), 115.4 (d, *J* = 22.4 Hz), 71.7, 37.2, 37.0, 31.8, 25.4, 22.6, 14.1. **^{19}F NMR** (471 MHz, $CDCl_3$) δ -117.7. **HRMS:** (ESI, *m*/*z*): calcd. for $C_{13}H_{19}OFNa[M+Na]^+$ 233.1312, found: 233.1315.

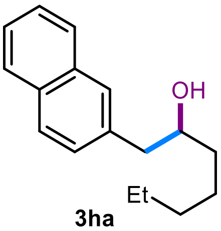

3ha

(Generated following the corresponding general procedure, isolated by column chromatography on silica gel with hexane: EtOAc = 94:6 as eluent, white solid, 33 mg, yield: 68%): **^{1}H NMR** (500 MHz, $CDCl_3$) δ 7.92 – 7.77 (m, 1H), 7.71 (s, 1H), 7.55 – 7.44 (m, 2H), 7.43 – 7.34 (m, 1H), 4.01 – 3.87 (m, 1H), 3.03 (dd, *J* = 13.6, 4.2 Hz, 1H),

2.85 (dd, J = 13.6, 8.4 Hz, 1H), 1.72 – 1.50 (m, 4H), 1.50 – 1.24 (m, 5H), 0.94 (t, J = 6.9 Hz, 3H). **^{13}C NMR** (126 MHz, $CDCl_3$) δ 136.2, 133.5, 132.2, 128.1, 127.8, 127.8, 127.6, 127.5, 126.0, 125.4, 72.6, 44.2, 36.9, 31.8, 25.4, 22.6, 14.0. **HRMS:** (ESI, *m*/*z*): calcd. for $C_{17}H_{22}ONa[M+Na]^+$ 265.1563, found: 265.1564.

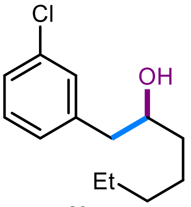

3ia

(Generated following the corresponding general procedure, isolated by column chromatography on silica gel with hexane: EtOAc = 94:6 as eluent, white solid, 26 mg, yield: 57%): **^{1}H NMR** (500 MHz, $CDCl_3$) δ 7.33 – 7.19 (m, 3H), 7.17 – 7.08 (m, 1H), 3.89 – 3.75 (m, 1H), 2.81 (dd, J = 13.7, 4.2 Hz, 1H), 2.65 (dd, J = 13.6, 8.4 Hz, 1H), 1.63 – 1.45 (m, 4H), 1.45 – 1.23 (m, 5H), 0.92 (t, J = 6.9 Hz, 3H). **^{13}C NMR** (126 MHz, $CDCl_3$) δ 140.8, 134.2, 129.7, 129.5, 127.6, 126.6, 72.5, 43.6, 36.9, 31.8, 25.4, 22.6, 14.0. **HRMS:** (ESI, *m*/*z*): calcd. for $C_{13}H_{19}OClNa[M+Na]^+$ 249.1017, found: 249.1020.

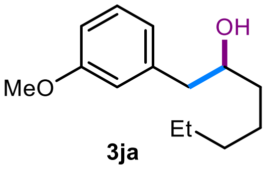

3ja

(Generated following the corresponding general procedure, isolated by column chromatography on silica gel with hexane: EtOAc = 92:8 as eluent, colorless oil, 27 mg, yield: 61%): **^{1}H NMR** (500 MHz, $CDCl_3$) δ 7.32 – 7.20 (m, 1H), 6.90 – 6.71 (m, 3H), 3.89 – 3.76 (m, 4H), 2.83 (dd, J = 13.5, 4.2 Hz, 1H), 2.64 (dd, J = 13.5, 8.5 Hz, 1H), 1.72 – 1.46 (m, 4H), 1.46 – 1.21 (m, 5H), 0.92 (t, J = 6.9 Hz, 3H). **^{13}C NMR** (126 MHz, $CDCl_3$) δ 159.7, 140.2, 129.5, 121.7, 115.1, 111.7, 72.6, 55.1, 44.1, 36.8, 31.8, 25.4, 22.6, 14.0. **HRMS:** (ESI, *m*/*z*): calcd. for $C_{14}H_{22}O_2Na[M+Na]^+$ 245.1512, found: 245.1521.

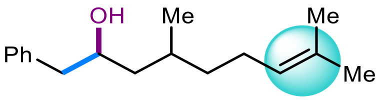

5aa

(Generated following the corresponding general procedure, isolated by column chromatography on silica gel with hexane: EtOAc = 95:5 as eluent, colorless oil, 33 mg, yield: 67%, mixture of two diastereomers, d.r. = 1:1) **^{1}H NMR** (500 MHz, $CDCl_3$) δ

7.43 – 7.31 (m, 2H), 7.31 – 7.15 (m, 3H), 5.23 – 5.05 (m, 1H), 4.02 – 3.88 (m, 1H), 2.94 – 2.77 (m, 1H), 2.77 – 2.58 (m, 1H), 2.13 – 1.90 (m, 2H), 1.80 – 1.12 (m, 12H), 1.06 – 0.81 (m, 3H). **^{13}C NMR** (126 MHz, $CDCl_3$) δ 138.6, 138.6, 131.2, 131.2, 129.4, 128.5, 126.4, 126.4, 124.7, 70.7, 70.3, 44.8, 44.3, 44.2, 44.2, 37.9, 36.6, 29.3, 28.9, 25.7, 25.5, 25.3, 20.2, 19.1, 17.6. **HRMS:** (ESI, *m*/*z*): calcd. for $C_{17}H_{26}ONa[M+Na]^+$ 269.1876, found: 269.1875.

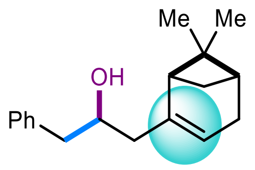

5ab

(Generated following the corresponding general procedure, isolated by column chromatography on silica gel with hexane: EtOAc = 95:5 as eluent, colorless oil, 32 mg, yield: 62%, mixture of two diastereomers, d.r. = 1:1) **^{1}H NMR** (500 MHz, $CDCl_3$) δ 7.41 – 7.30 (m, 2H), 7.30 – 7.16 (m, 3H), 5.48 – 5.34 (m, 1H), 3.93 – 3.78 (m, 1H), 2.88 – 2.73 (m, 2H), 2.48 – 2.38 (m, 1H), 2.38 – 2.11 (m, 5H), 2.11 – 2.00 (m, 1H), 1.92 – 1.74 (m, 1H), 1.30 (d, J = 2.6 Hz, 3H), 1.24 – 1.11 (m, 1H), 0.89 (d, J = 2.2 Hz, 3H). **^{13}C NMR** (126 MHz, $CDCl_3$) δ 145.3, 145.0, 138.8, 138.8, 129.4, 128.4, 128.3, 126.3, 120.3, 120.0, 69.8, 69.6, 45.9, 45.6, 45.0, 44.7, 43.4, 43.4, 40.7, 40.6, 38.0, 37.7, 32.0, 31.7, 31.4, 31.4, 26.2, 26.2, 21.3, 21.2. **HRMS:** (ESI, *m*/*z*): calcd. for $C_{18}H_{24}ONa[M+Na]^+$ 279.1719, found: 279.1728.

VI. NMR spectra of products

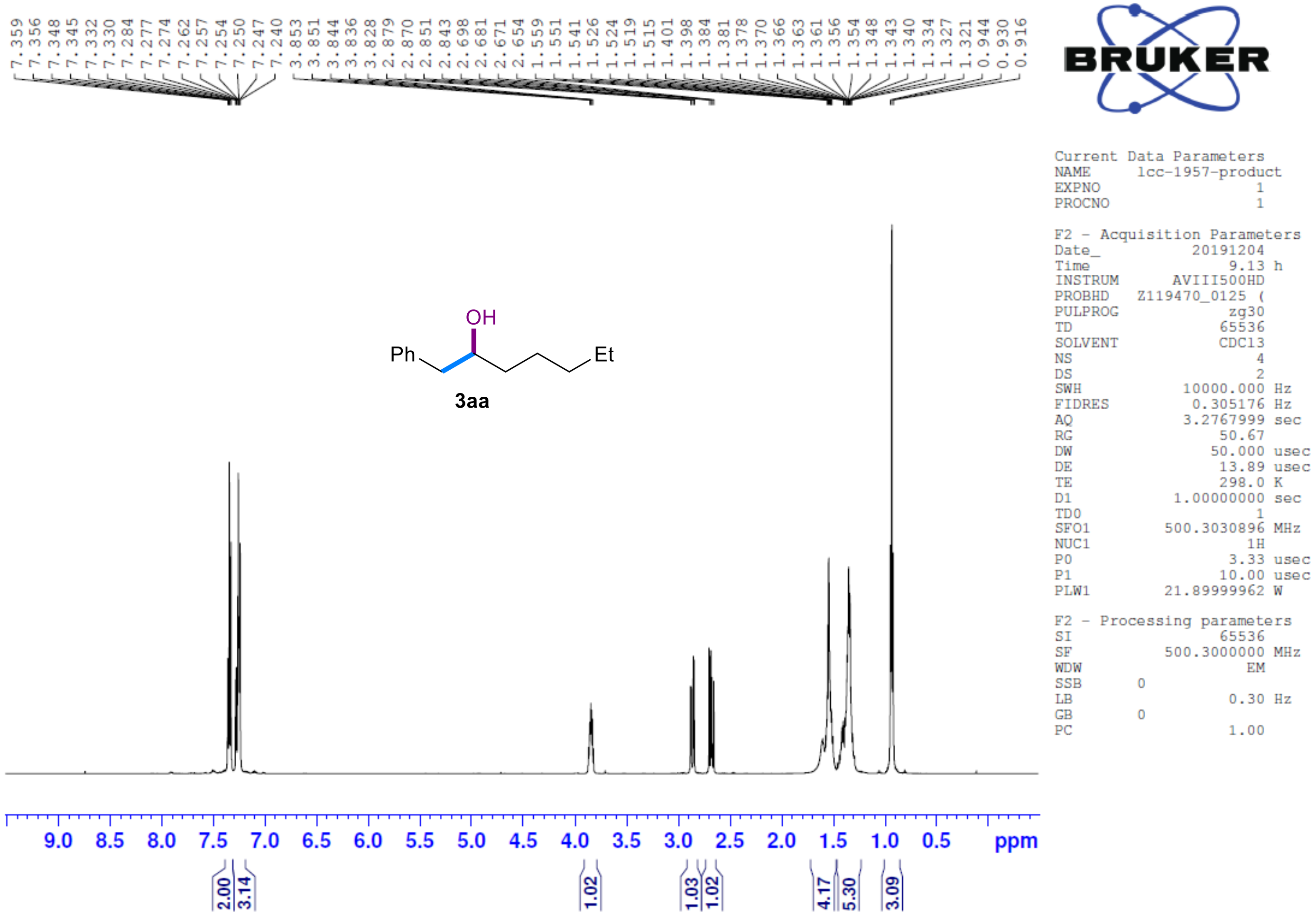
7.359 7.356 7.348 7.345 7.332 7.330 7.284 7.277 7.274 7.262 7.257 7.254 7.250 7.247 7.240 3.853 3.851 3.844 3.836 3.828 2.879 2.870 2.851 2.843 2.698 2.681 2.671 2.654 1.559 1.551 1.541 1.526 1.524 1.519 1.515 1.401 1.398 1.384 1.381 1.378 1.370 1.366 1.363 1.361 1.356 1.354 1.348 1.343 1.340 1.334 1.327 1.321 0.944 0.930 0.916
BRUKER
Current Data Parameters
NAME lcc-1957-product
EXPNO 1
PROCNO 1
F2 - Acquisition Parameters
Date_ 20191204
Time 9.13 h
INSTRUM AVIII500HD
PROBHD Z119470_0125 (
PULPROG zg30
TD 65536
SOLVENT CDCl3
NS 4
DS 2
SWH 10000.000 Hz
FIDRES 0.305176 Hz
AQ 3.2767999 sec
RG 50.67
DW 50.000 usec
DE 13.89 usec
TE 298.0 K
D1 1.00000000 sec
TD0 1
SFO1 500.3030896 MHz
NUC1 1H
P0 3.33 usec
P1 10.00 usec
PLW1 21.89999962 W
F2 - Processing parameters
SI 65536
SF 500.3000000 MHz
WDW EM
SSB 0
LB 0.30 Hz
GB 0
PC 1.00
OH
Ph
Et
3aa
9.0 8.5 8.0 7.5 7.0 6.5 6.0 5.5 5.0 4.5 4.0 3.5 3.0 2.5 2.0 1.5 1.0 0.5 ppm
2.00 3.14 1.02 1.03 1.02 4.17 5.30 3.09

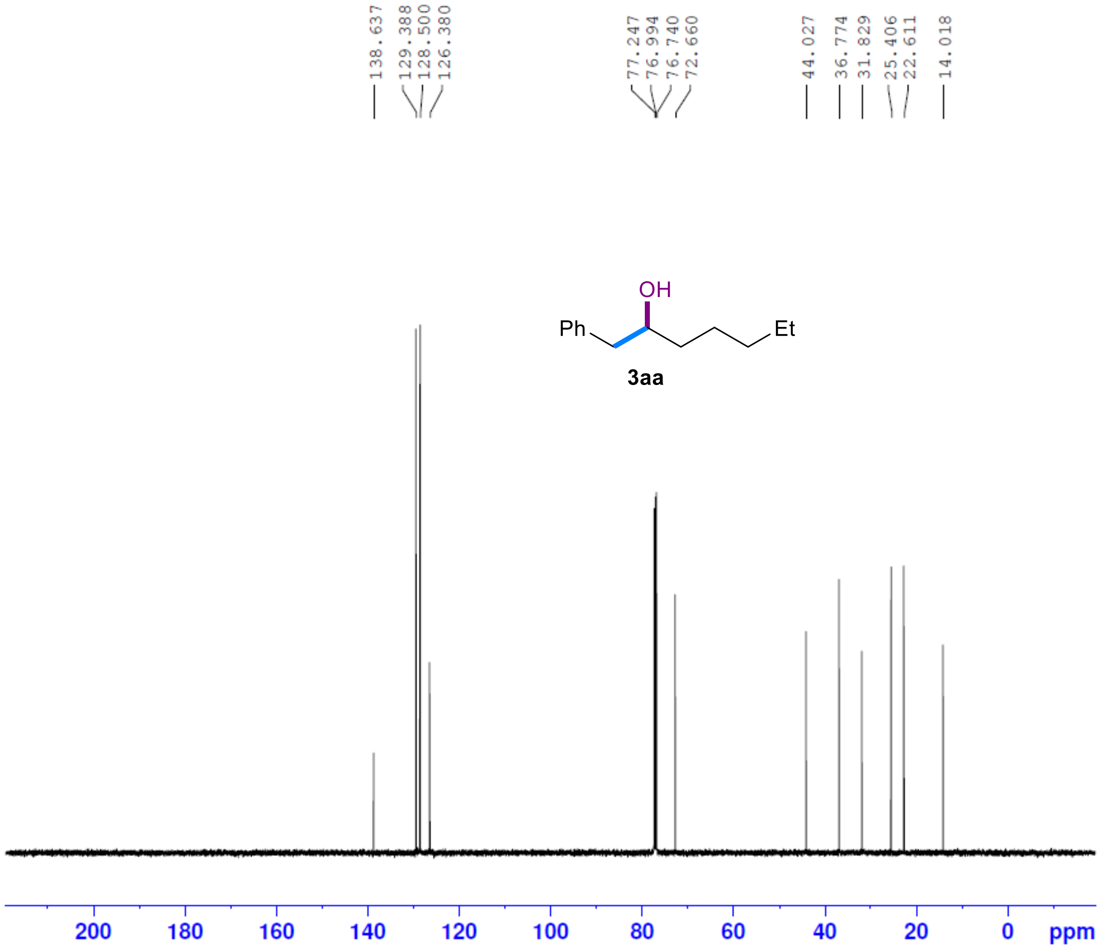


```
Current Data Parameters
NAME       lcc-1957-product
EXPNO                     2
PROCNO                    1

F2 - Acquisition Parameters
Date_              20191204
Time                   9.21 h
INSTRUM          AVIII500HD
PROBHD     Z119470_0125 (
PULPROG              zgpg30
TD                    65536
SOLVENT               CDCl3
NS                      128
DS                        4
SWH               30000.000 Hz
FIDRES             0.915527 Hz
AQ                1.0922667 sec
RG                   192.72
DW                   16.667 usec
DE                    20.34 usec
TE                    298.0 K
D1               2.00000000 sec
D11              0.03000000 sec
TD0                       1
SFO1            125.8131140 MHz
NUC1                    13C
P0                     3.41 usec
P1                    10.23 usec
PLW1            78.00000000 W
SFO2            500.3020012 MHz
NUC2                     1H
CPDPRG[2            waltz16
PCPD2                 80.00 usec
PLW2            21.50000000 W
PLW12            0.49000001 W
PLW13            0.20446000 W

F2 - Processing parameters
SI                    32768
SF              125.8005425 MHz
WDW                      EM
SSB         0
LB                     1.00 Hz
GB          0
PC                     1.40
```

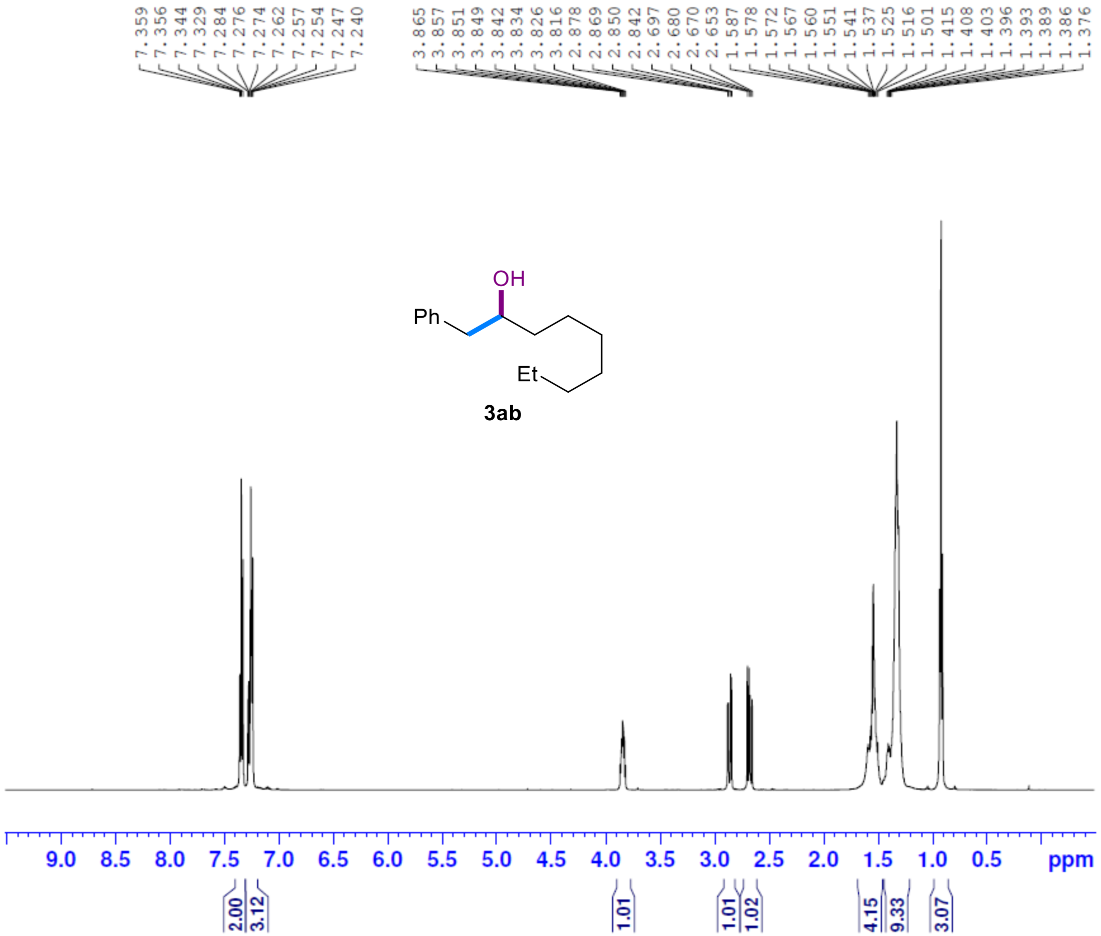

Current Data Parameters
NAME lcc-1958-product
EXPNO 3
PROCNO 1

F2 - Acquisition Parameters
Date_ 20191204
Time 14.05 h
INSTRUM AVIII500HD
PROBHD Z119470_0125 (
PULPROG zg30
TD 65536
SOLVENT CDCl3
NS 4
DS 2
SWH 10000.000 Hz
FIDRES 0.305176 Hz
AQ 3.2767999 sec
RG 44.1
DW 50.000 usec
DE 13.89 usec
TE 298.0 K
D1 1.00000000 sec
TD0 1
SFO1 500.3030896 MHz
NUC1 1H
P0 3.33 usec
P1 10.00 usec
PLW1 21.89999962 W

F2 - Processing parameters
SI 65536
SF 500.3000000 MHz
WDW EM
SSB 0
LB 0.30 Hz
GB 0
PC 1.00

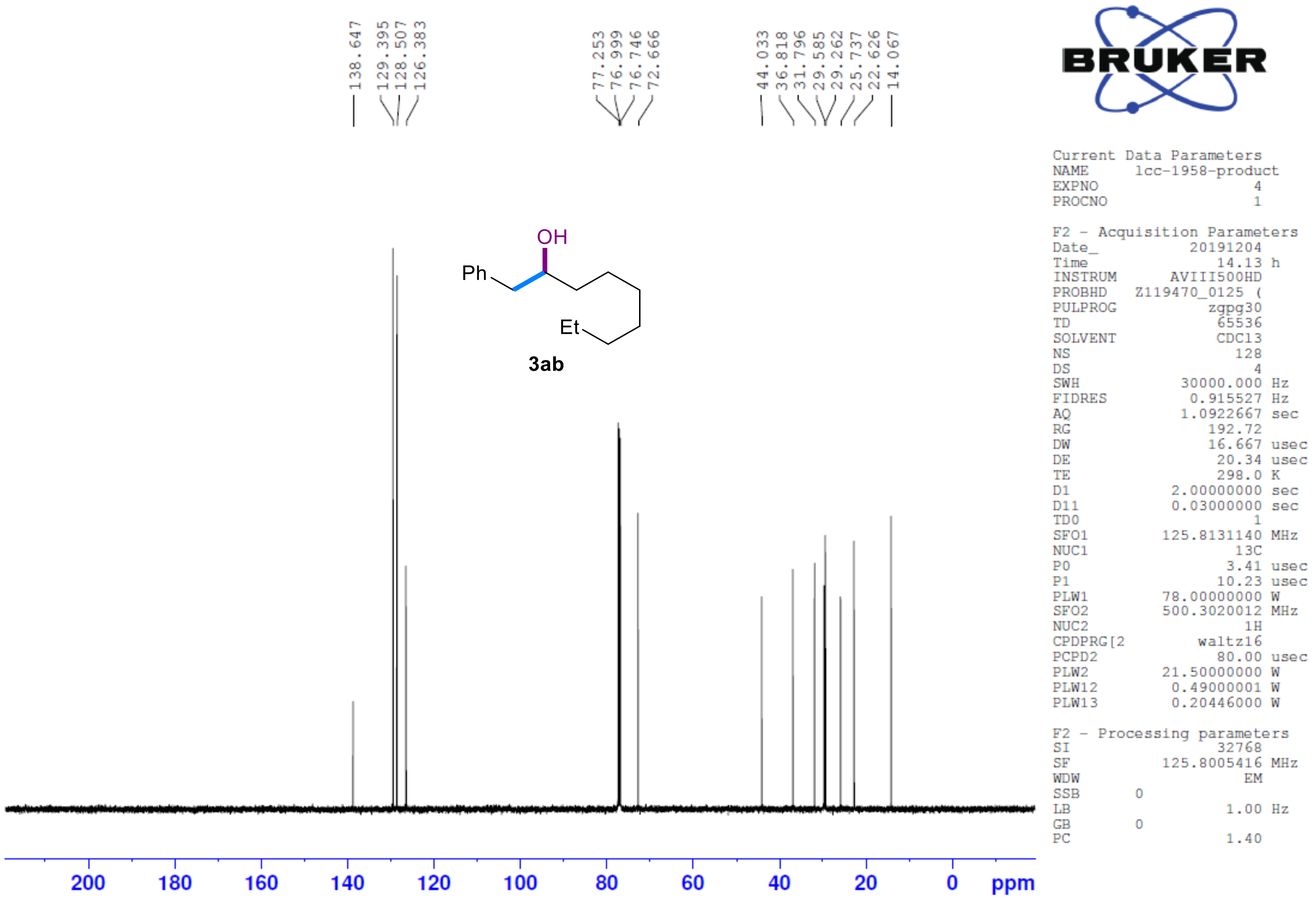
138.647
129.395
128.507
126.383
77.253
76.999
76.746
72.666
44.033
36.818
31.796
29.585
29.262
25.737
22.626
14.067
OH
Ph
Et
3ab
200 180 160 140 120 100 80 60 40 20 0 ppm
BRUKER
Current Data Parameters
NAME lcc-1958-product
EXPNO 4
PROCNO 1
F2 - Acquisition Parameters
Date_ 20191204
Time 14.13 h
INSTRUM AVIII500HD
PROBHD Z119470_0125 (
PULPROG zgpg30
TD 65536
SOLVENT CDCl3
NS 128
DS 4
SWH 30000.000 Hz
FIDRES 0.915527 Hz
AQ 1.0922667 sec
RG 192.72
DW 16.667 usec
DE 20.34 usec
TE 298.0 K
D1 2.00000000 sec
D11 0.03000000 sec
TD0 1
SFO1 125.8131140 MHz
NUC1 13C
P0 3.41 usec
P1 10.23 usec
PLW1 78.00000000 W
SFO2 500.3020012 MHz
NUC2 1H
CPDPRG[2 waltz16
PCPD2 80.00 usec
PLW2 21.50000000 W
PLW12 0.49000001 W
PLW13 0.20446000 W
F2 - Processing parameters
SI 32768
SF 125.8005416 MHz
WDW EM
SSB 0
LB 1.00 Hz
GB 0
PC 1.40

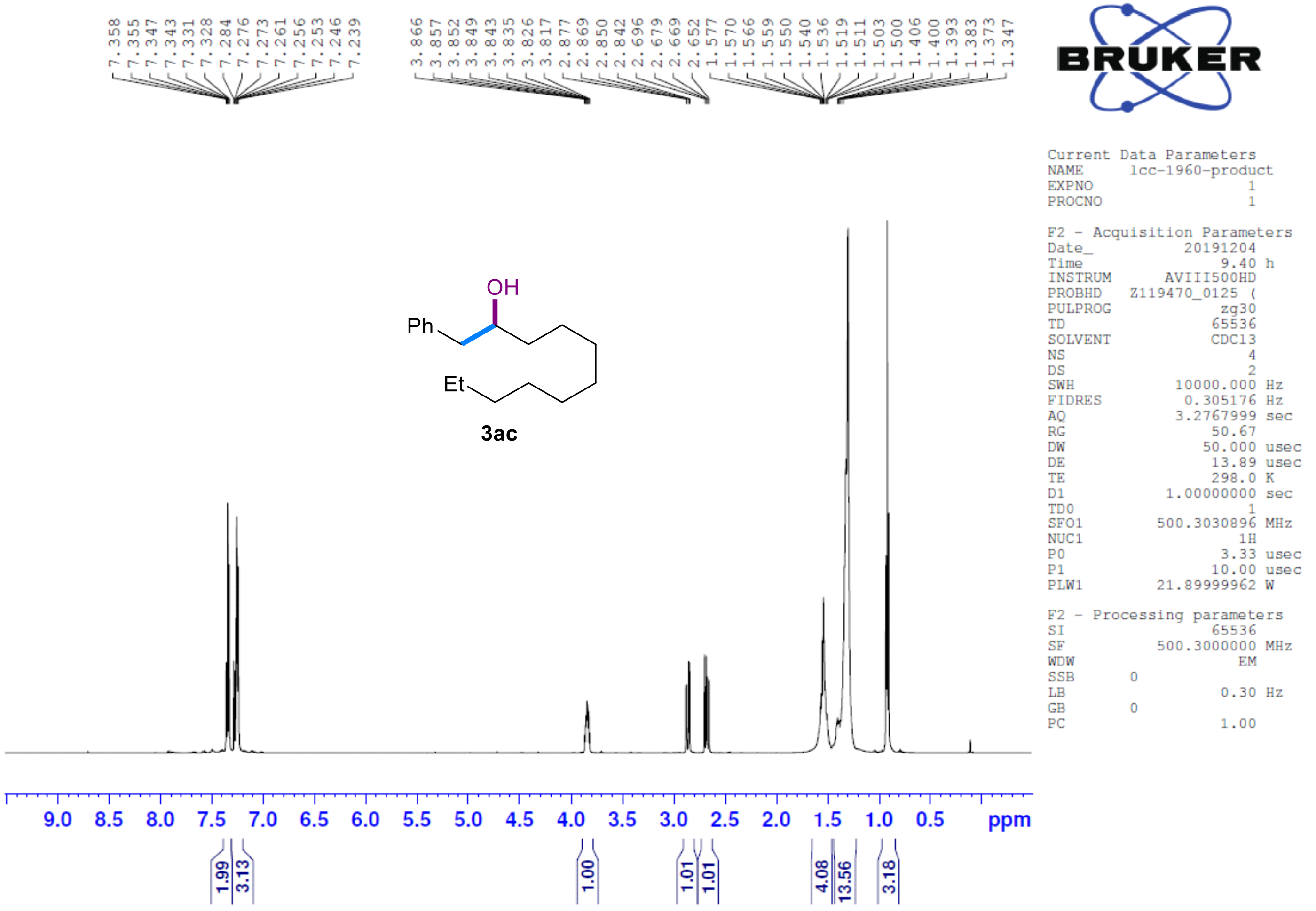
OH
Ph
Et
3ac
Current Data Parameters
NAME lcc-1960-product
EXPNO 1
PROCNO 1
F2 - Acquisition Parameters
Date_ 20191204
Time 9.40 h
INSTRUM AVIII500HD
PROBHD Z119470_0125 (
PULPROG zg30
TD 65536
SOLVENT CDCl3
NS 4
DS 2
SWH 10000.000 Hz
FIDRES 0.305176 Hz
AQ 3.2767999 sec
RG 50.67
DW 50.000 usec
DE 13.89 usec
TE 298.0 K
D1 1.00000000 sec
TD0 1
SFO1 500.3030896 MHz
NUC1 1H
P0 3.33 usec
P1 10.00 usec
PLW1 21.89999962 W
F2 - Processing parameters
SI 65536
SF 500.3000000 MHz
WDW EM
SSB 0
LB 0.30 Hz
GB 0
PC 1.00
ppm

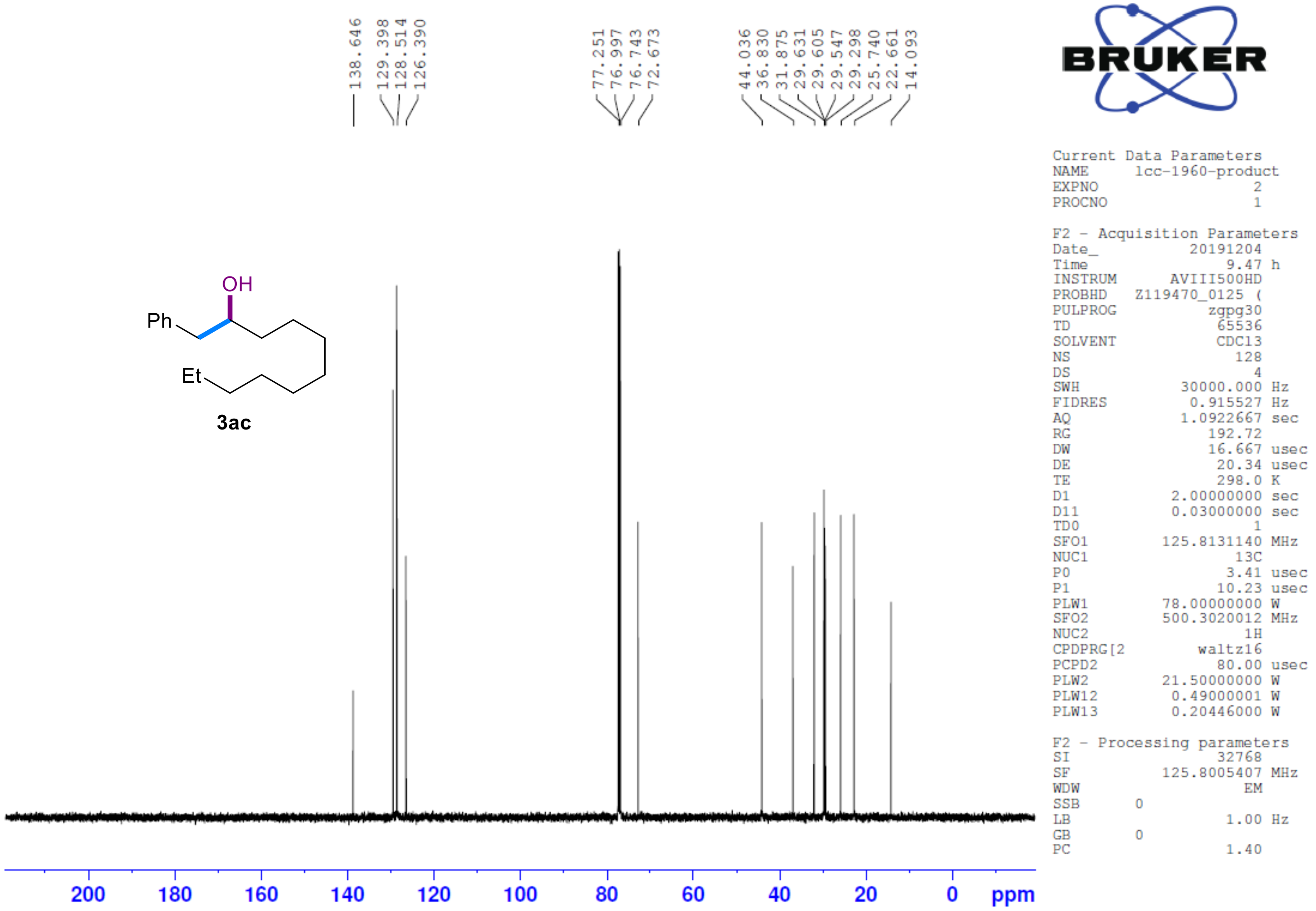
138.646
129.398
128.514
126.390
77.251
76.997
76.743
72.673
44.036
36.830
31.875
29.631
29.605
29.547
29.298
25.740
22.661
14.093
OH
Ph
Et
3ac
BRUKER
Current Data Parameters
NAME lcc-1960-product
EXPNO 2
PROCNO 1
F2 - Acquisition Parameters
Date_ 20191204
Time 9.47 h
INSTRUM AVIII500HD
PROBHD Z119470_0125 (
PULPROG zgpg30
TD 65536
SOLVENT CDCl3
NS 128
DS 4
SWH 30000.000 Hz
FIDRES 0.915527 Hz
AQ 1.0922667 sec
RG 192.72
DW 16.667 usec
DE 20.34 usec
TE 298.0 K
D1 2.00000000 sec
D11 0.03000000 sec
TD0 1
SFO1 125.8131140 MHz
NUC1 13C
P0 3.41 usec
P1 10.23 usec
PLW1 78.00000000 W
SFO2 500.3020012 MHz
NUC2 1H
CPDPRG[2 waltz16
PCPD2 80.00 usec
PLW2 21.50000000 W
PLW12 0.49000001 W
PLW13 0.20446000 W
F2 - Processing parameters
SI 32768
SF 125.8005407 MHz
WDW EM
SSB 0
LB 1.00 Hz
GB 0
PC 1.40
200
180
160
140
120
100
80
60
40
20
0
ppm

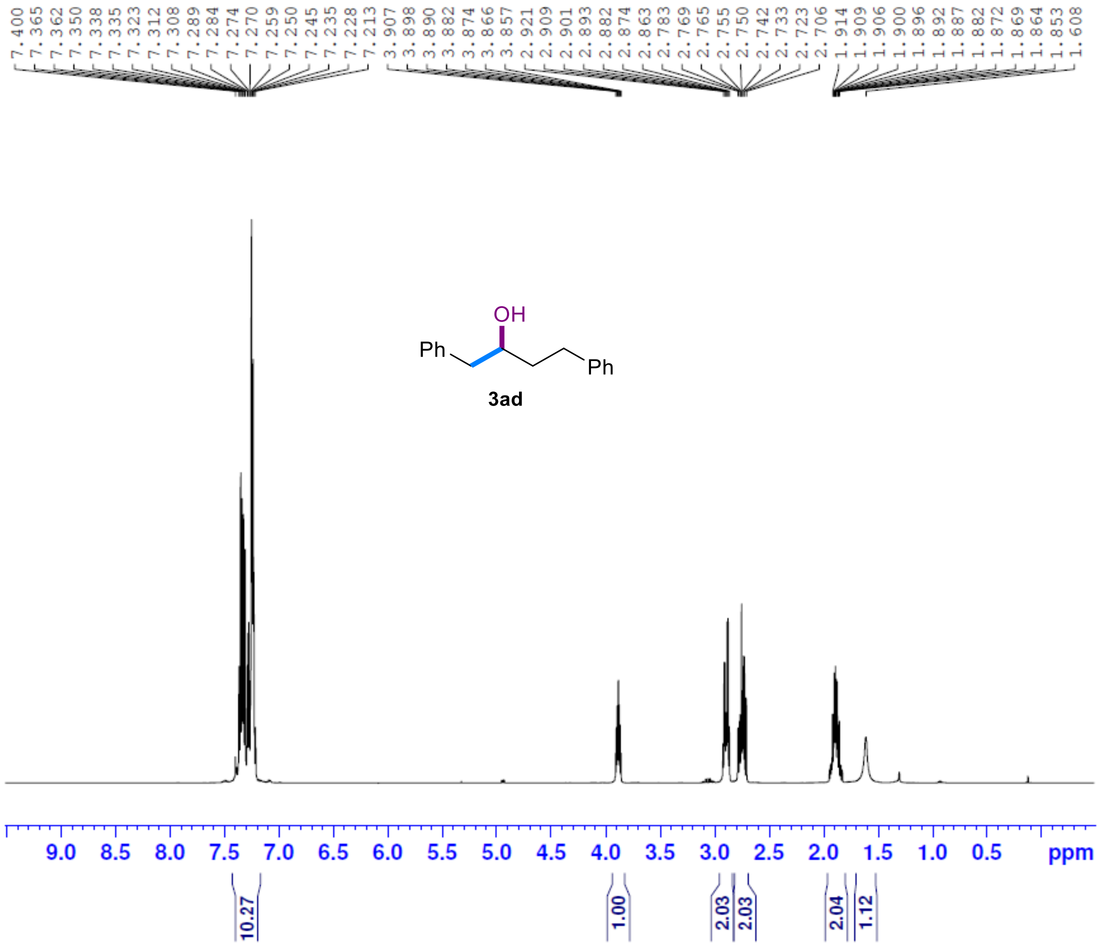

BRUKER

```
Current Data Parameters
NAME      lcc-1959-product
EXPNO                    1
PROCNO                   1

F2 - Acquisition Parameters
Date_             20191204
Time                9.28 h
INSTRUM        AVIII500HD
PROBHD    Z119470_0125 (
PULPROG               zg30
TD                   65536
SOLVENT              CDCl3
NS                       4
DS                       2
SWH             10000.000 Hz
FIDRES           0.305176 Hz
AQ              3.2767999 sec
RG                   77.88
DW                  50.000 usec
DE                   13.89 usec
TE                   298.0 K
D1             1.00000000 sec
TD0                      1
SFO1          500.3030896 MHz
NUC1                    1H
P0                    3.33 usec
P1                   10.00 usec
PLW1           21.89999962 W

F2 - Processing parameters
SI                   65536
SF            500.3000000 MHz
WDW                     EM
SSB      0
LB                    0.30 Hz
GB       0
PC                    1.00
```

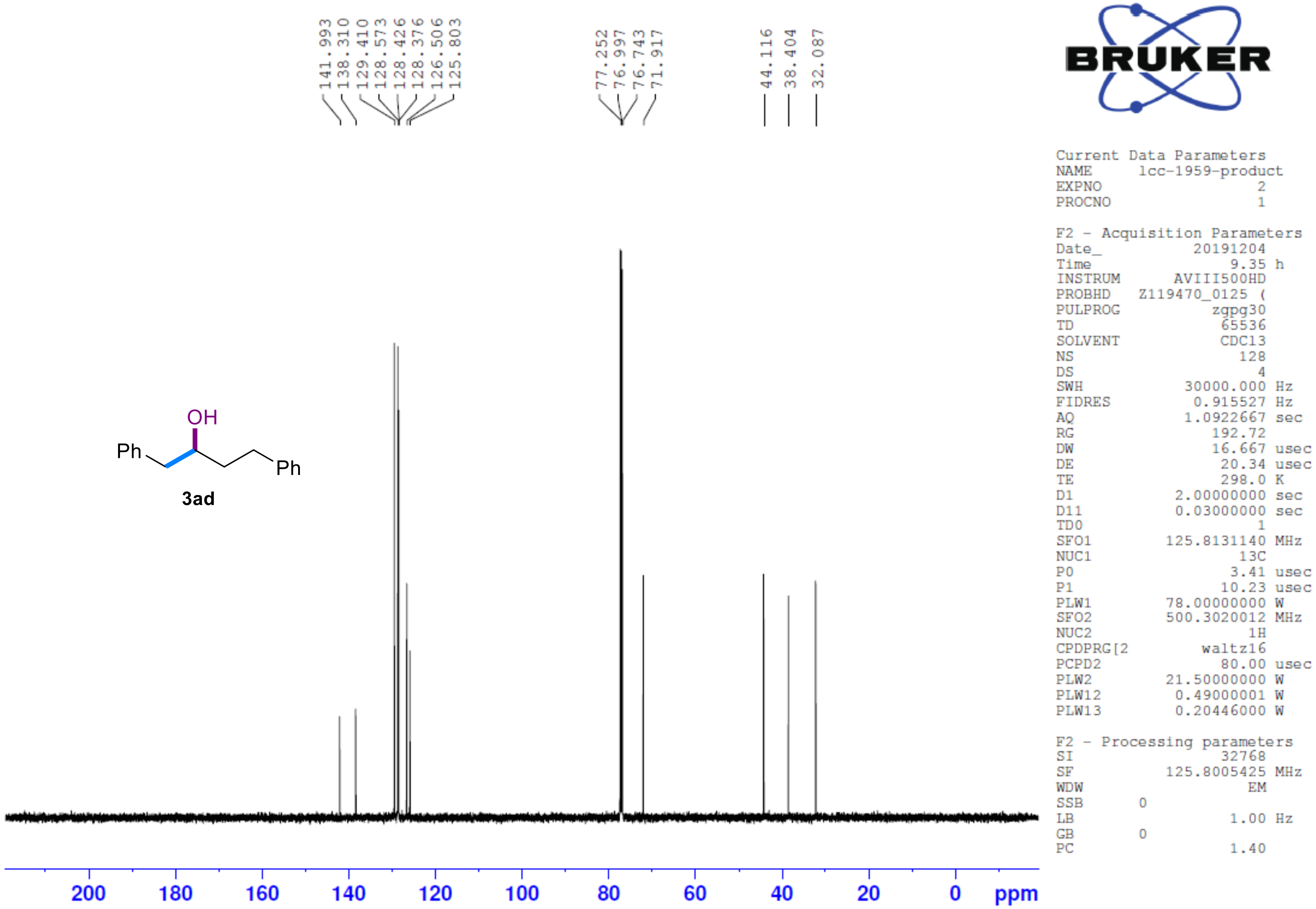
141.993
138.310
129.410
128.573
128.426
128.376
126.506
125.803
77.252
76.997
76.743
71.917
44.116
38.404
32.087
OH
Ph
Ph
3ad
BRUKER
Current Data Parameters
NAME lcc-1959-product
EXPNO 2
PROCNO 1
F2 - Acquisition Parameters
Date_ 20191204
Time 9.35 h
INSTRUM AVIII500HD
PROBHD Z119470_0125 (
PULPROG zgpg30
TD 65536
SOLVENT CDCl3
NS 128
DS 4
SWH 30000.000 Hz
FIDRES 0.915527 Hz
AQ 1.0922667 sec
RG 192.72
DW 16.667 usec
DE 20.34 usec
TE 298.0 K
D1 2.00000000 sec
D11 0.03000000 sec
TD0 1
SFO1 125.8131140 MHz
NUC1 13C
P0 3.41 usec
P1 10.23 usec
PLW1 78.00000000 W
SFO2 500.3020012 MHz
NUC2 1H
CPDPRG[2 waltz16
PCPD2 80.00 usec
PLW2 21.50000000 W
PLW12 0.49000001 W
PLW13 0.20446000 W
F2 - Processing parameters
SI 32768
SF 125.8005425 MHz
WDW EM
SSB 0
LB 1.00 Hz
GB 0
PC 1.40
200
180
160
140
120
100
80
60
40
20
0
ppm

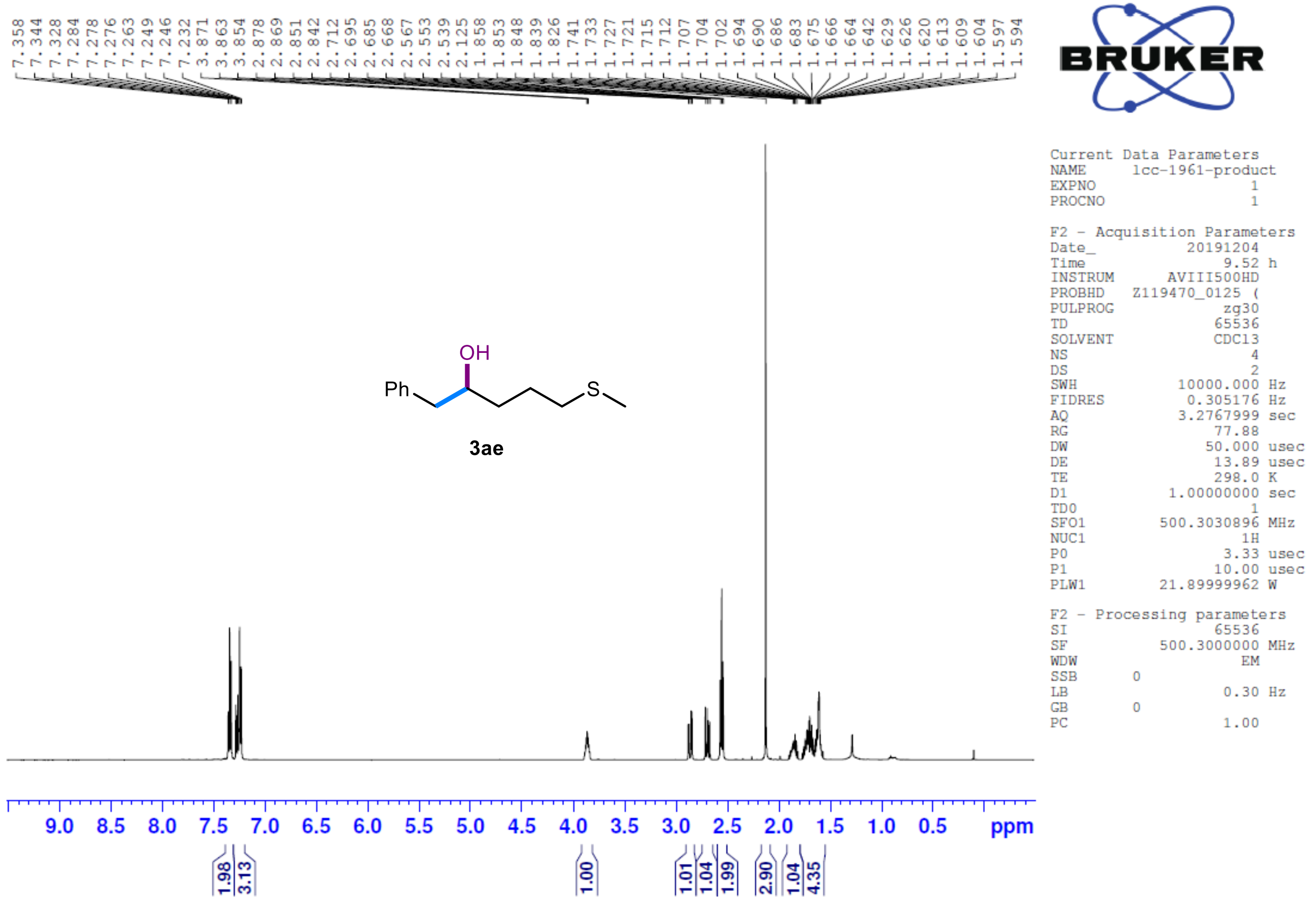
7.358 7.344 7.328 7.284 7.278 7.276 7.263 7.249 7.246 7.232 3.871 3.863 3.854 2.878 2.869 2.851 2.842 2.712 2.695 2.685 2.668 2.567 2.553 2.539 2.125 1.858 1.853 1.848 1.839 1.826 1.741 1.733 1.727 1.721 1.715 1.712 1.707 1.704 1.702 1.694 1.690 1.686 1.683 1.675 1.666 1.664 1.642 1.629 1.626 1.620 1.613 1.609 1.604 1.597 1.594
BRUKER
OH
Ph
S
3ae
Current Data Parameters
NAME lcc-1961-product
EXPNO 1
PROCNO 1
F2 - Acquisition Parameters
Date_ 20191204
Time 9.52 h
INSTRUM AVIII500HD
PROBHD Z119470_0125 (
PULPROG zg30
TD 65536
SOLVENT CDCl3
NS 4
DS 2
SWH 10000.000 Hz
FIDRES 0.305176 Hz
AQ 3.2767999 sec
RG 77.88
DW 50.000 usec
DE 13.89 usec
TE 298.0 K
D1 1.00000000 sec
TD0 1
SFO1 500.3030896 MHz
NUC1 1H
P0 3.33 usec
P1 10.00 usec
PLW1 21.89999962 W
F2 - Processing parameters
SI 65536
SF 500.3000000 MHz
WDW EM
SSB 0
LB 0.30 Hz
GB 0
PC 1.00
9.0 8.5 8.0 7.5 7.0 6.5 6.0 5.5 5.0 4.5 4.0 3.5 3.0 2.5 2.0 1.5 1.0 0.5 ppm
1.98 3.13 1.00 1.01 1.04 1.99 2.90 1.04 4.35

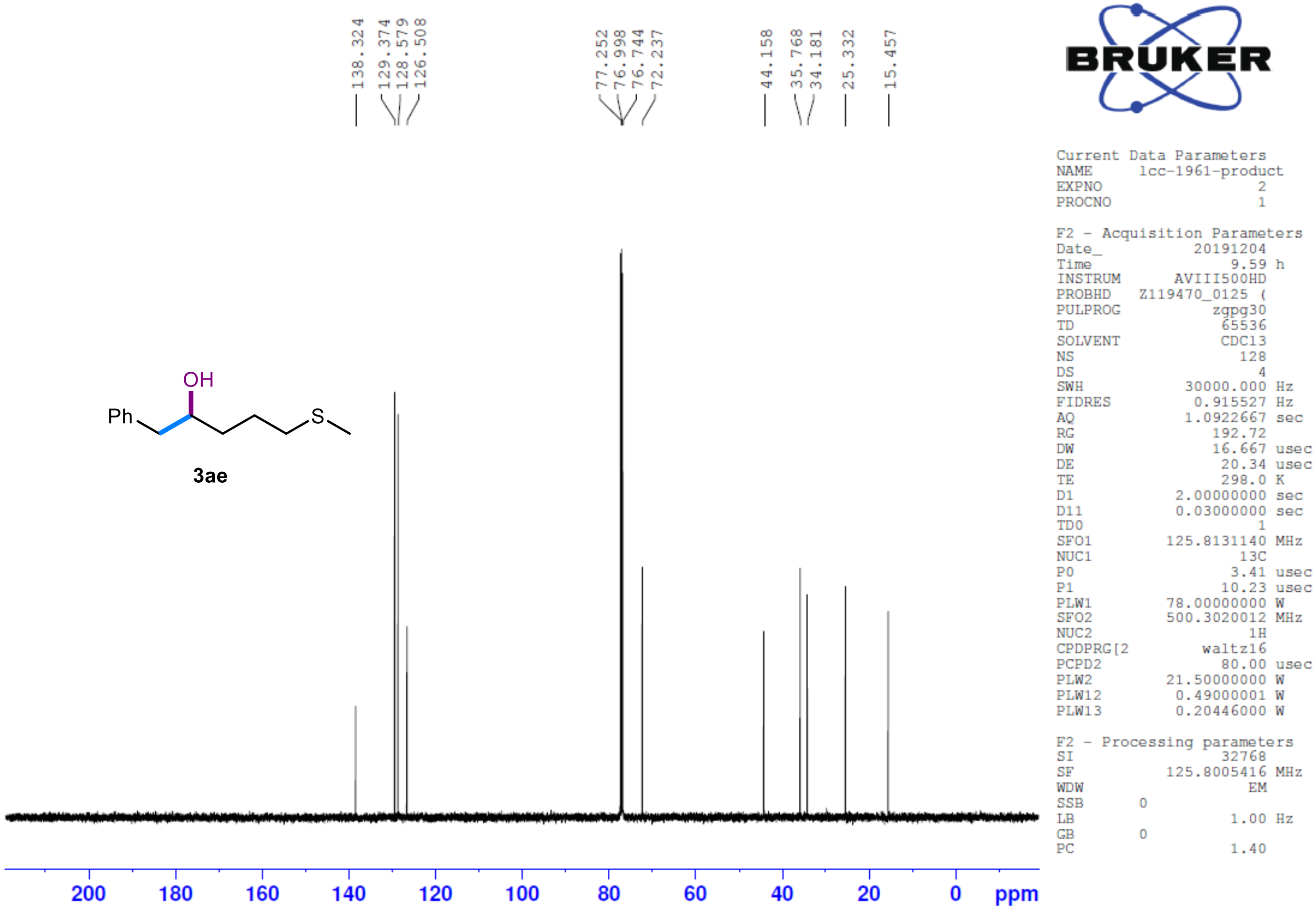
138.324
129.374
128.579
126.508
77.252
76.998
76.744
72.237
44.158
35.768
34.181
25.332
15.457
OH
Ph
S
3ae
200
180
160
140
120
100
80
60
40
20
0
ppm
BRUKER
Current Data Parameters
NAME lcc-1961-product
EXPNO 2
PROCNO 1
F2 - Acquisition Parameters
Date_ 20191204
Time 9.59 h
INSTRUM AVIII500HD
PROBHD Z119470_0125 (
PULPROG zgpg30
TD 65536
SOLVENT CDCl3
NS 128
DS 4
SWH 30000.000 Hz
FIDRES 0.915527 Hz
AQ 1.0922667 sec
RG 192.72
DW 16.667 usec
DE 20.34 usec
TE 298.0 K
D1 2.00000000 sec
D11 0.03000000 sec
TD0 1
SFO1 125.8131140 MHz
NUC1 13C
P0 3.41 usec
P1 10.23 usec
PLW1 78.00000000 W
SFO2 500.3020012 MHz
NUC2 1H
CPDPRG[2 waltz16
PCPD2 80.00 usec
PLW2 21.50000000 W
PLW12 0.49000001 W
PLW13 0.20446000 W
F2 - Processing parameters
SI 32768
SF 125.8005416 MHz
WDW EM
SSB 0
LB 1.00 Hz
GB 0
PC 1.40

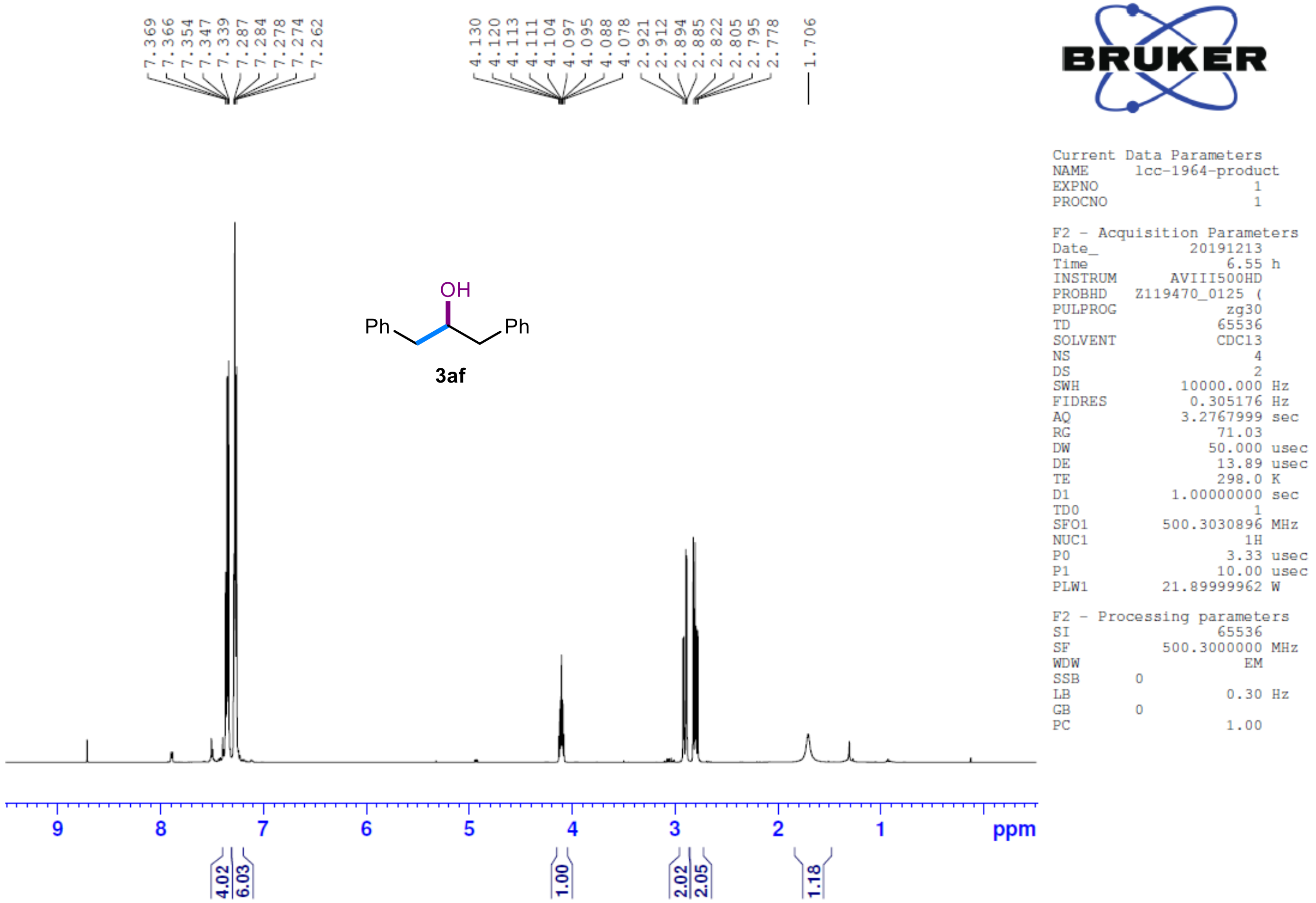
7.369
7.366
7.354
7.347
7.339
7.287
7.284
7.278
7.274
7.262
4.130
4.120
4.113
4.111
4.104
4.097
4.095
4.088
4.078
2.921
2.912
2.894
2.885
2.822
2.805
2.795
2.778
1.706
OH
Ph
Ph
3af
9
8
7
6
5
4
3
2
1
ppm
4.02
6.03
1.00
2.02
2.05
1.18
Current Data Parameters
NAME lcc-1964-product
EXPNO 1
PROCNO 1
F2 - Acquisition Parameters
Date_ 20191213
Time 6.55 h
INSTRUM AVIII500HD
PROBHD Z119470_0125 (
PULPROG zg30
TD 65536
SOLVENT CDCl3
NS 4
DS 2
SWH 10000.000 Hz
FIDRES 0.305176 Hz
AQ 3.2767999 sec
RG 71.03
DW 50.000 usec
DE 13.89 usec
TE 298.0 K
D1 1.00000000 sec
TD0 1
SFO1 500.3030896 MHz
NUC1 1H
P0 3.33 usec
P1 10.00 usec
PLW1 21.89999962 W
F2 - Processing parameters
SI 65536
SF 500.3000000 MHz
WDW EM
SSB 0
LB 0.30 Hz
GB 0
PC 1.00

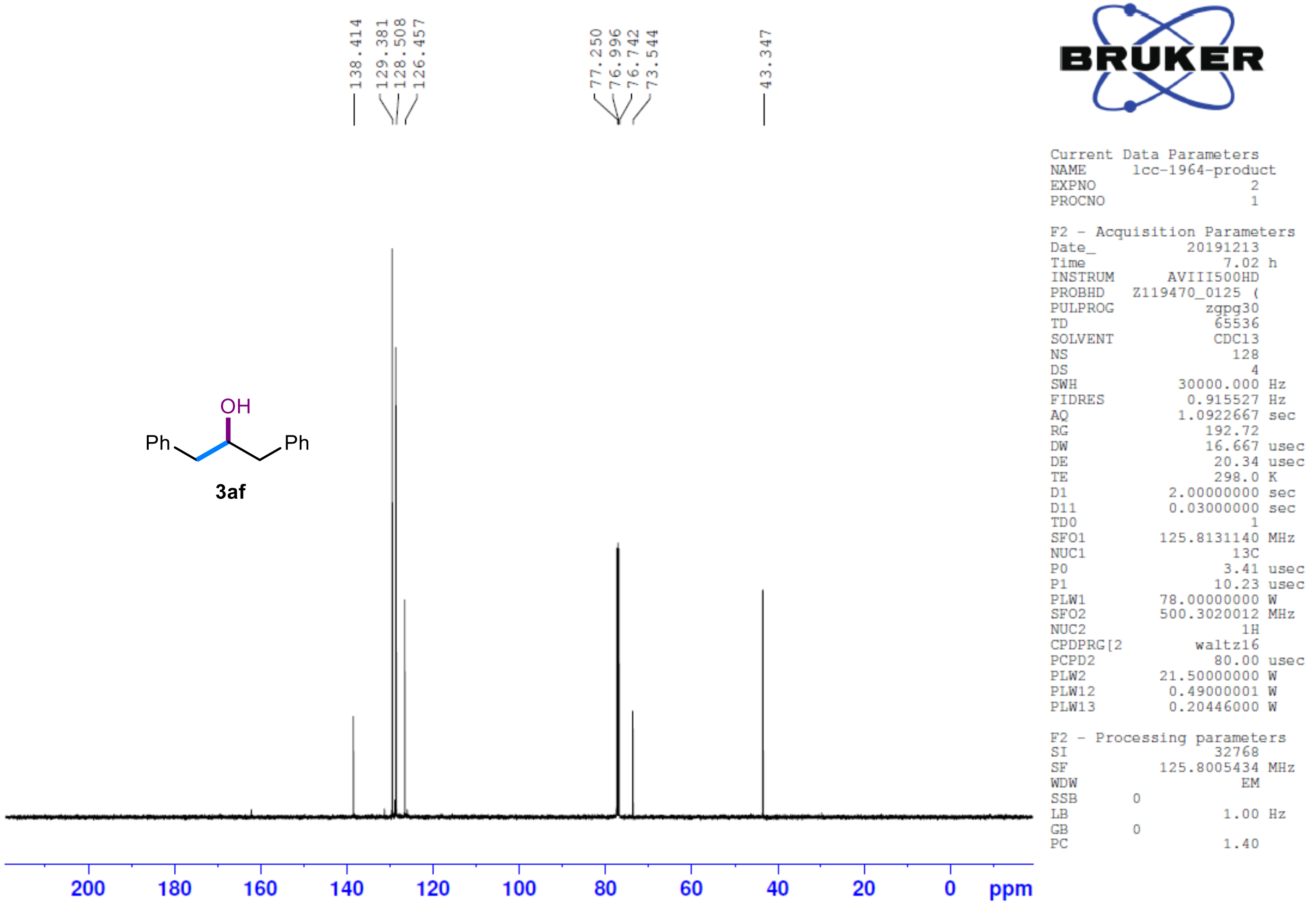
138.414
129.381
128.508
126.457
77.250
76.996
76.742
73.544
43.347
OH
Ph
Ph
3af
200
180
160
140
120
100
80
60
40
20
0
ppm
BRUKER
Current Data Parameters
NAME lcc-1964-product
EXPNO 2
PROCNO 1
F2 - Acquisition Parameters
Date_ 20191213
Time 7.02 h
INSTRUM AVIII500HD
PROBHD Z119470_0125 (
PULPROG zgpg30
TD 65536
SOLVENT CDCl3
NS 128
DS 4
SWH 30000.000 Hz
FIDRES 0.915527 Hz
AQ 1.0922667 sec
RG 192.72
DW 16.667 usec
DE 20.34 usec
TE 298.0 K
D1 2.00000000 sec
D11 0.03000000 sec
TD0 1
SFO1 125.8131140 MHz
NUC1 13C
P0 3.41 usec
P1 10.23 usec
PLW1 78.00000000 W
SFO2 500.3020012 MHz
NUC2 1H
CPDPRG[2 waltz16
PCPD2 80.00 usec
PLW2 21.50000000 W
PLW12 0.49000001 W
PLW13 0.20446000 W
F2 - Processing parameters
SI 32768
SF 125.8005434 MHz
WDW EM
SSB 0
LB 1.00 Hz
GB 0
PC 1.40

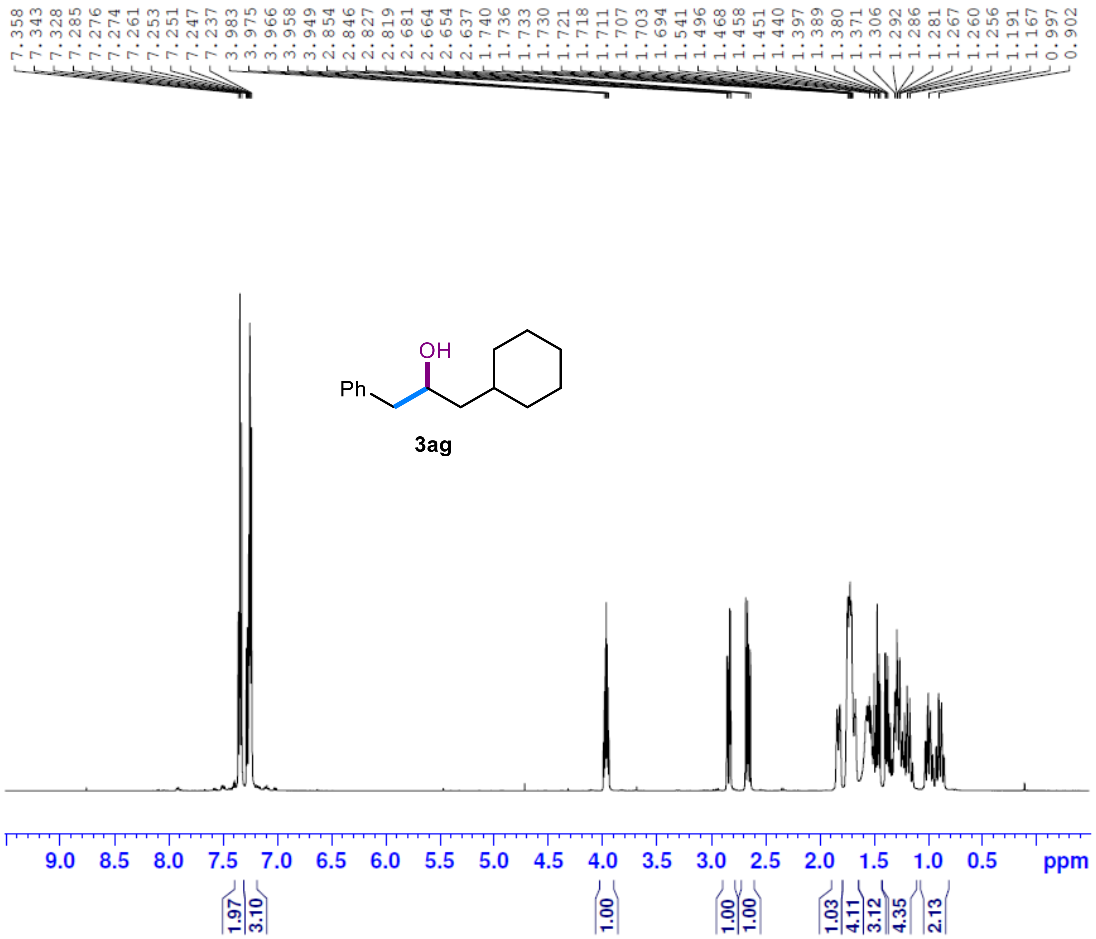


```
Current Data Parameters
NAME       lcc-1983-product
EXPNO                     1
PROCNO                    1

F2 - Acquisition Parameters
Date_              20200102
Time                  11.11 h
INSTRUM          AVIII500HD
PROBHD     Z119470_0125 (
PULPROG                zg30
TD                    65536
SOLVENT               CDCl3
NS                        4
DS                        2
SWH               10000.000 Hz
FIDRES             0.305176 Hz
AQ                3.2767999 sec
RG                    50.67
DW                   50.000 usec
DE                    13.89 usec
TE                    298.0 K
D1               1.00000000 sec
TD0                       1
SFO1            500.3030896 MHz
NUC1                     1H
P0                     3.33 usec
P1                    10.00 usec
PLW1            21.89999962 W

F2 - Processing parameters
SI                    65536
SF              500.3000000 MHz
WDW                      EM
SSB        0
LB                     0.30 Hz
GB         0
PC                     1.00
```

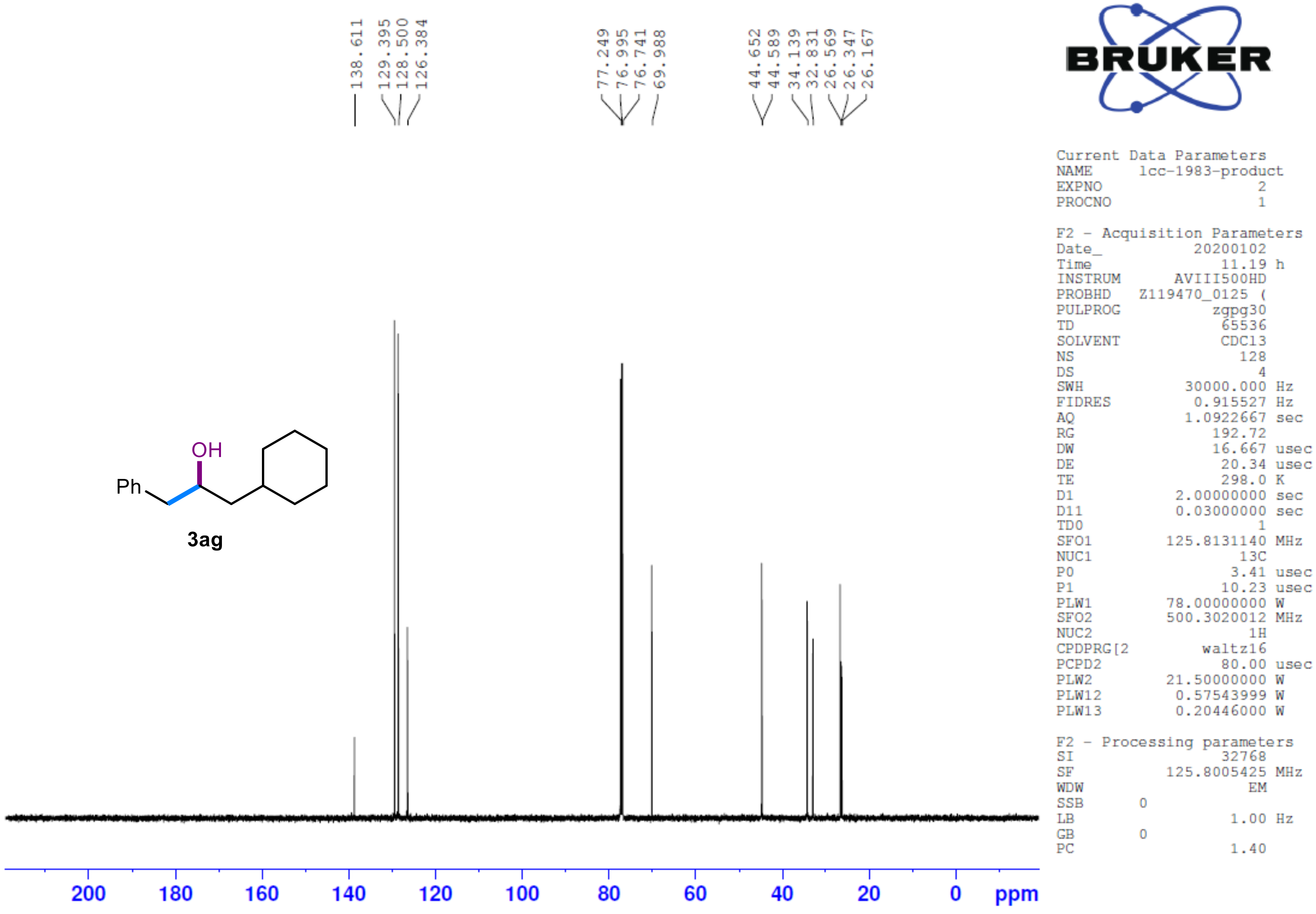
138.611
129.395
128.500
126.384
77.249
76.995
76.741
69.988
44.652
44.589
34.139
32.831
26.569
26.347
26.167
OH
Ph
3ag
200
180
160
140
120
100
80
60
40
20
0
ppm
BRUKER
Current Data Parameters
NAME lcc-1983-product
EXPNO 2
PROCNO 1
F2 - Acquisition Parameters
Date_ 20200102
Time 11.19 h
INSTRUM AVIII500HD
PROBHD Z119470_0125 (
PULPROG zgpg30
TD 65536
SOLVENT CDCl3
NS 128
DS 4
SWH 30000.000 Hz
FIDRES 0.915527 Hz
AQ 1.0922667 sec
RG 192.72
DW 16.667 usec
DE 20.34 usec
TE 298.0 K
D1 2.00000000 sec
D11 0.03000000 sec
TD0 1
SFO1 125.8131140 MHz
NUC1 13C
P0 3.41 usec
P1 10.23 usec
PLW1 78.00000000 W
SFO2 500.3020012 MHz
NUC2 1H
CPDPRG[2 waltz16
PCPD2 80.00 usec
PLW2 21.50000000 W
PLW12 0.57543999 W
PLW13 0.20446000 W
F2 - Processing parameters
SI 32768
SF 125.8005425 MHz
WDW EM
SSB 0
LB 1.00 Hz
GB 0
PC 1.40

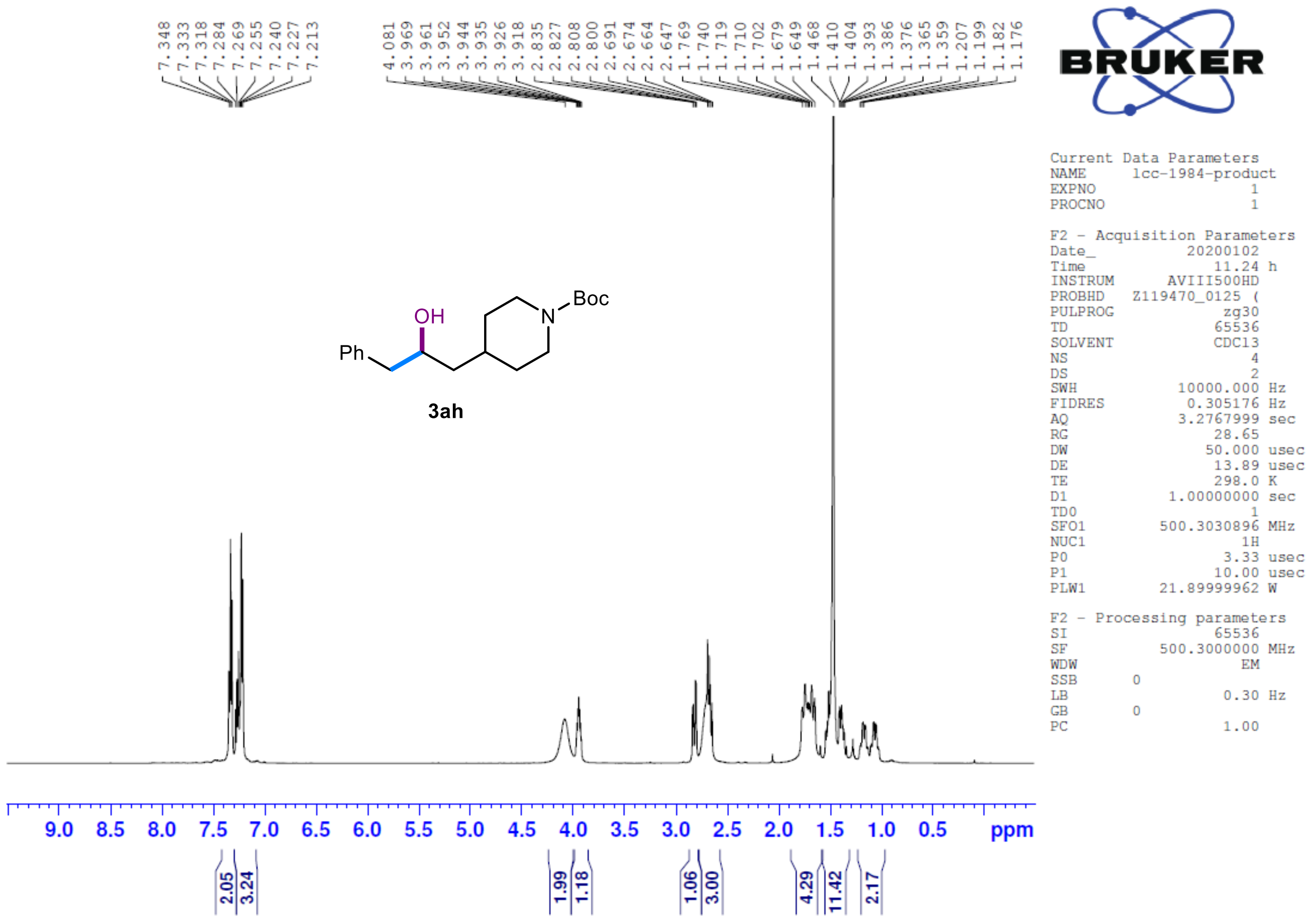
7.348
7.333
7.318
7.284
7.269
7.255
7.240
7.227
7.213
4.081
3.969
3.961
3.952
3.944
3.935
3.926
3.918
2.835
2.827
2.808
2.800
2.691
2.674
2.664
2.647
1.769
1.740
1.719
1.710
1.702
1.679
1.649
1.468
1.410
1.404
1.393
1.386
1.376
1.365
1.359
1.207
1.199
1.182
1.176
BRUKER
Current Data Parameters
NAME lcc-1984-product
EXPNO 1
PROCNO 1
F2 - Acquisition Parameters
Date_ 20200102
Time 11.24 h
INSTRUM AVIII500HD
PROBHD Z119470_0125 (
PULPROG zg30
TD 65536
SOLVENT CDCl3
NS 4
DS 2
SWH 10000.000 Hz
FIDRES 0.305176 Hz
AQ 3.2767999 sec
RG 28.65
DW 50.000 usec
DE 13.89 usec
TE 298.0 K
D1 1.00000000 sec
TD0 1
SFO1 500.3030896 MHz
NUC1 1H
P0 3.33 usec
P1 10.00 usec
PLW1 21.89999962 W
F2 - Processing parameters
SI 65536
SF 500.3000000 MHz
WDW EM
SSB 0
LB 0.30 Hz
GB 0
PC 1.00
OH
Boc
N
Ph
3ah
9.0 8.5 8.0 7.5 7.0 6.5 6.0 5.5 5.0 4.5 4.0 3.5 3.0 2.5 2.0 1.5 1.0 0.5 ppm
2.05
3.24
1.99
1.18
1.06
3.00
4.29
11.42
2.17

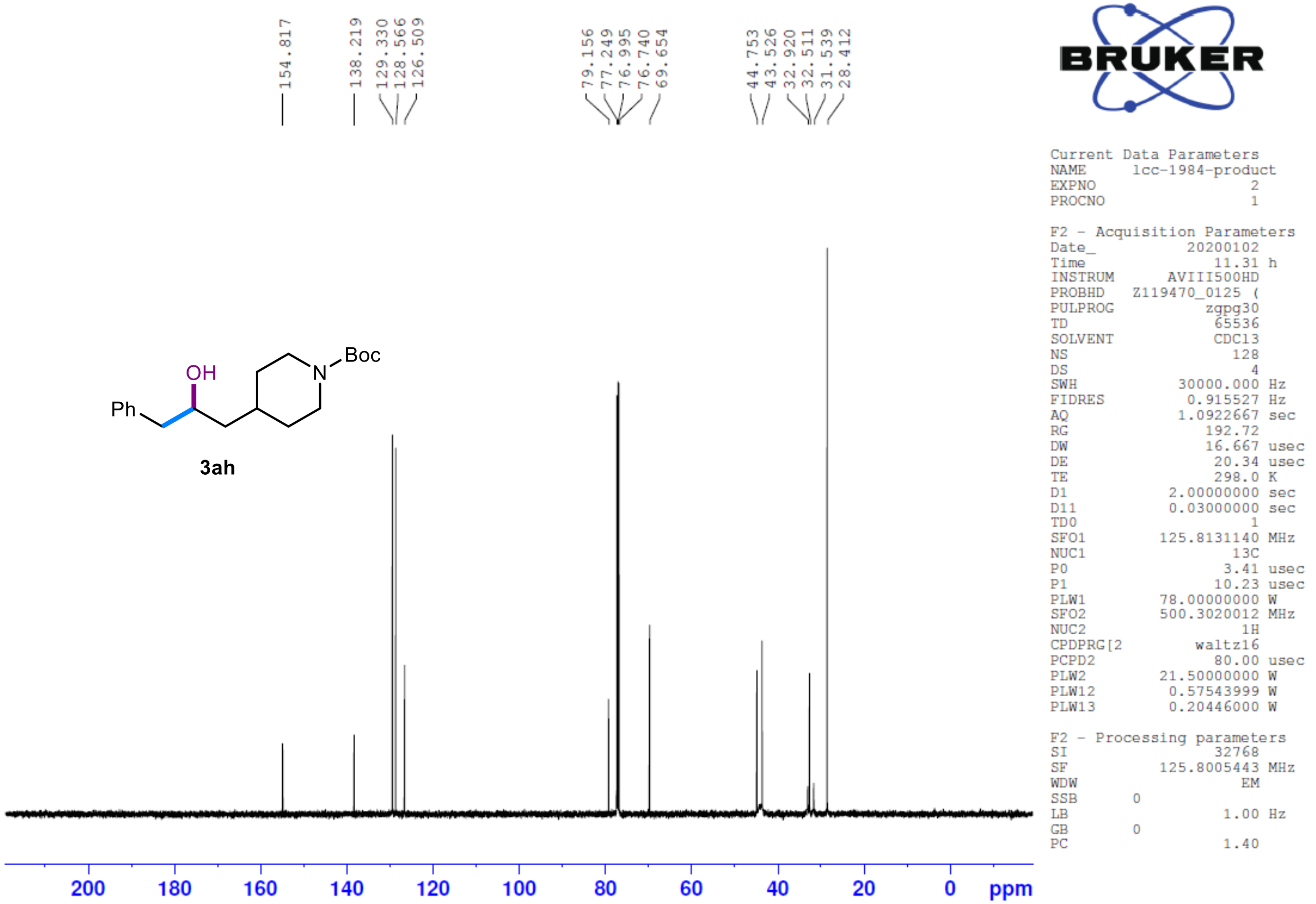
154.817
138.219
129.330
128.566
126.509
79.156
77.249
76.995
76.740
69.654
44.753
43.526
32.920
32.511
31.539
28.412
OH
Ph
N
Boc
3ah
BRUKER
Current Data Parameters
NAME lcc-1984-product
EXPNO 2
PROCNO 1
F2 - Acquisition Parameters
Date_ 20200102
Time 11.31 h
INSTRUM AVIII500HD
PROBHD Z119470_0125 (
PULPROG zgpg30
TD 65536
SOLVENT CDCl3
NS 128
DS 4
SWH 30000.000 Hz
FIDRES 0.915527 Hz
AQ 1.0922667 sec
RG 192.72
DW 16.667 usec
DE 20.34 usec
TE 298.0 K
D1 2.00000000 sec
D11 0.03000000 sec
TD0 1
SFO1 125.8131140 MHz
NUC1 13C
P0 3.41 usec
P1 10.23 usec
PLW1 78.00000000 W
SFO2 500.3020012 MHz
NUC2 1H
CPDPRG[2 waltz16
PCPD2 80.00 usec
PLW2 21.50000000 W
PLW12 0.57543999 W
PLW13 0.20446000 W
F2 - Processing parameters
SI 32768
SF 125.8005443 MHz
WDW EM
SSB 0
LB 1.00 Hz
GB 0
PC 1.40
200
180
160
140
120
100
80
60
40
20
0
ppm

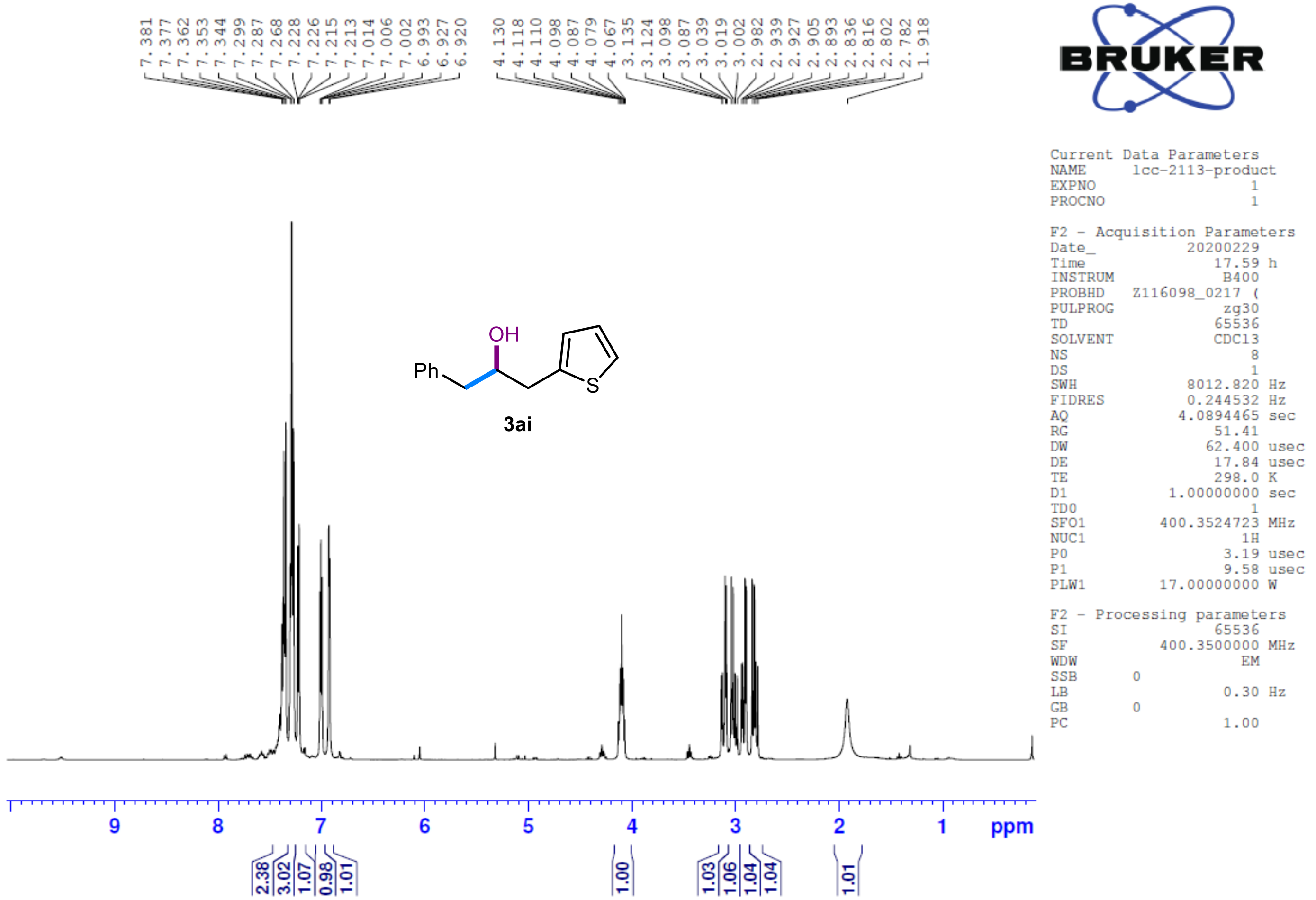
7.381 7.377 7.362 7.353 7.344 7.299 7.287 7.268 7.228 7.226 7.215 7.213 7.014 7.006 7.002 6.993 6.927 6.920
4.130 4.118 4.110 4.098 4.087 4.079 4.067 3.135 3.124 3.098 3.087 3.039 3.019 3.002 2.982 2.939 2.927 2.905 2.893 2.836 2.816 2.802 2.782 1.918
OH
Ph
S
3ai
BRUKER
Current Data Parameters
NAME lcc-2113-product
EXPNO 1
PROCNO 1
F2 - Acquisition Parameters
Date_ 20200229
Time 17.59 h
INSTRUM B400
PROBHD Z116098_0217 (
PULPROG zg30
TD 65536
SOLVENT CDCl3
NS 8
DS 1
SWH 8012.820 Hz
FIDRES 0.244532 Hz
AQ 4.0894465 sec
RG 51.41
DW 62.400 usec
DE 17.84 usec
TE 298.0 K
D1 1.00000000 sec
TD0 1
SFO1 400.3524723 MHz
NUC1 1H
P0 3.19 usec
P1 9.58 usec
PLW1 17.00000000 W
F2 - Processing parameters
SI 65536
SF 400.3500000 MHz
WDW EM
SSB 0
LB 0.30 Hz
GB 0
PC 1.00
9 8 7 6 5 4 3 2 1 ppm
2.38 3.02 1.07 0.98 1.01 1.00 1.03 1.06 1.04 1.04 1.01

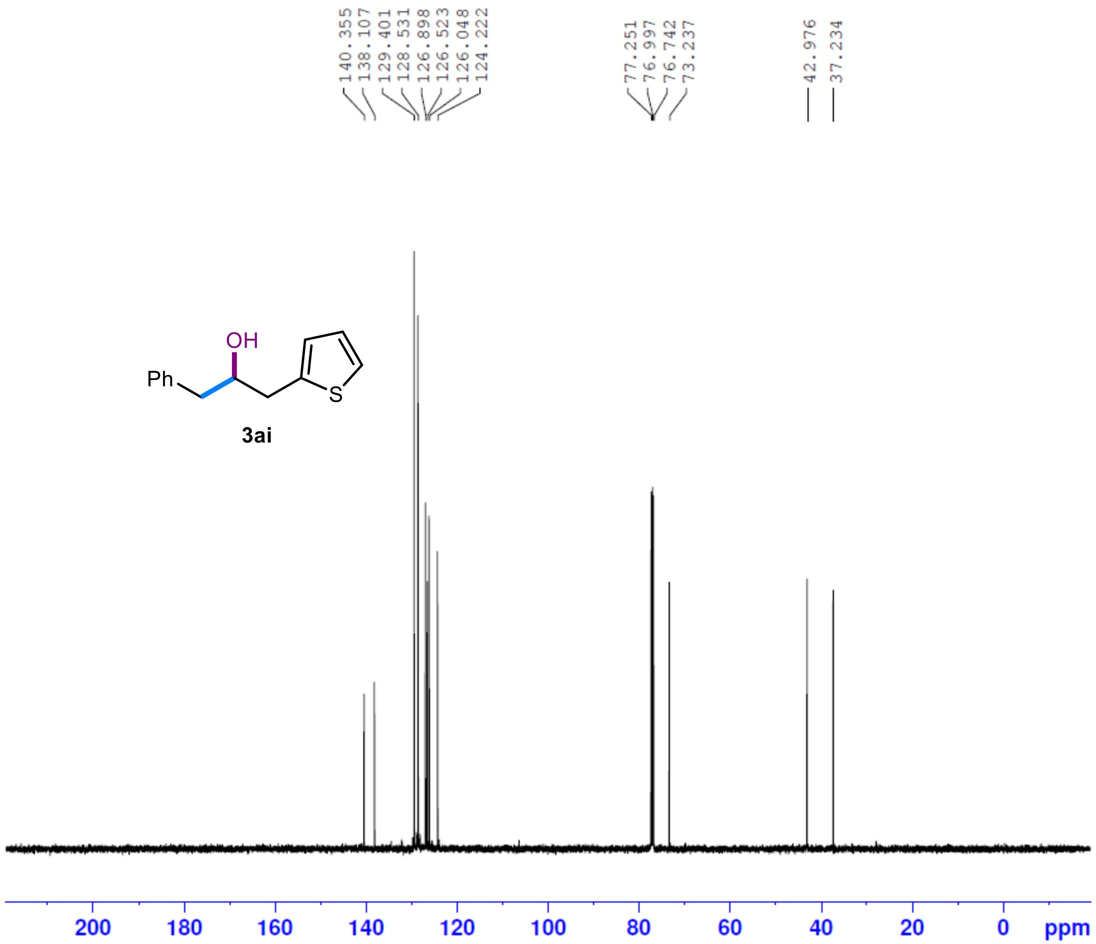


```
Current Data Parameters
NAME       lcc-2113-product
EXPNO                     2
PROCNO                    1

F2 - Acquisition Parameters
Date_              20200229
Time                  23.04 h
INSTRUM          AVIII500HD
PROBHD   Z119470_0125 (
PULPROG              zgpg30
TD                    65536
SOLVENT               CDCl3
NS                      128
DS                        4
SWH               30000.000 Hz
FIDRES             0.915527 Hz
AQ                1.0922667 sec
RG                   192.72
DW                   16.667 usec
DE                    20.34 usec
TE                    298.0 K
D1               2.00000000 sec
D11              0.03000000 sec
TD0                       1
SFO1            125.8131140 MHz
NUC1                    13C
P0                     3.41 usec
P1                    10.23 usec
PLW1            78.00000000 W
SFO2            500.3020012 MHz
NUC2                     1H
CPDPRG[2            waltz16
PCPD2                 80.00 usec
PLW2            21.50000000 W
PLW12            0.57543999 W
PLW13            0.20446000 W

F2 - Processing parameters
SI                    32768
SF              125.8005453 MHz
WDW                      EM
SSB          0
LB                     1.00 Hz
GB           0
PC                     1.40
```

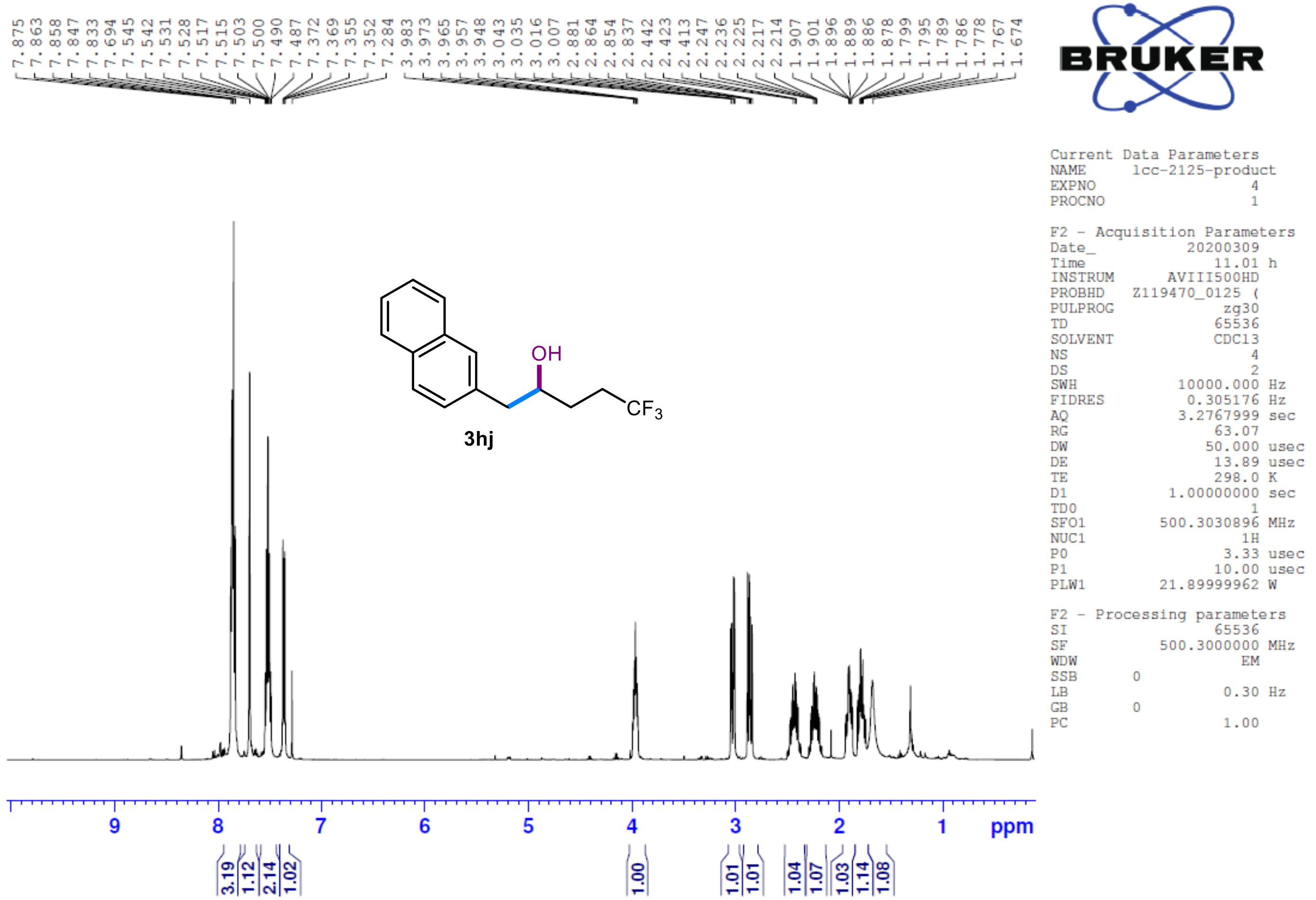
7.875 7.863 7.858 7.847 7.833 7.694 7.545 7.542 7.531 7.528 7.517 7.515 7.503 7.500 7.490 7.487 7.372 7.369 7.355 7.352 7.284 3.983 3.973 3.965 3.957 3.948 3.043 3.035 3.016 3.007 2.881 2.864 2.854 2.837 2.442 2.423 2.413 2.247 2.236 2.225 2.217 2.214 1.907 1.901 1.896 1.889 1.886 1.878 1.799 1.795 1.789 1.786 1.778 1.767 1.674
OH
CF3
3hj
BRUKER
Current Data Parameters
NAME lcc-2125-product
EXPNO 4
PROCNO 1
F2 - Acquisition Parameters
Date_ 20200309
Time 11.01 h
INSTRUM AVIII500HD
PROBHD Z119470_0125 (
PULPROG zg30
TD 65536
SOLVENT CDCl3
NS 4
DS 2
SWH 10000.000 Hz
FIDRES 0.305176 Hz
AQ 3.2767999 sec
RG 63.07
DW 50.000 usec
DE 13.89 usec
TE 298.0 K
D1 1.00000000 sec
TD0 1
SFO1 500.3030896 MHz
NUC1 1H
P0 3.33 usec
P1 10.00 usec
PLW1 21.89999962 W
F2 - Processing parameters
SI 65536
SF 500.3000000 MHz
WDW EM
SSB 0
LB 0.30 Hz
GB 0
PC 1.00
9 8 7 6 5 4 3 2 1 ppm
3.19 1.12 2.14 1.02 1.00 1.01 1.01 1.04 1.07 1.03 1.14 1.08

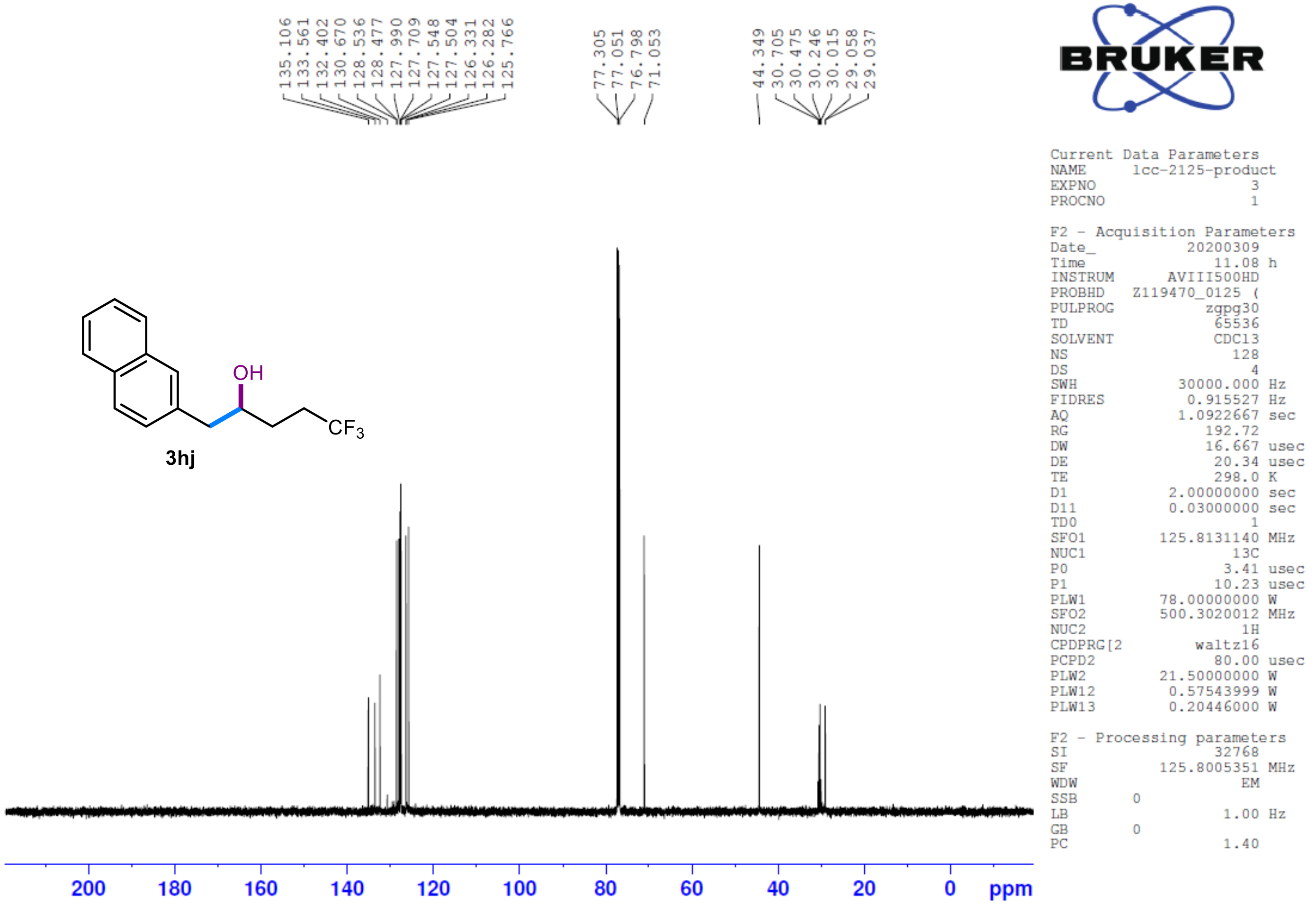
135.106
133.561
132.402
130.670
128.536
128.477
127.990
127.709
127.548
127.504
126.331
126.282
125.766
77.305
77.051
76.798
71.053
44.349
30.705
30.475
30.246
30.015
29.058
29.037
OH
CF3
3hj
BRUKER
Current Data Parameters
NAME lcc-2125-product
EXPNO 3
PROCNO 1
F2 - Acquisition Parameters
Date_ 20200309
Time 11.08 h
INSTRUM AVIII500HD
PROBHD Z119470_0125 (
PULPROG zgpg30
TD 65536
SOLVENT CDCl3
NS 128
DS 4
SWH 30000.000 Hz
FIDRES 0.915527 Hz
AQ 1.0922667 sec
RG 192.72
DW 16.667 usec
DE 20.34 usec
TE 298.0 K
D1 2.00000000 sec
D11 0.03000000 sec
TD0 1
SFO1 125.8131140 MHz
NUC1 13C
P0 3.41 usec
P1 10.23 usec
PLW1 78.00000000 W
SFO2 500.3020012 MHz
NUC2 1H
CPDPRG[2 waltz16
PCPD2 80.00 usec
PLW2 21.50000000 W
PLW12 0.57543999 W
PLW13 0.20446000 W
F2 - Processing parameters
SI 32768
SF 125.8005351 MHz
WDW EM
SSB 0
LB 1.00 Hz
GB 0
PC 1.40
200
180
160
140
120
100
80
60
40
20
0
ppm

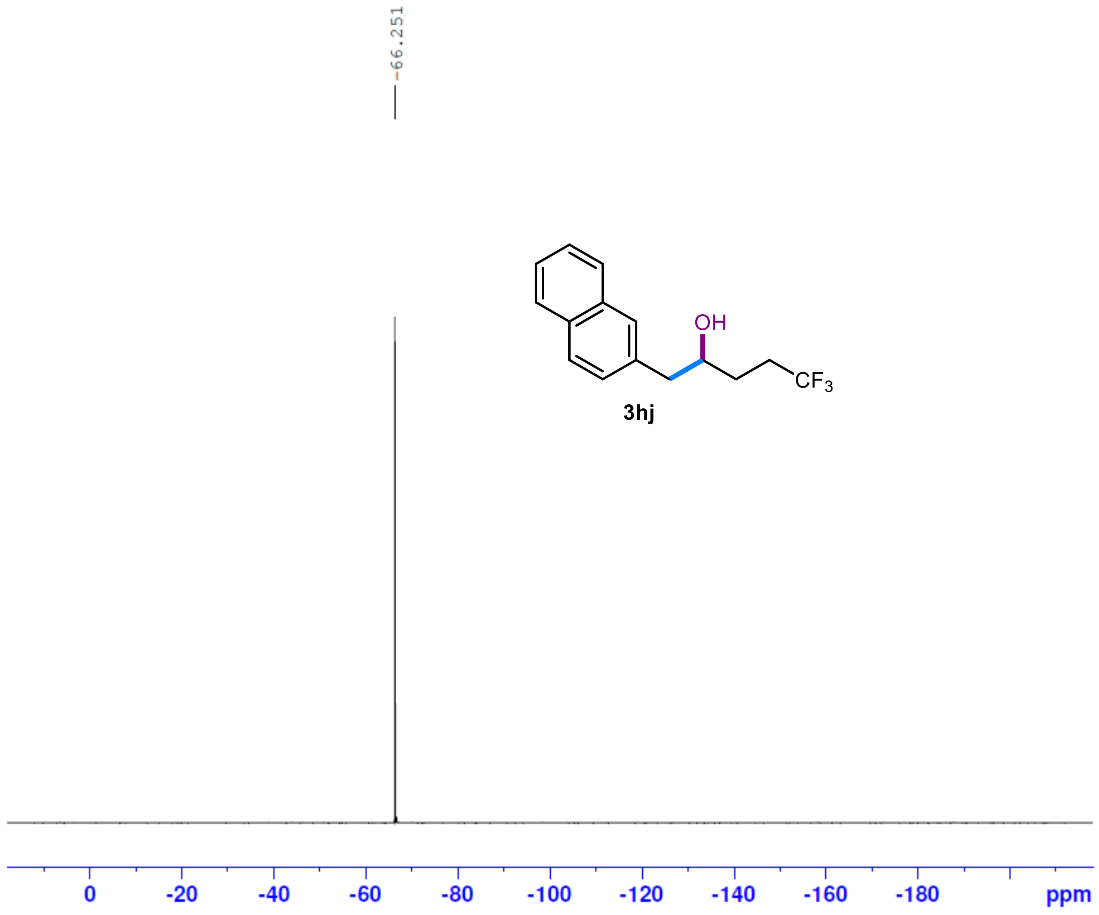


```
Current Data Parameters
NAME      lcc-2017-product
EXPNO                    3
PROCNO                   1

F2 - Acquisition Parameters
Date_             20200119
Time                 18.55 h
INSTRUM         AVIII500HD
PROBHD    Z119470_0125 (
PULPROG   zgbsfhigqn.baseopt
TD                  131072
SOLVENT              CDCl3
NS                      16
DS                       4
SWH            111111.109 Hz
FIDRES           1.695421 Hz
AQ              0.5898240 sec
RG                  192.72
DW                   4.500 usec
DE                   35.33 usec
TE                   298.0 K
D1              1.00000000 sec
D11             0.03000000 sec
D12             0.00002000 sec
TD0                      1
SFO1           470.7052618 MHz
NUC1                   19F
P1                   15.00 usec
P2                   30.00 usec
PLW1           39.50000000 W
SFO2           500.3025015 MHz
NUC2                    1H
CPDPRG[2           waltz16
PCPD2                80.00 usec
PLW2           21.50000000 W
PLW12           0.57543999 W

F2 - Processing parameters
SI                   65536
SF             470.7523370 MHz
WDW                     EM
SSB       0
LB                    0.30 Hz
GB        0
PC                    1.00
```

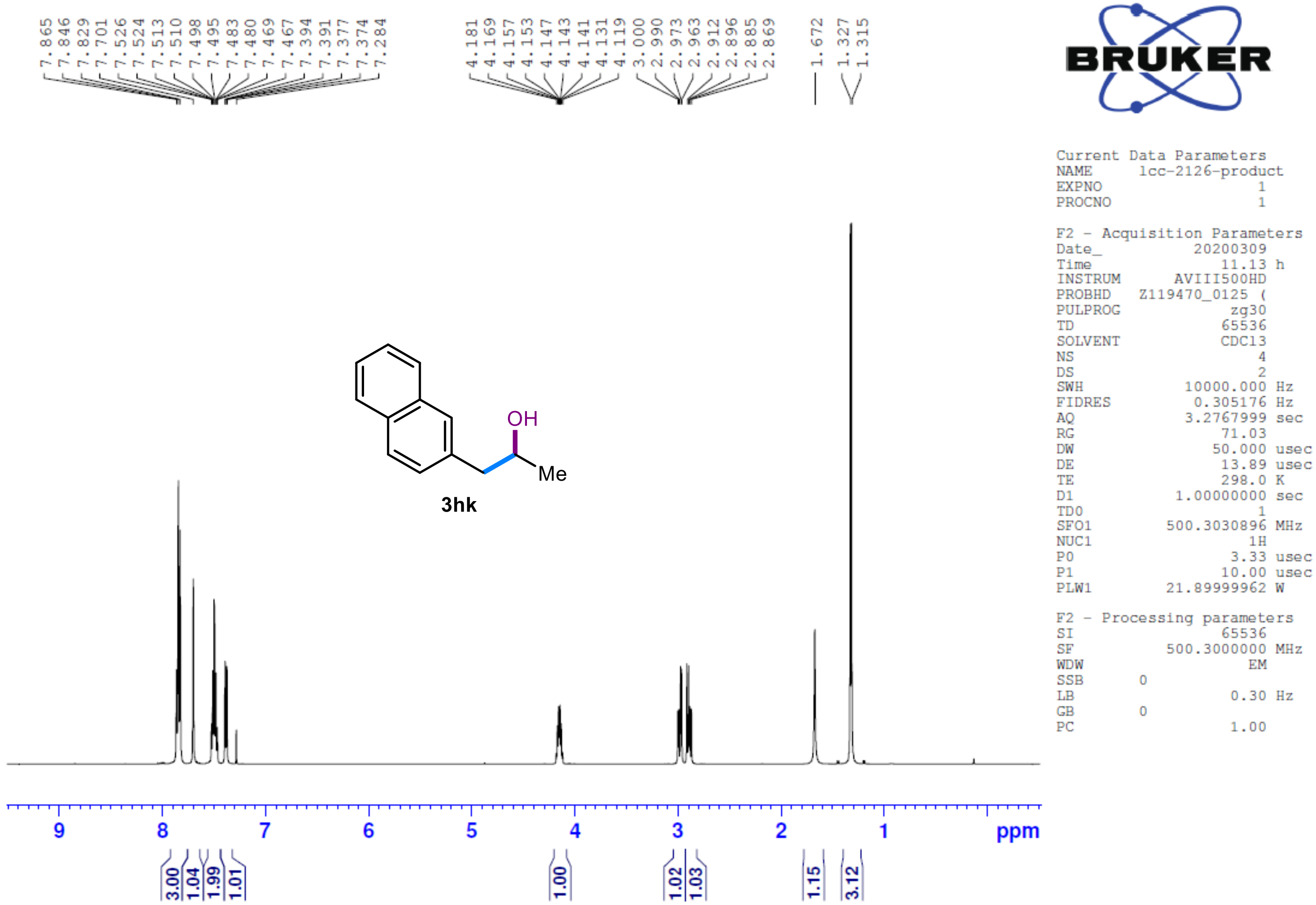
7.865 7.846 7.829 7.701 7.526 7.524 7.513 7.510 7.498 7.495 7.483 7.480 7.469 7.467 7.394 7.391 7.377 7.374 7.284
4.181 4.169 4.157 4.153 4.147 4.143 4.141 4.131 4.119 3.000 2.990 2.973 2.963 2.912 2.896 2.885 2.869
1.672 1.327 1.315
OH
Me
3hk
BRUKER
Current Data Parameters
NAME lcc-2126-product
EXPNO 1
PROCNO 1
F2 - Acquisition Parameters
Date_ 20200309
Time 11.13 h
INSTRUM AVIII500HD
PROBHD Z119470_0125 (
PULPROG zg30
TD 65536
SOLVENT CDCl3
NS 4
DS 2
SWH 10000.000 Hz
FIDRES 0.305176 Hz
AQ 3.2767999 sec
RG 71.03
DW 50.000 usec
DE 13.89 usec
TE 298.0 K
D1 1.00000000 sec
TD0 1
SFO1 500.3030896 MHz
NUC1 1H
P0 3.33 usec
P1 10.00 usec
PLW1 21.89999962 W
F2 - Processing parameters
SI 65536
SF 500.3000000 MHz
WDW EM
SSB 0
LB 0.30 Hz
GB 0
PC 1.00
9 8 7 6 5 4 3 2 1 ppm
3.00 1.04 1.99 1.01 1.00 1.02 1.03 1.15 3.12

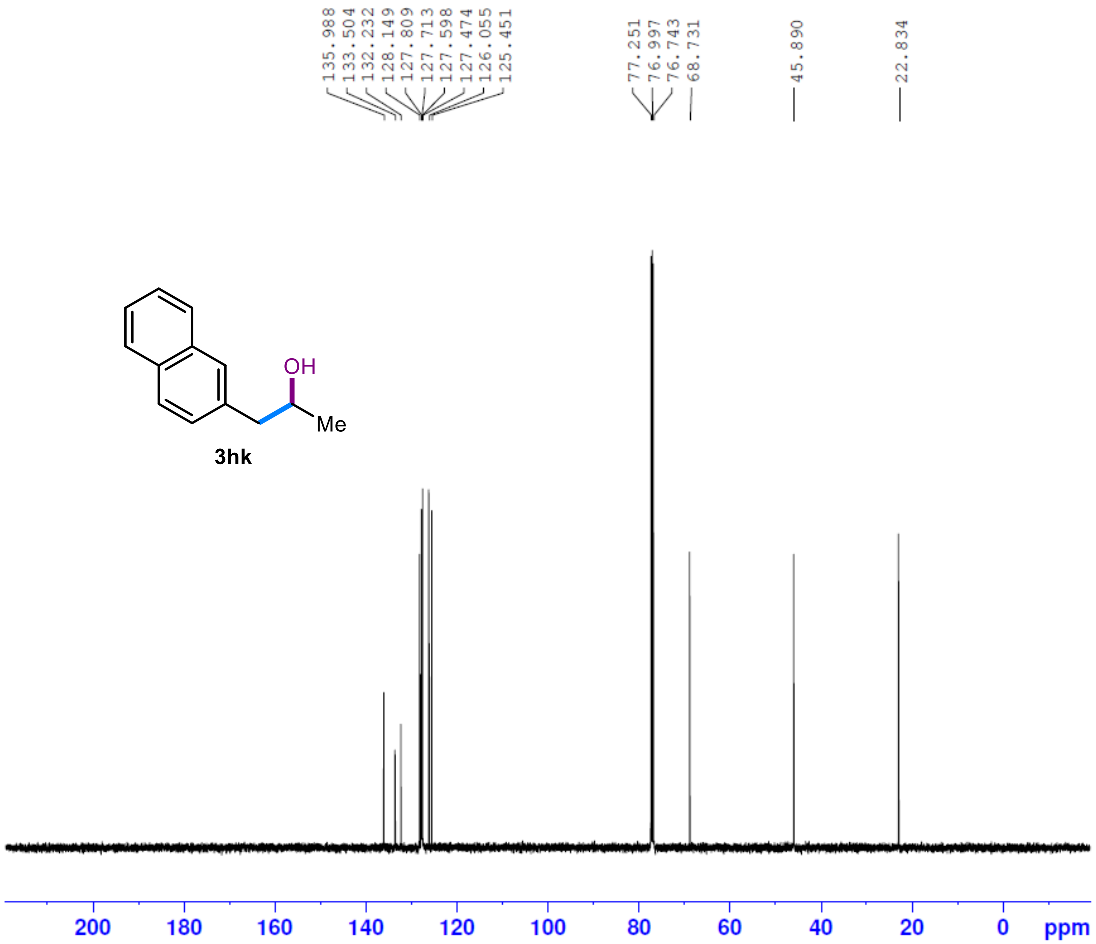

Current Data Parameters
NAME lcc-2126-product
EXPNO 2
PROCNO 1

F2 - Acquisition Parameters
Date_ 20200309
Time 11.20 h
INSTRUM AVIII500HD
PROBHD Z119470_0125 (
PULPROG zgpg30
TD 65536
SOLVENT CDCl3
NS 128
DS 4
SWH 30000.000 Hz
FIDRES 0.915527 Hz
AQ 1.0922667 sec
RG 192.72
DW 16.667 usec
DE 20.34 usec
TE 298.0 K
D1 2.00000000 sec
D11 0.03000000 sec
TD0 1
SFO1 125.8131140 MHz
NUC1 13C
P0 3.41 usec
P1 10.23 usec
PLW1 78.00000000 W
SFO2 500.3020012 MHz
NUC2 1H
CPDPRG[2 waltz16
PCPD2 80.00 usec
PLW2 21.50000000 W
PLW12 0.57543999 W
PLW13 0.20446000 W

F2 - Processing parameters
SI 32768
SF 125.8005443 MHz
WDW EM
SSB 0
LB 1.00 Hz
GB 0
PC 1.40

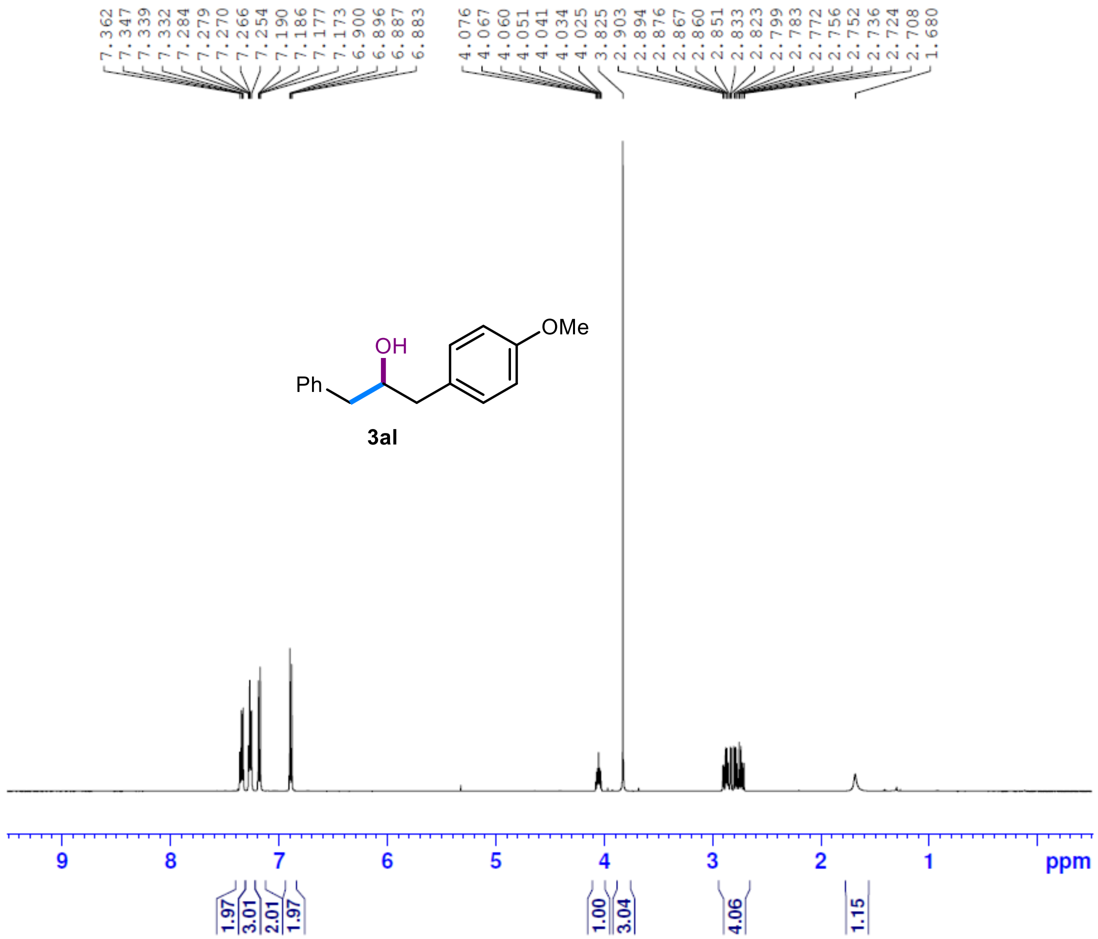

Current Data Parameters
NAME lcc-2139-product
EXPNO 1
PROCNO 1

F2 - Acquisition Parameters
Date_ 20200313
Time 8.38 h
INSTRUM AVIII500HD
PROBHD Z119470_0125 (
PULPROG zg30
TD 65536
SOLVENT CDCl3
NS 4
DS 2
SWH 10000.000 Hz
FIDRES 0.305176 Hz
AQ 3.2767999 sec
RG 71.03
DW 50.000 usec
DE 13.89 usec
TE 298.0 K
D1 1.00000000 sec
TD0 1
SFO1 500.3030896 MHz
NUC1 1H
P0 3.33 usec
P1 10.00 usec
PLW1 21.89999962 W

F2 - Processing parameters
SI 65536
SF 500.3000000 MHz
WDW EM
SSB 0
LB 0.30 Hz
GB 0
PC 1.00

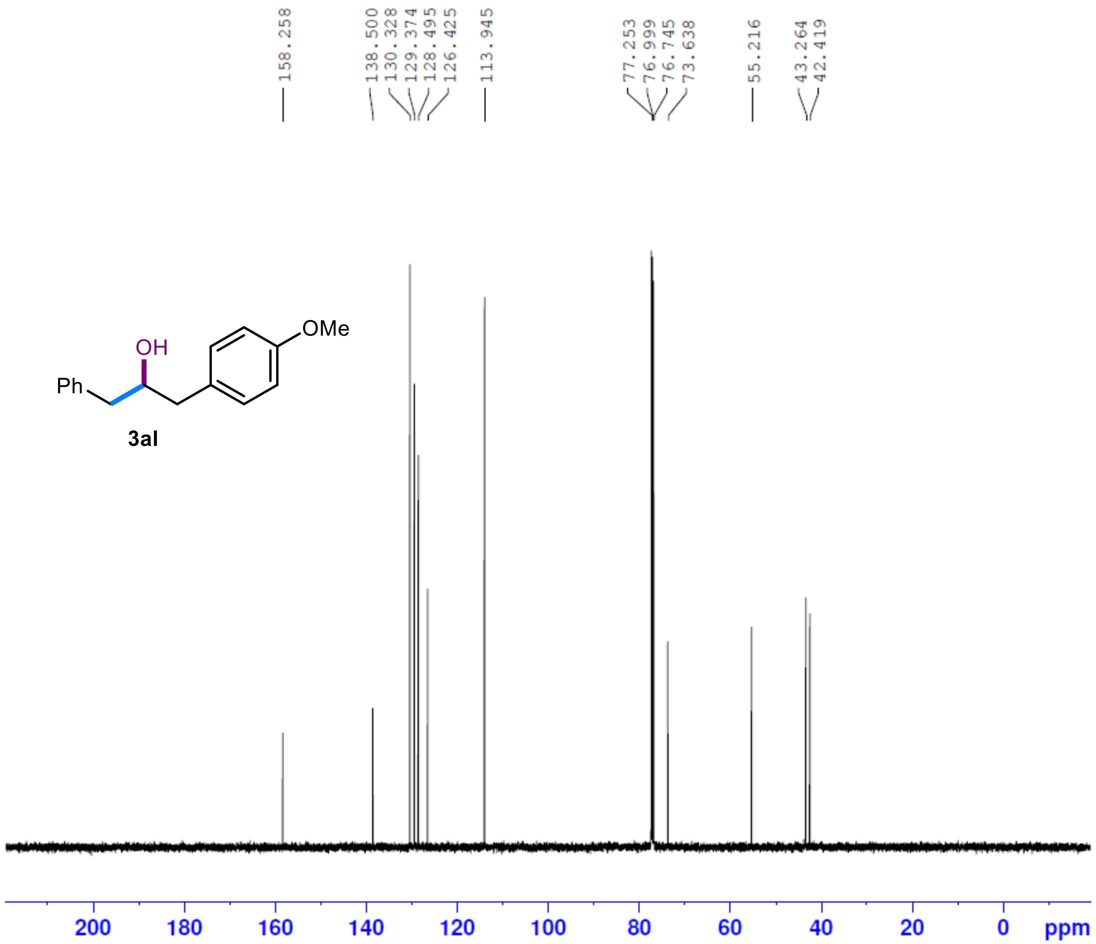

3al

```
Current Data Parameters
NAME       lcc-2139-product
EXPNO                     2
PROCNO                    1

F2 - Acquisition Parameters
Date_             20200313
Time                  8.45 h
INSTRUM         AVIII500HD
PROBHD    Z119470_0125 (
PULPROG            zgpg30
TD                  65536
SOLVENT             CDCl3
NS                    128
DS                      4
SWH             30000.000 Hz
FIDRES           0.915527 Hz
AQ              1.0922667 sec
RG                 192.72
DW                 16.667 usec
DE                  20.34 usec
TE                  298.0 K
D1             2.00000000 sec
D11            0.03000000 sec
TD0                     1
SFO1          125.8131140 MHz
NUC1                  13C
P0                   3.41 usec
P1                  10.23 usec
PLW1          78.00000000 W
SFO2          500.3020012 MHz
NUC2                   1H
CPDPRG[2          waltz16
PCPD2               80.00 usec
PLW2          21.50000000 W
PLW12          0.57543999 W
PLW13          0.20446000 W

F2 - Processing parameters
SI                  32768
SF            125.8005425 MHz
WDW                    EM
SSB      0
LB                   1.00 Hz
GB       0
PC                   1.40
```

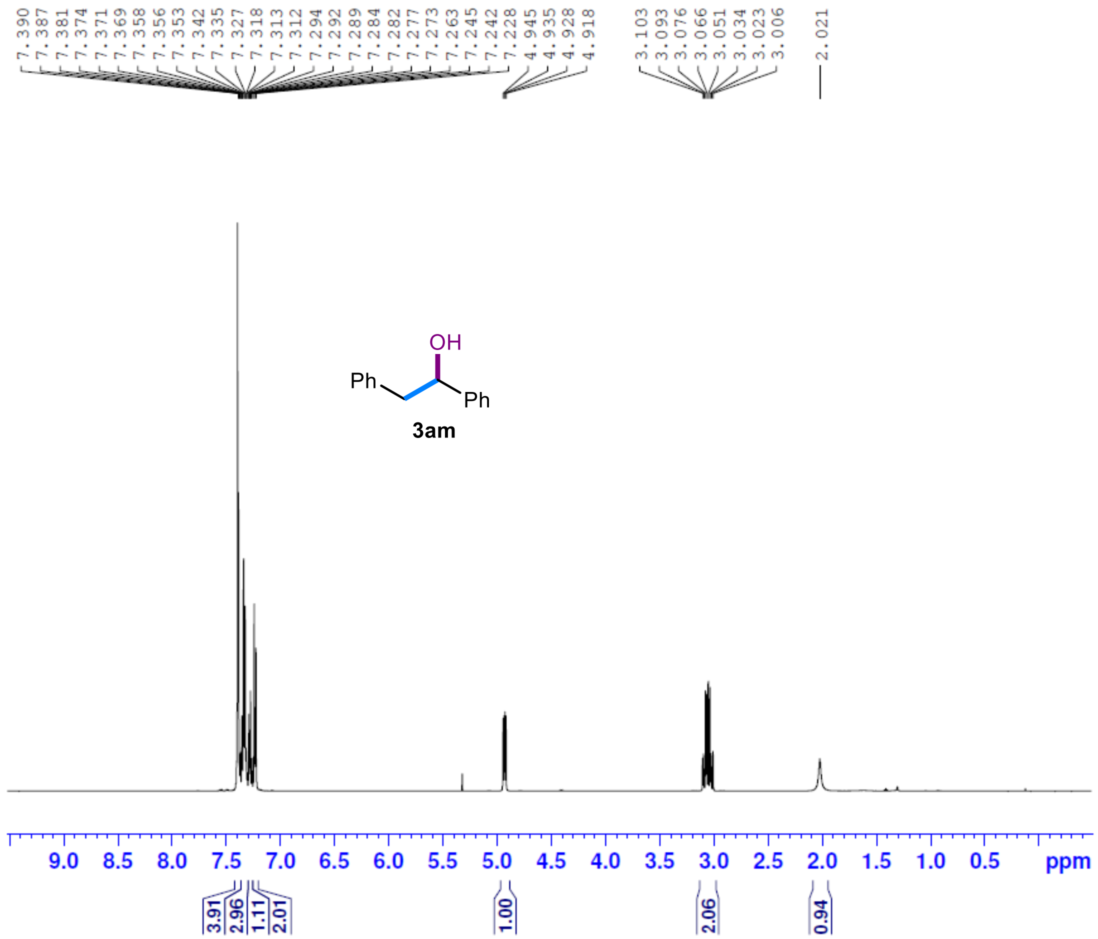


```
Current Data Parameters
NAME      lcc-2114-product
EXPNO                    1
PROCNO                   1

F2 - Acquisition Parameters
Date_             20200229
Time                 23.08 h
INSTRUM         AVIII500HD
PROBHD    Z119470_0125 (
PULPROG               zg30
TD                   65536
SOLVENT              CDCl3
NS                       4
DS                       2
SWH              10000.000 Hz
FIDRES            0.305176 Hz
AQ               3.2767999 sec
RG                   77.88
DW                  50.000 usec
DE                   13.89 usec
TE                   298.0 K
D1              1.00000000 sec
TD0                      1
SFO1           500.3030896 MHz
NUC1                    1H
P0                    3.33 usec
P1                   10.00 usec
PLW1           21.89999962 W

F2 - Processing parameters
SI                   65536
SF             500.3000000 MHz
WDW                     EM
SSB       0
LB                    0.30 Hz
GB        0
PC                    1.00
```

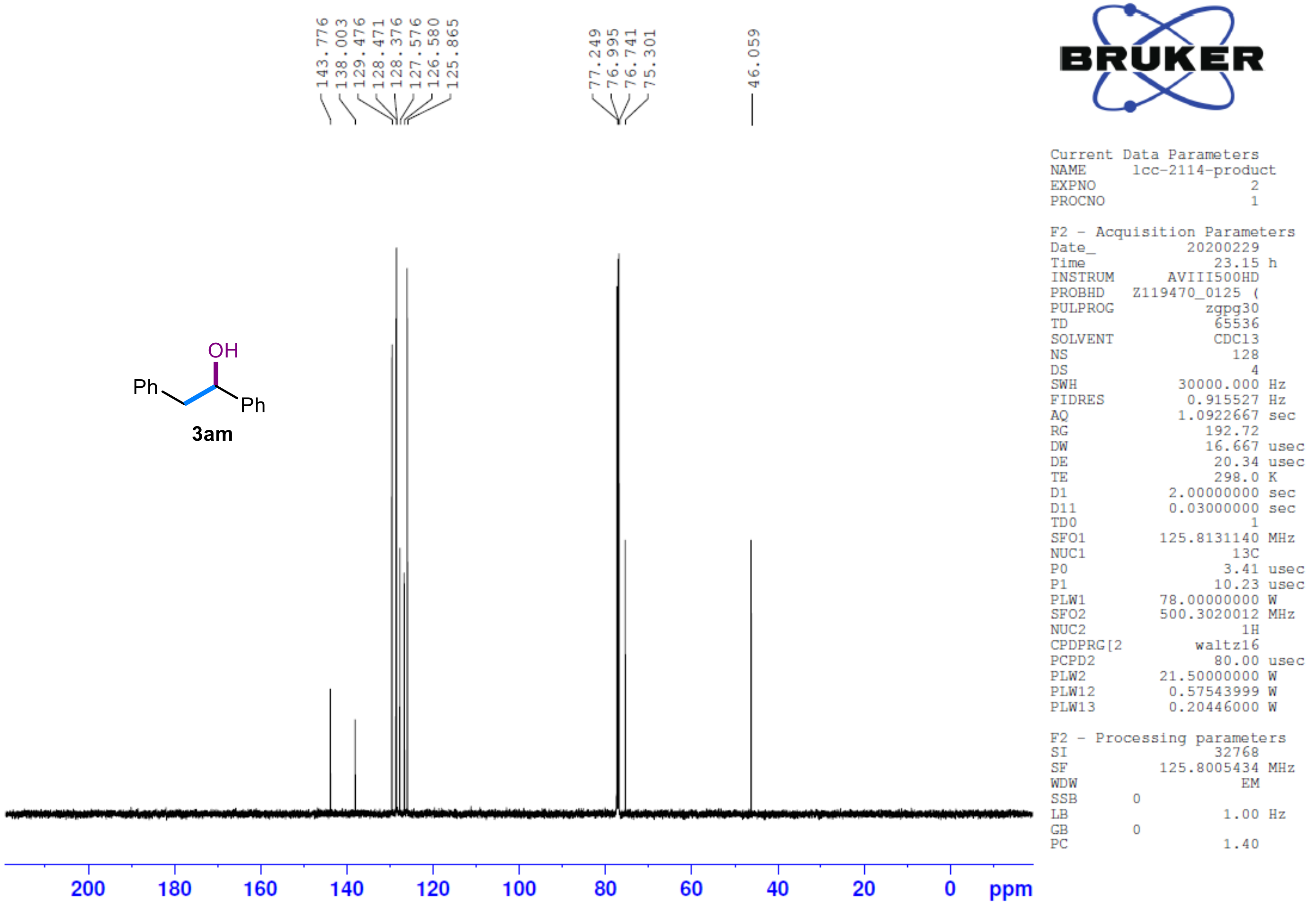
143.776
138.003
129.476
128.471
128.376
127.576
126.580
125.865
77.249
76.995
76.741
75.301
46.059
OH
Ph
Ph
3am
200 180 160 140 120 100 80 60 40 20 0 ppm
Current Data Parameters
NAME lcc-2114-product
EXPNO 2
PROCNO 1
F2 - Acquisition Parameters
Date_ 20200229
Time 23.15 h
INSTRUM AVIII500HD
PROBHD Z119470_0125 (
PULPROG zgpg30
TD 65536
SOLVENT CDCl3
NS 128
DS 4
SWH 30000.000 Hz
FIDRES 0.915527 Hz
AQ 1.0922667 sec
RG 192.72
DW 16.667 usec
DE 20.34 usec
TE 298.0 K
D1 2.00000000 sec
D11 0.03000000 sec
TD0 1
SFO1 125.8131140 MHz
NUC1 13C
P0 3.41 usec
P1 10.23 usec
PLW1 78.00000000 W
SFO2 500.3020012 MHz
NUC2 1H
CPDPRG[2 waltz16
PCPD2 80.00 usec
PLW2 21.50000000 W
PLW12 0.57543999 W
PLW13 0.20446000 W
F2 - Processing parameters
SI 32768
SF 125.8005434 MHz
WDW EM
SSB 0
LB 1.00 Hz
GB 0
PC 1.40

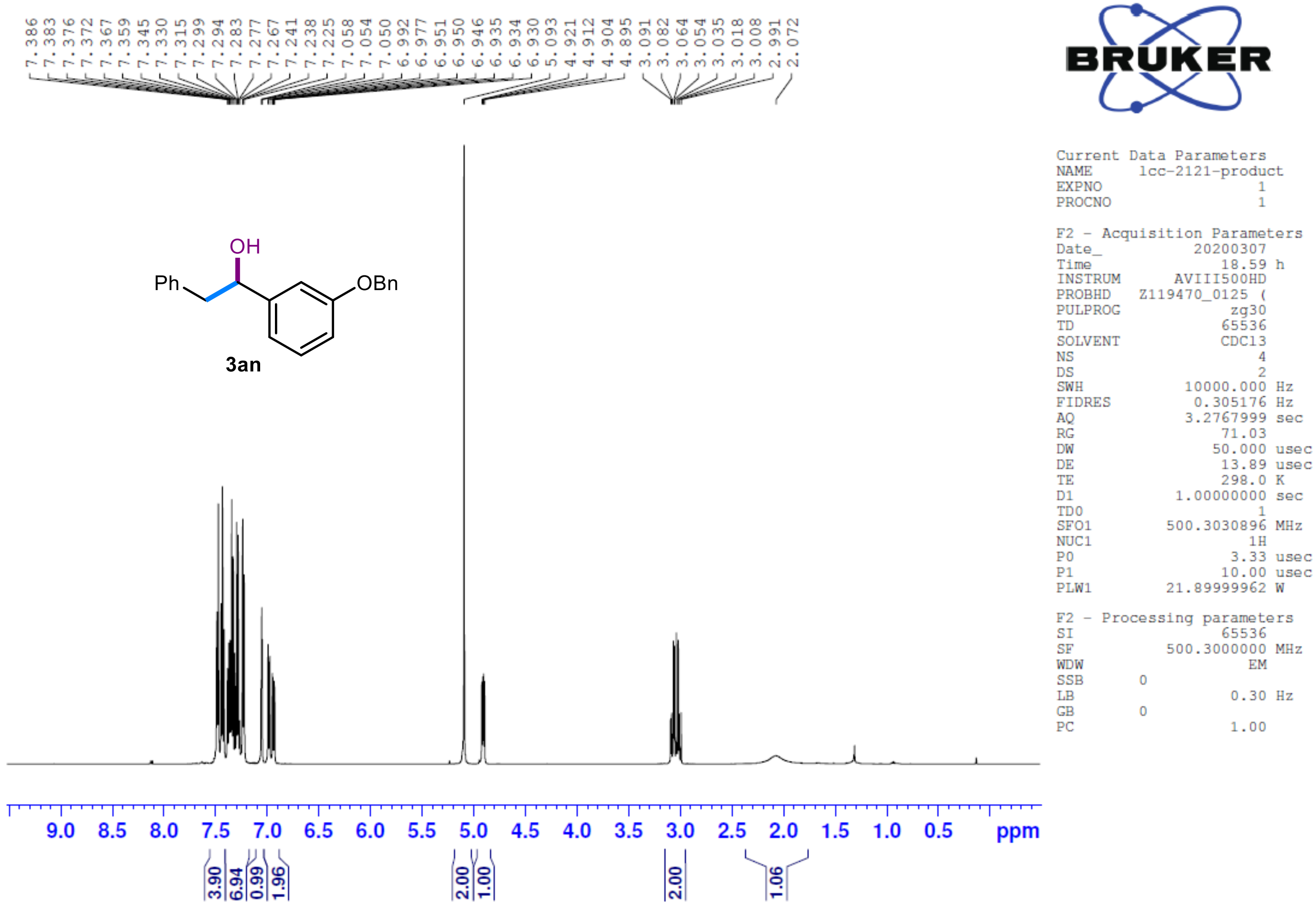
7.386 7.383 7.376 7.372 7.367 7.359 7.345 7.330 7.315 7.299 7.294 7.283 7.277 7.267 7.241 7.238 7.225 7.058 7.054 7.050 6.992 6.977 6.951 6.950 6.946 6.935 6.934 6.930 5.093 4.921 4.912 4.904 4.895 3.091 3.082 3.064 3.054 3.035 3.018 3.008 2.991 2.072
OH
Ph
OBn
3an
Current Data Parameters
NAME lcc-2121-product
EXPNO 1
PROCNO 1
F2 - Acquisition Parameters
Date_ 20200307
Time 18.59 h
INSTRUM AVIII500HD
PROBHD Z119470_0125 (
PULPROG zg30
TD 65536
SOLVENT CDCl3
NS 4
DS 2
SWH 10000.000 Hz
FIDRES 0.305176 Hz
AQ 3.2767999 sec
RG 71.03
DW 50.000 usec
DE 13.89 usec
TE 298.0 K
D1 1.00000000 sec
TD0 1
SFO1 500.3030896 MHz
NUC1 1H
P0 3.33 usec
P1 10.00 usec
PLW1 21.89999962 W
F2 - Processing parameters
SI 65536
SF 500.3000000 MHz
WDW EM
SSB 0
LB 0.30 Hz
GB 0
PC 1.00
9.0 8.5 8.0 7.5 7.0 6.5 6.0 5.5 5.0 4.5 4.0 3.5 3.0 2.5 2.0 1.5 1.0 0.5 ppm
3.90 6.94 0.99 1.96 2.00 1.00 2.00 1.06

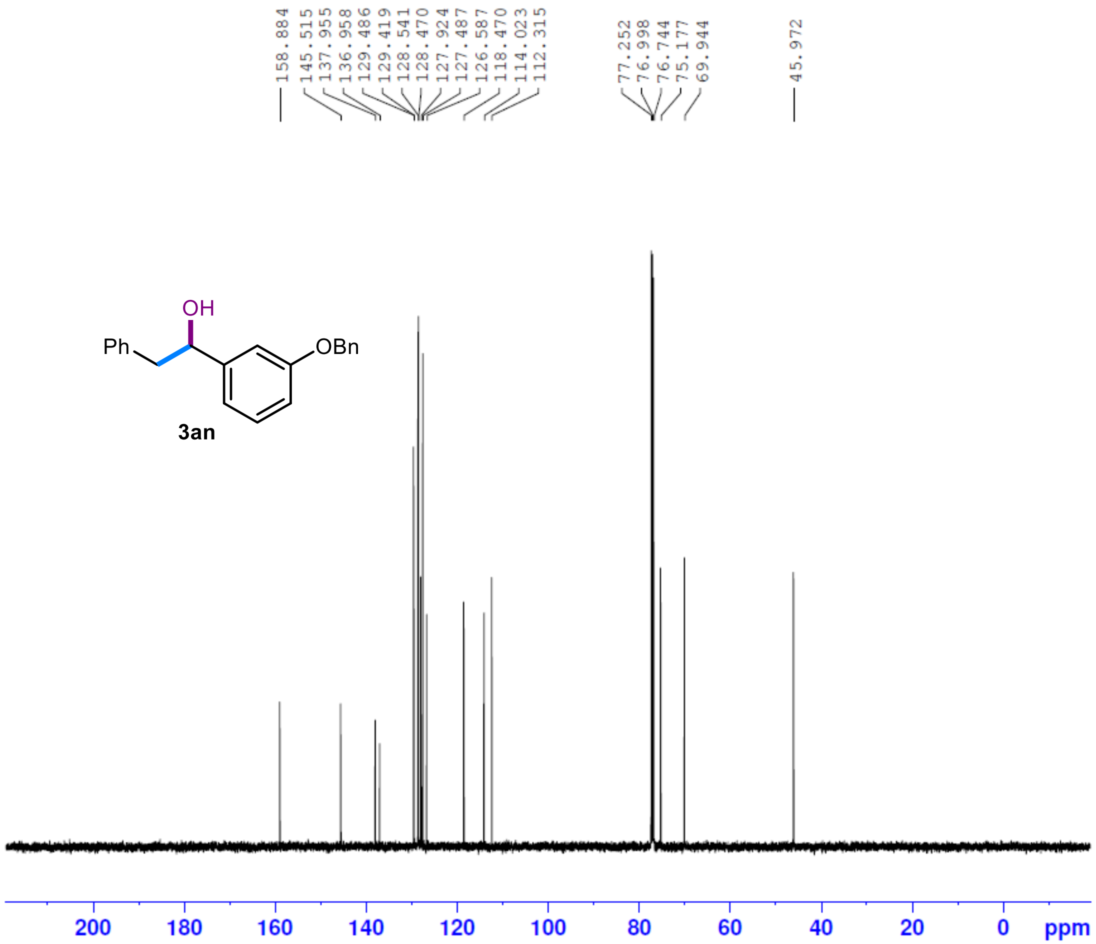

Current Data Parameters
NAME lcc-2121-product
EXPNO 2
PROCNO 1

F2 - Acquisition Parameters
Date_ 20200307
Time 19.06 h
INSTRUM AVIII500HD
PROBHD Z119470_0125 (
PULPROG zgpg30
TD 65536
SOLVENT CDCl3
NS 128
DS 4
SWH 30000.000 Hz
FIDRES 0.915527 Hz
AQ 1.0922667 sec
RG 192.72
DW 16.667 usec
DE 20.34 usec
TE 298.0 K
D1 2.00000000 sec
D11 0.03000000 sec
TD0 1
SFO1 125.8131140 MHz
NUC1 13C
P0 3.41 usec
P1 10.23 usec
PLW1 78.00000000 W
SFO2 500.3020012 MHz
NUC2 1H
CPDPRG[2 waltz16
PCPD2 80.00 usec
PLW2 21.50000000 W
PLW12 0.57543999 W
PLW13 0.20446000 W

F2 - Processing parameters
SI 32768
SF 125.8005444 MHz
WDW EM
SSB 0
LB 1.00 Hz
GB 0
PC 1.40

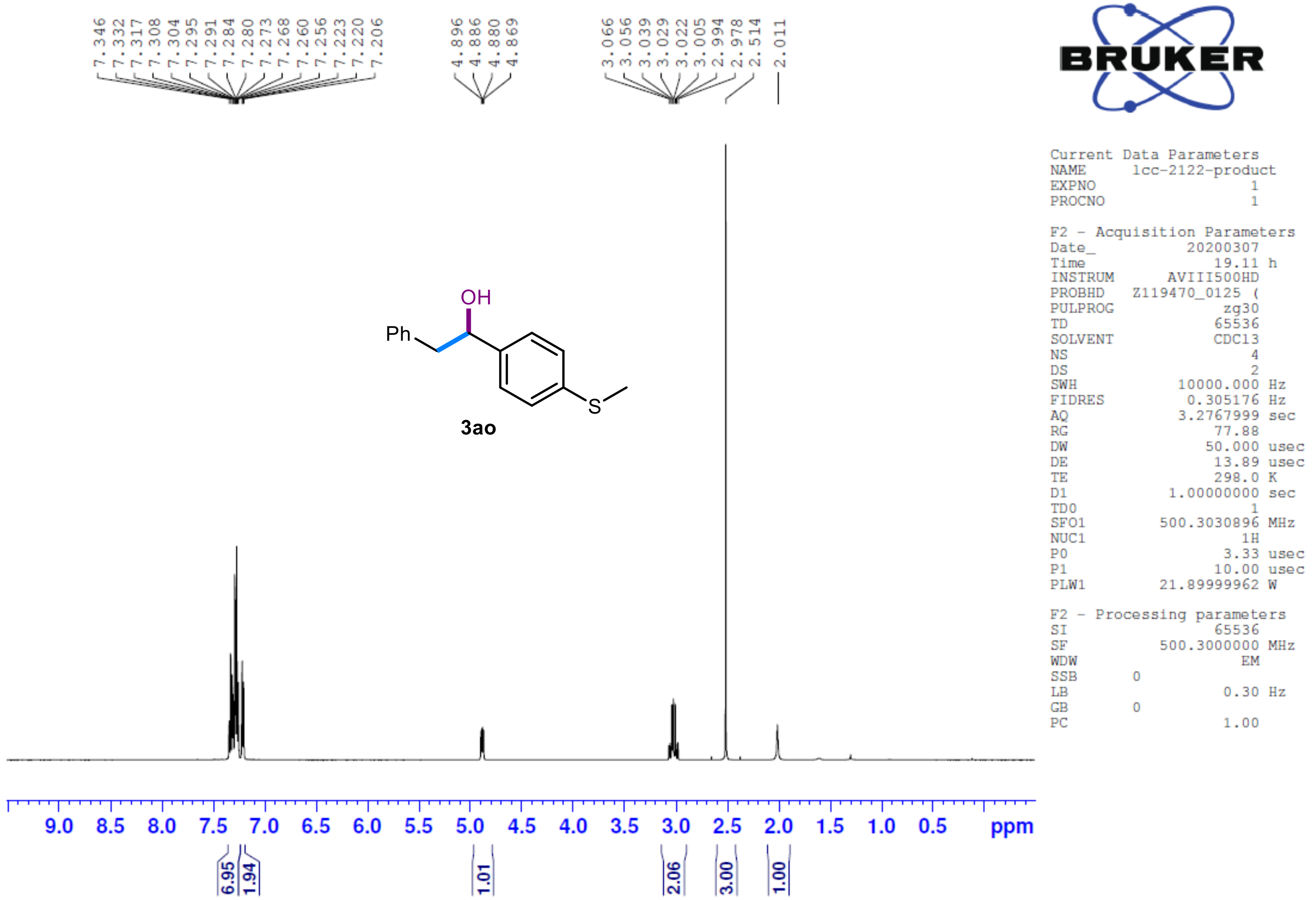
7.346
7.332
7.317
7.308
7.304
7.295
7.291
7.284
7.280
7.273
7.268
7.260
7.256
7.223
7.220
7.206
4.896
4.886
4.880
4.869
3.066
3.056
3.039
3.029
3.022
3.005
2.994
2.978
2.514
2.011
BRUKER
Current Data Parameters
NAME lcc-2122-product
EXPNO 1
PROCNO 1
F2 - Acquisition Parameters
Date_ 20200307
Time 19.11 h
INSTRUM AVIII500HD
PROBHD Z119470_0125 (
PULPROG zg30
TD 65536
SOLVENT CDCl3
NS 4
DS 2
SWH 10000.000 Hz
FIDRES 0.305176 Hz
AQ 3.2767999 sec
RG 77.88
DW 50.000 usec
DE 13.89 usec
TE 298.0 K
D1 1.00000000 sec
TD0 1
SFO1 500.3030896 MHz
NUC1 1H
P0 3.33 usec
P1 10.00 usec
PLW1 21.89999962 W
F2 - Processing parameters
SI 65536
SF 500.3000000 MHz
WDW EM
SSB 0
LB 0.30 Hz
GB 0
PC 1.00
OH
Ph
S
3ao
9.0 8.5 8.0 7.5 7.0 6.5 6.0 5.5 5.0 4.5 4.0 3.5 3.0 2.5 2.0 1.5 1.0 0.5 ppm
6.95
1.94
1.01
2.06
3.00
1.00

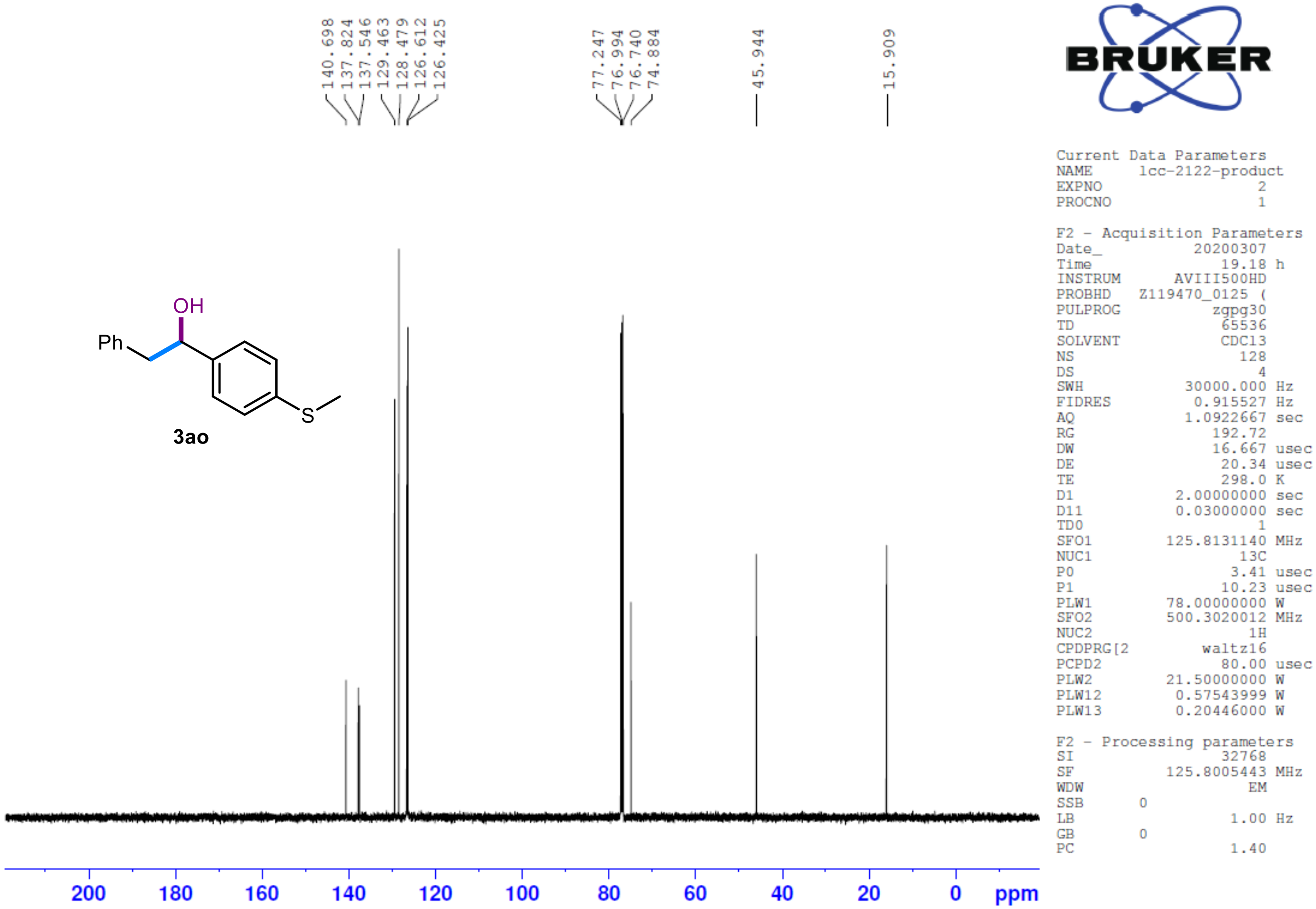
OH
Ph
S
3ao
140.698
137.824
137.546
129.463
128.479
126.612
126.425
77.247
76.994
76.740
74.884
45.944
15.909
200 180 160 140 120 100 80 60 40 20 0 ppm
BRUKER
Current Data Parameters
NAME lcc-2122-product
EXPNO 2
PROCNO 1
F2 - Acquisition Parameters
Date_ 20200307
Time 19.18 h
INSTRUM AVIII500HD
PROBHD Z119470_0125 (
PULPROG zgpg30
TD 65536
SOLVENT CDCl3
NS 128
DS 4
SWH 30000.000 Hz
FIDRES 0.915527 Hz
AQ 1.0922667 sec
RG 192.72
DW 16.667 usec
DE 20.34 usec
TE 298.0 K
D1 2.00000000 sec
D11 0.03000000 sec
TD0 1
SFO1 125.8131140 MHz
NUC1 13C
P0 3.41 usec
P1 10.23 usec
PLW1 78.00000000 W
SFO2 500.3020012 MHz
NUC2 1H
CPDPRG[2 waltz16
PCPD2 80.00 usec
PLW2 21.50000000 W
PLW12 0.57543999 W
PLW13 0.20446000 W
F2 - Processing parameters
SI 32768
SF 125.8005443 MHz
WDW EM
SSB 0
LB 1.00 Hz
GB 0
PC 1.40

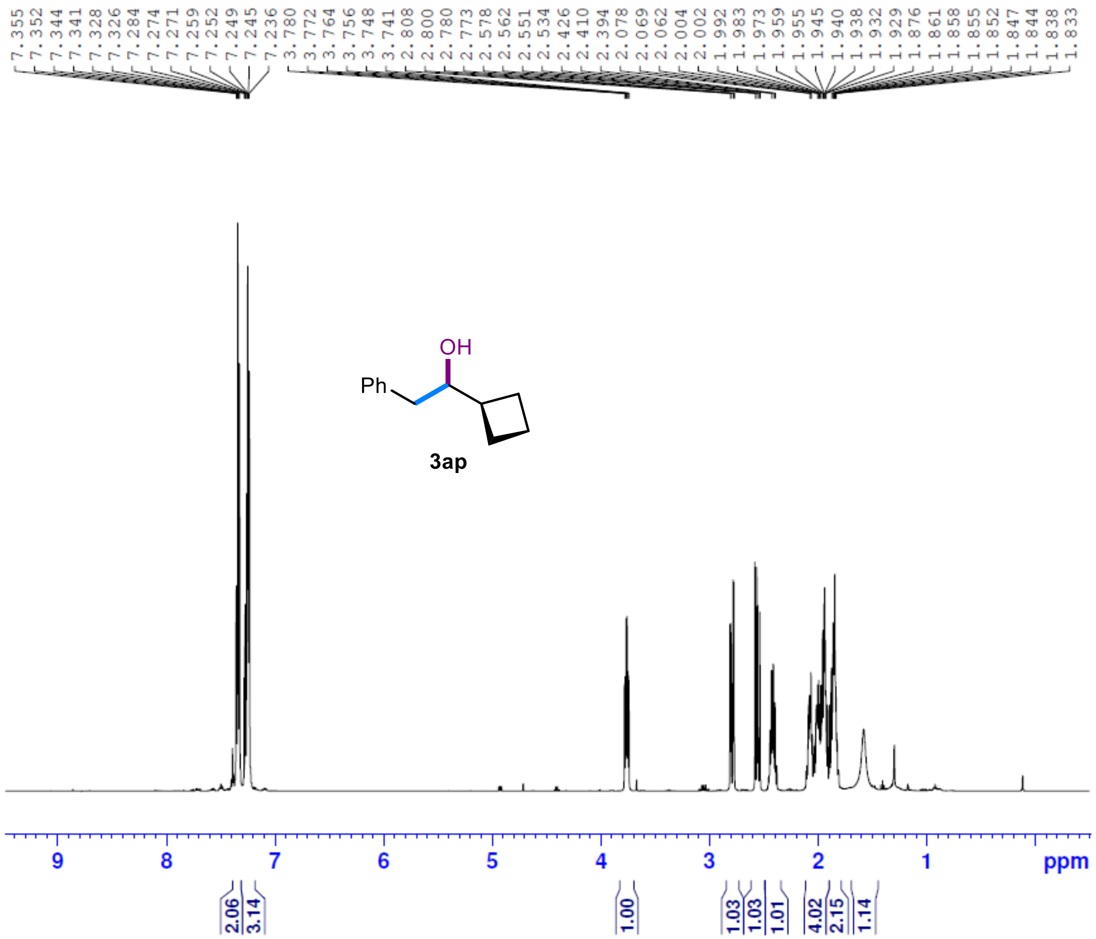


```
Current Data Parameters
NAME      lcc-2120-product
EXPNO                    1
PROCNO                   1

F2 - Acquisition Parameters
Date_           20200307
Time               18.35 h
INSTRUM       AVIII500HD
PROBHD   Z119470_0125 (
PULPROG             zg30
TD                 65536
SOLVENT            CDCl3
NS                     4
DS                     2
SWH            10000.000 Hz
FIDRES          0.305176 Hz
AQ             3.2767999 sec
RG                  56.3
DW                50.000 usec
DE                 13.89 usec
TE                 298.0 K
D1            1.00000000 sec
TD0                    1
SFO1         500.3030896 MHz
NUC1                  1H
P0                  3.33 usec
P1                 10.00 usec
PLW1         21.89999962 W

F2 - Processing parameters
SI                 65536
SF           500.3000000 MHz
WDW                   EM
SSB      0
LB                  0.30 Hz
GB       0
PC                  1.00
```

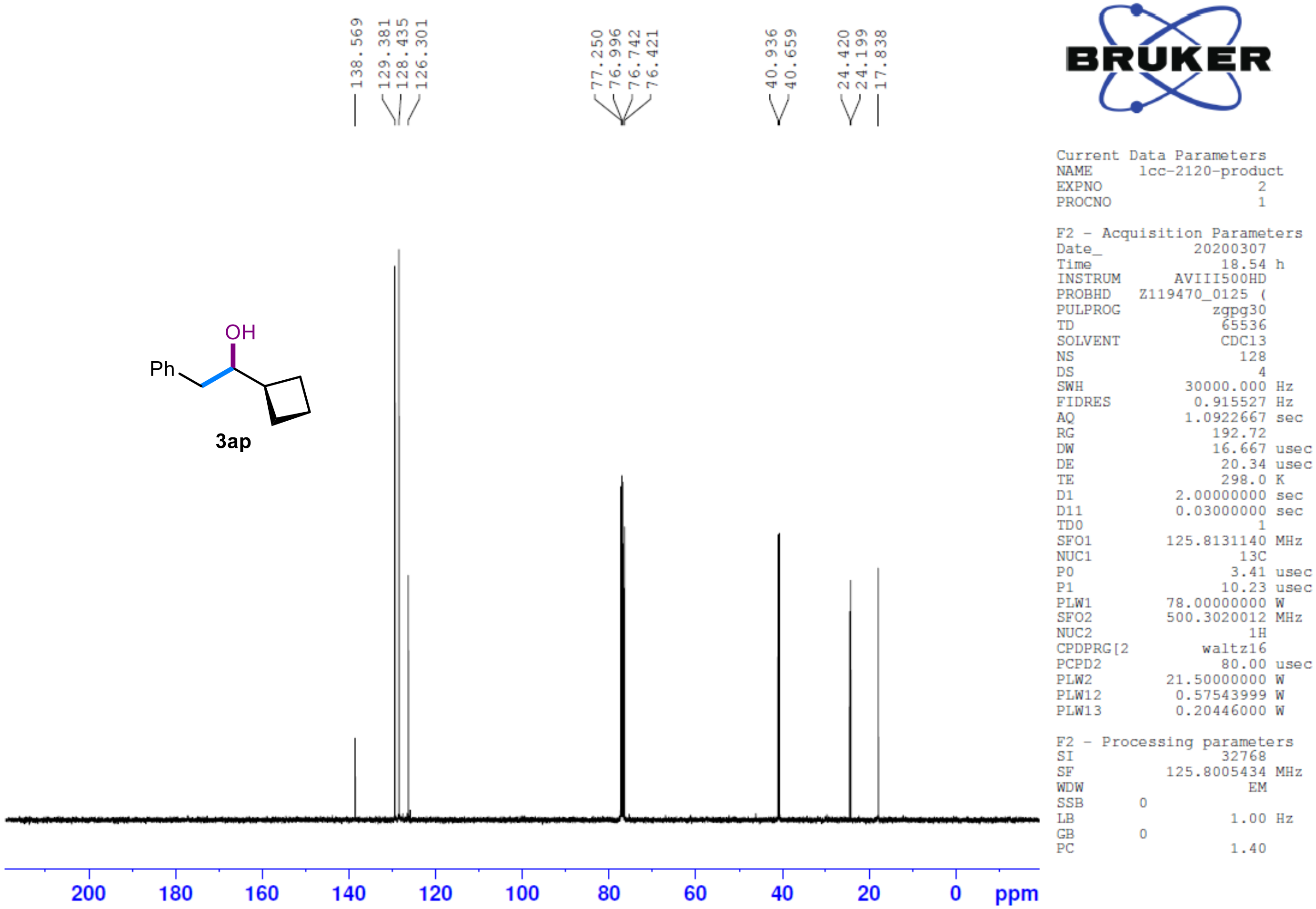

138.569
129.381
128.435
126.301
77.250
76.996
76.742
76.421
40.936
40.659
24.420
24.199
17.838
200 180 160 140 120 100 80 60 40 20 0 ppm
BRUKER
Current Data Parameters
NAME lcc-2120-product
EXPNO 2
PROCNO 1
F2 - Acquisition Parameters
Date_ 20200307
Time 18.54 h
INSTRUM AVIII500HD
PROBHD Z119470_0125 (
PULPROG zgpg30
TD 65536
SOLVENT CDCl3
NS 128
DS 4
SWH 30000.000 Hz
FIDRES 0.915527 Hz
AQ 1.0922667 sec
RG 192.72
DW 16.667 usec
DE 20.34 usec
TE 298.0 K
D1 2.00000000 sec
D11 0.03000000 sec
TD0 1
SFO1 125.8131140 MHz
NUC1 13C
P0 3.41 usec
P1 10.23 usec
PLW1 78.00000000 W
SFO2 500.3020012 MHz
NUC2 1H
CPDPRG[2 waltz16
PCPD2 80.00 usec
PLW2 21.50000000 W
PLW12 0.57543999 W
PLW13 0.20446000 W
F2 - Processing parameters
SI 32768
SF 125.8005434 MHz
WDW EM
SSB 0
LB 1.00 Hz
GB 0
PC 1.40
OH
Ph

3ap

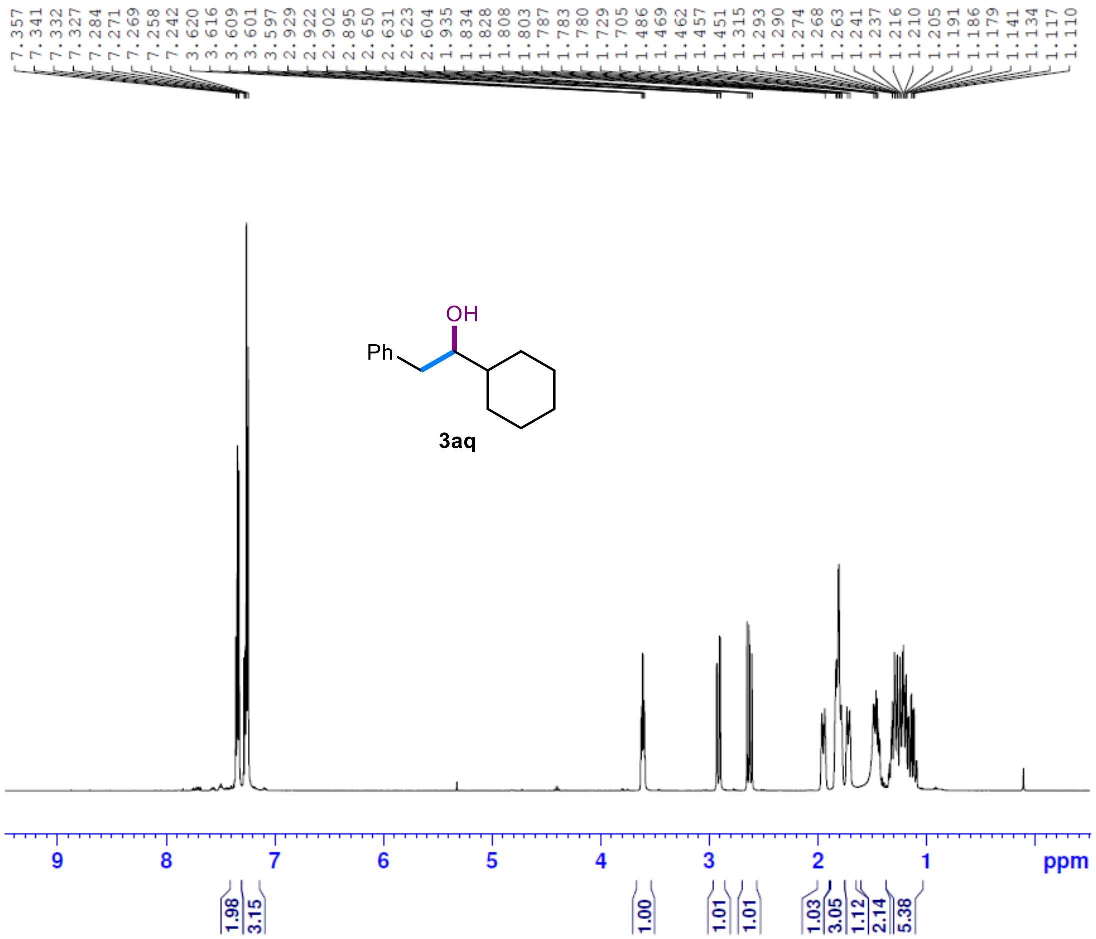

Current Data Parameters

NAME	lcc-2110-product
EXPNO	1
PROCNO	1

F2 - Acquisition Parameters

Date_	20200301
Time	23.18 h
INSTRUM	AVIII500HD
PROBHD	Z119470_0125 (
PULPROG	zg30
TD	65536
SOLVENT	CDCl3
NS	4
DS	2
SWH	10000.000 Hz
FIDRES	0.305176 Hz
AQ	3.2767999 sec
RG	77.88
DW	50.000 usec
DE	13.89 usec
TE	298.0 K
D1	1.00000000 sec
TD0	1
SFO1	500.3030896 MHz
NUC1	1H
P0	3.33 usec
P1	10.00 usec
PLW1	21.89999962 W

F2 - Processing parameters

SI	65536
SF	500.3000000 MHz
WDW	EM
SSB	0
LB	0.30 Hz
GB	0
PC	1.00

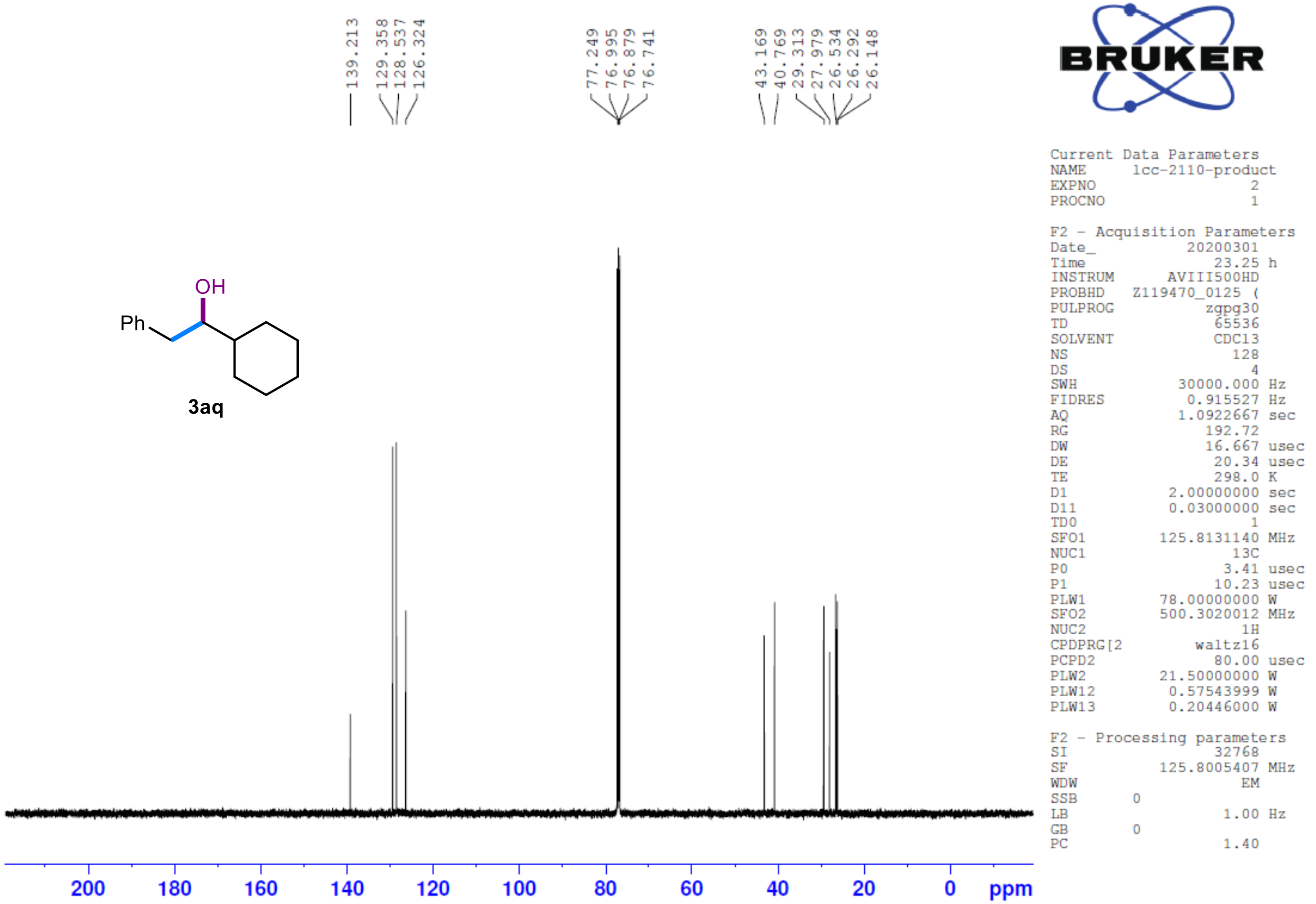
139.213
129.358
128.537
126.324
77.249
76.995
76.879
76.741
43.169
40.769
29.313
27.979
26.534
26.292
26.148
OH
Ph
3aq
200 180 160 140 120 100 80 60 40 20 0 ppm
BRUKER
Current Data Parameters
NAME lcc-2110-product
EXPNO 2
PROCNO 1
F2 - Acquisition Parameters
Date_ 20200301
Time 23.25 h
INSTRUM AVIII500HD
PROBHD Z119470_0125 (
PULPROG zgpg30
TD 65536
SOLVENT CDCl3
NS 128
DS 4
SWH 30000.000 Hz
FIDRES 0.915527 Hz
AQ 1.0922667 sec
RG 192.72
DW 16.667 usec
DE 20.34 usec
TE 298.0 K
D1 2.00000000 sec
D11 0.03000000 sec
TD0 1
SFO1 125.8131140 MHz
NUC1 13C
P0 3.41 usec
P1 10.23 usec
PLW1 78.00000000 W
SFO2 500.3020012 MHz
NUC2 1H
CPDPRG[2 waltz16
PCPD2 80.00 usec
PLW2 21.50000000 W
PLW12 0.57543999 W
PLW13 0.20446000 W
F2 - Processing parameters
SI 32768
SF 125.8005407 MHz
WDW EM
SSB 0
LB 1.00 Hz
GB 0
PC 1.40

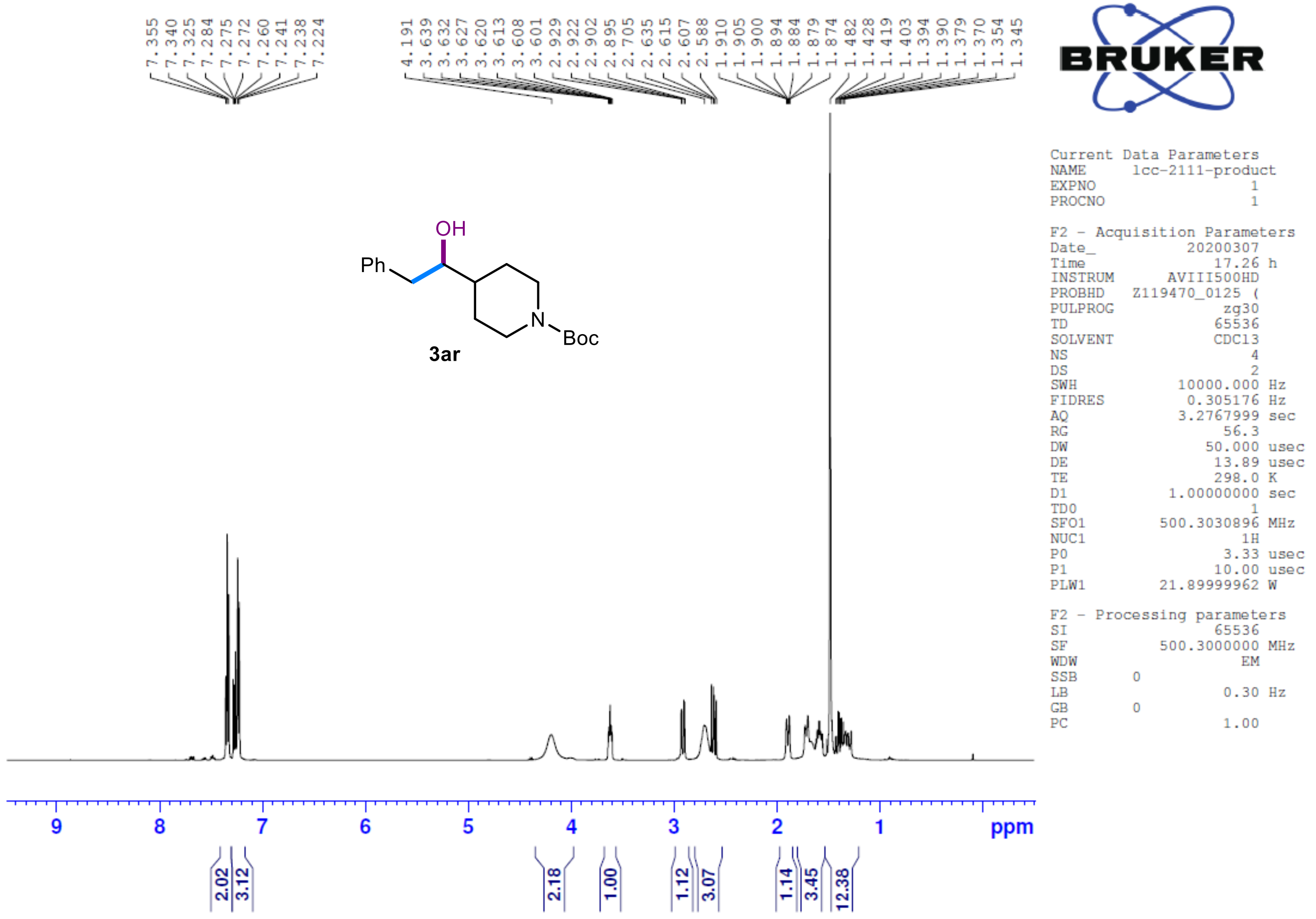
7.355
7.340
7.325
7.284
7.275
7.272
7.260
7.241
7.238
7.224
4.191
3.639
3.632
3.627
3.620
3.613
3.608
3.601
2.929
2.922
2.902
2.895
2.705
2.635
2.615
2.607
2.588
1.910
1.905
1.900
1.894
1.884
1.879
1.874
1.482
1.428
1.419
1.403
1.394
1.390
1.379
1.370
1.354
1.345
OH
Ph
N
Boc
3ar
BRUKER
Current Data Parameters
NAME lcc-2111-product
EXPNO 1
PROCNO 1
F2 - Acquisition Parameters
Date_ 20200307
Time 17.26 h
INSTRUM AVIII500HD
PROBHD Z119470_0125 (
PULPROG zg30
TD 65536
SOLVENT CDCl3
NS 4
DS 2
SWH 10000.000 Hz
FIDRES 0.305176 Hz
AQ 3.2767999 sec
RG 56.3
DW 50.000 usec
DE 13.89 usec
TE 298.0 K
D1 1.00000000 sec
TD0 1
SFO1 500.3030896 MHz
NUC1 1H
P0 3.33 usec
P1 10.00 usec
PLW1 21.89999962 W
F2 - Processing parameters
SI 65536
SF 500.3000000 MHz
WDW EM
SSB 0
LB 0.30 Hz
GB 0
PC 1.00
9
8
7
6
5
4
3
2
1
ppm
2.02
3.12
2.18
1.00
1.12
3.07
1.14
3.45
12.38

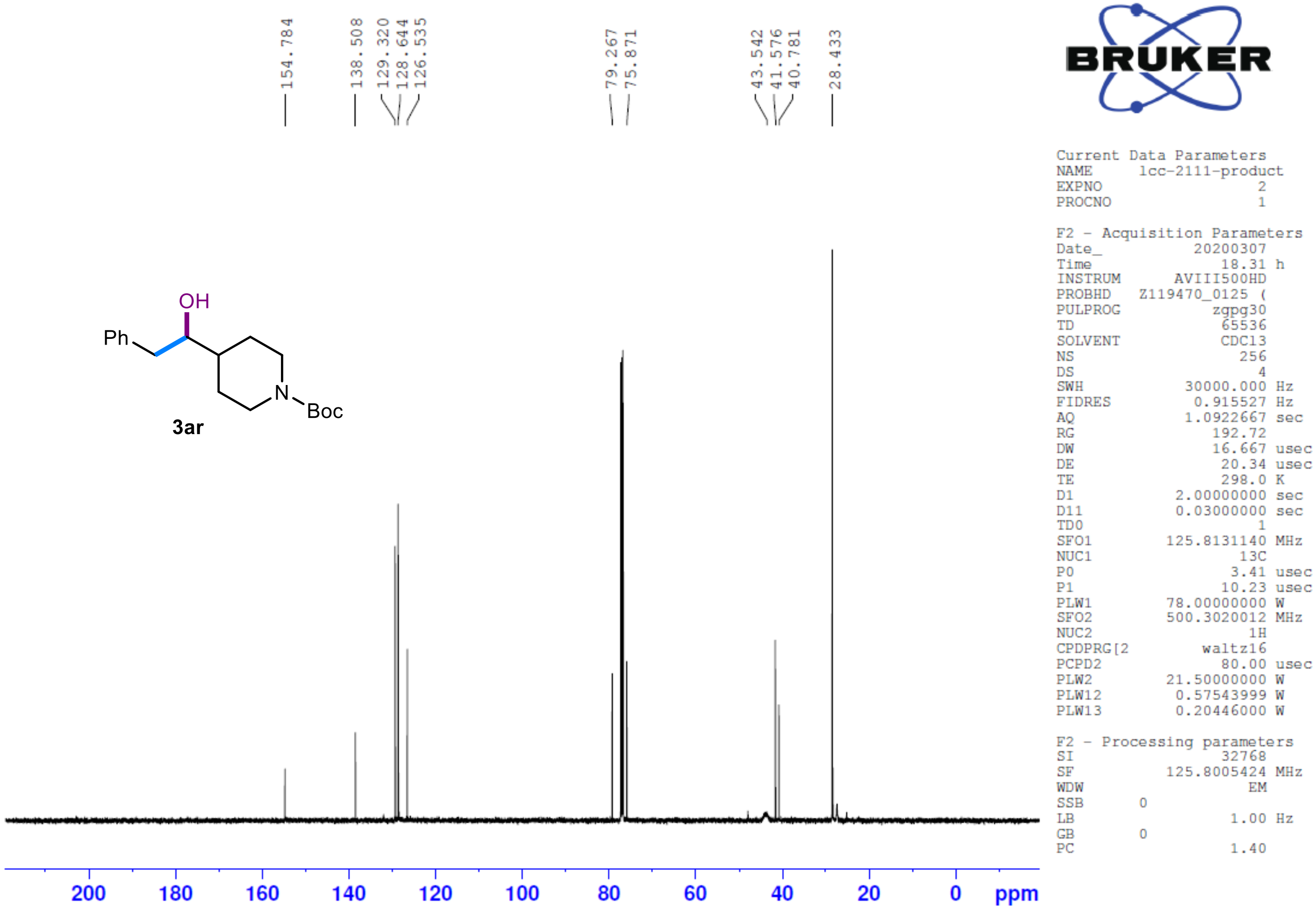

154.784
138.508
129.320
128.644
126.535
79.267
75.871
43.542
41.576
40.781
28.433
OH
Ph
N
Boc
3ar
200
180
160
140
120
100
80
60
40
20
0
ppm
BRUKER
Current Data Parameters
NAME lcc-2111-product
EXPNO 2
PROCNO 1
F2 - Acquisition Parameters
Date_ 20200307
Time 18.31 h
INSTRUM AVIII500HD
PROBHD Z119470_0125 (
PULPROG zgpg30
TD 65536
SOLVENT CDCl3
NS 256
DS 4
SWH 30000.000 Hz
FIDRES 0.915527 Hz
AQ 1.0922667 sec
RG 192.72
DW 16.667 usec
DE 20.34 usec
TE 298.0 K
D1 2.00000000 sec
D11 0.03000000 sec
TD0 1
SFO1 125.8131140 MHz
NUC1 13C
P0 3.41 usec
P1 10.23 usec
PLW1 78.00000000 W
SFO2 500.3020012 MHz
NUC2 1H
CPDPRG[2 waltz16
PCPD2 80.00 usec
PLW2 21.50000000 W
PLW12 0.57543999 W
PLW13 0.20446000 W
F2 - Processing parameters
SI 32768
SF 125.8005424 MHz
WDW EM
SSB 0
LB 1.00 Hz
GB 0
PC 1.40

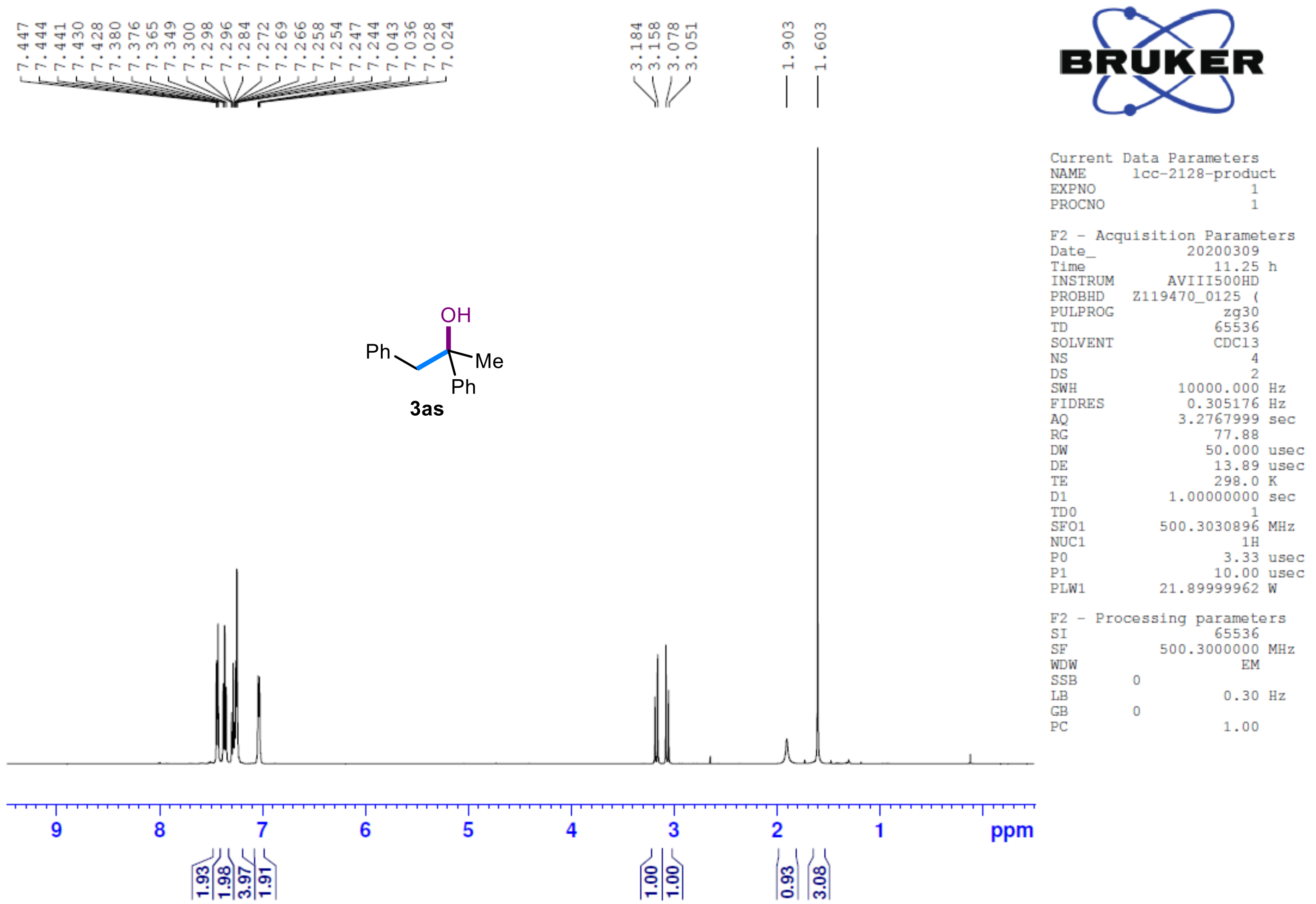

7.447 7.444 7.441 7.430 7.428 7.380 7.376 7.365 7.349 7.300 7.298 7.296 7.284 7.272 7.269 7.266 7.258 7.254 7.247 7.244 7.043 7.036 7.028 7.024
3.184 3.158 3.078 3.051
1.903
1.603
OH
Ph
Me
Ph
3as
9 8 7 6 5 4 3 2 1 ppm
1.93 1.98 3.97 1.91 1.00 1.00 0.93 3.08
BRUKER
Current Data Parameters
NAME lcc-2128-product
EXPNO 1
PROCNO 1
F2 - Acquisition Parameters
Date_ 20200309
Time 11.25 h
INSTRUM AVIII500HD
PROBHD Z119470_0125 (
PULPROG zg30
TD 65536
SOLVENT CDCl3
NS 4
DS 2
SWH 10000.000 Hz
FIDRES 0.305176 Hz
AQ 3.2767999 sec
RG 77.88
DW 50.000 usec
DE 13.89 usec
TE 298.0 K
D1 1.00000000 sec
TD0 1
SFO1 500.3030896 MHz
NUC1 1H
P0 3.33 usec
P1 10.00 usec
PLW1 21.89999962 W
F2 - Processing parameters
SI 65536
SF 500.3000000 MHz
WDW EM
SSB 0
LB 0.30 Hz
GB 0
PC 1.00

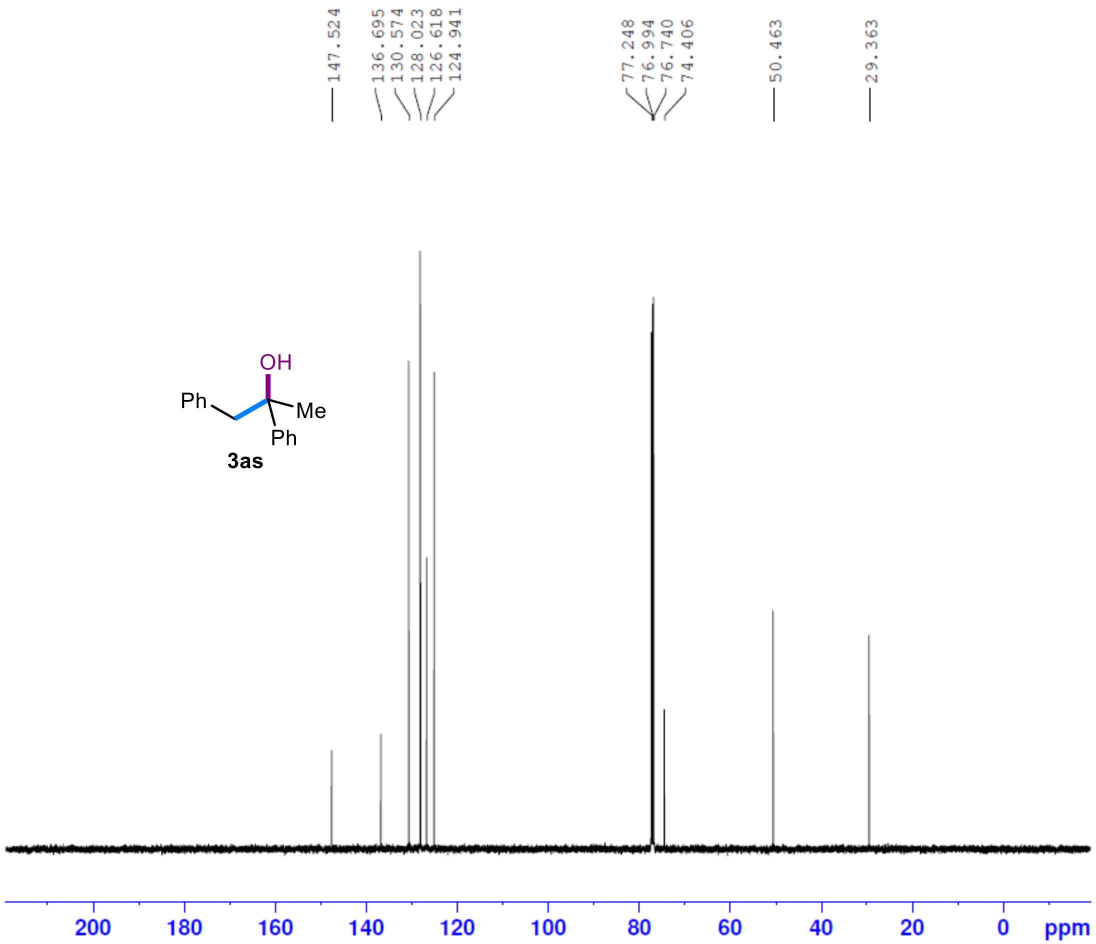


```
Current Data Parameters
NAME      lcc-2128-product
EXPNO                    2
PROCNO                   1

F2 - Acquisition Parameters
Date_             20200309
Time                 11.32 h
INSTRUM         AVIII500HD
PROBHD    Z119470_0125 (
PULPROG            zgpg30
TD                  65536
SOLVENT             CDCl3
NS                    128
DS                      4
SWH             30000.000 Hz
FIDRES           0.915527 Hz
AQ              1.0922667 sec
RG                 192.72
DW                 16.667 usec
DE                  20.34 usec
TE                  298.0 K
D1             2.00000000 sec
D11            0.03000000 sec
TD0                     1
SFO1          125.8131140 MHz
NUC1                  13C
P0                   3.41 usec
P1                  10.23 usec
PLW1          78.00000000 W
SFO2          500.3020012 MHz
NUC2                   1H
CPDPRG[2          waltz16
PCPD2               80.00 usec
PLW2          21.50000000 W
PLW12          0.57543999 W
PLW13          0.20446000 W

F2 - Processing parameters
SI                  32768
SF            125.8005425 MHz
WDW                    EM
SSB      0
LB                   1.00 Hz
GB       0
PC                   1.40
```

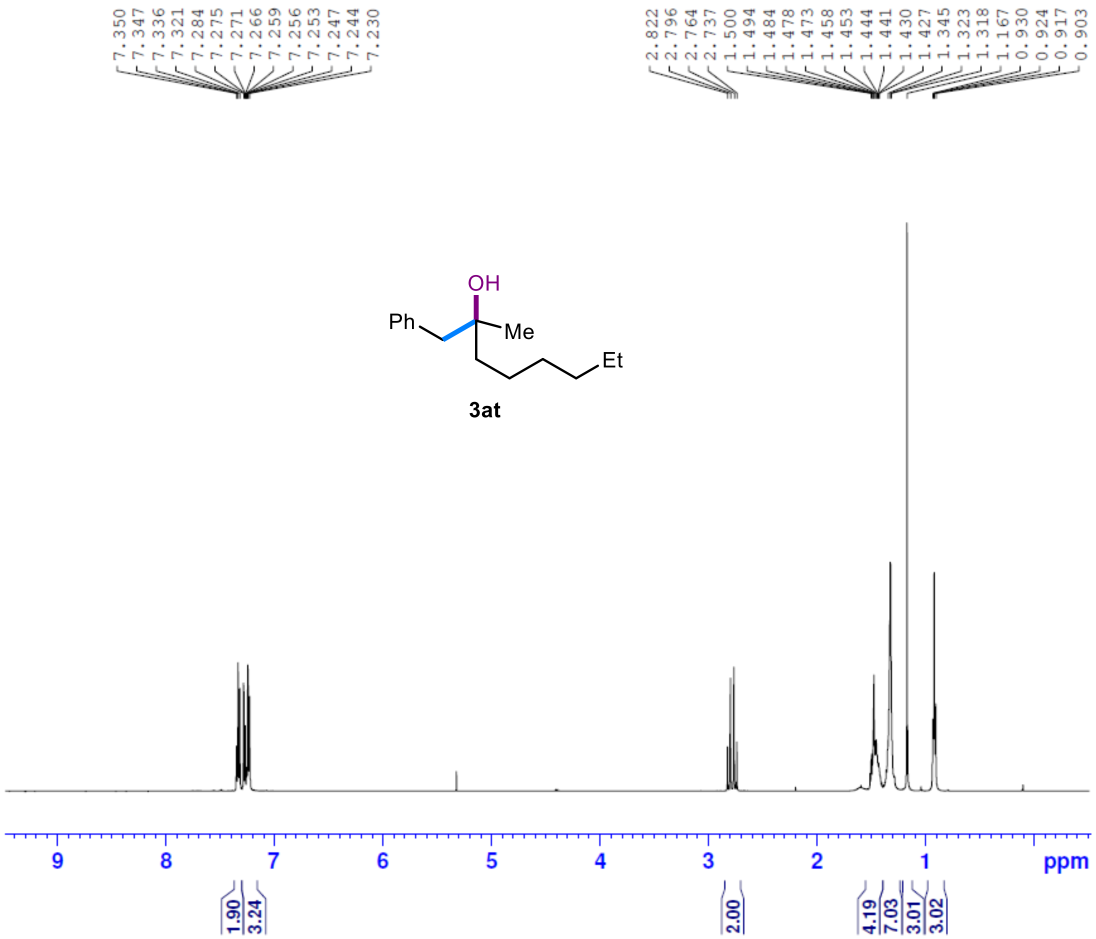

3at

Current Data Parameters
NAME lcc-2130-product
EXPNO 1
PROCNO 1

F2 - Acquisition Parameters
Date_ 20200313
Time 8.06 h
INSTRUM AVIII500HD
PROBHD Z119470_0125 (
PULPROG zg30
TD 65536
SOLVENT CDCl3
NS 16
DS 2
SWH 10000.000 Hz
FIDRES 0.305176 Hz
AQ 3.2767999 sec
RG 106.56
DW 50.000 usec
DE 13.89 usec
TE 298.0 K
D1 1.00000000 sec
TD0 1
SFO1 500.3030896 MHz
NUC1 1H
P0 3.33 usec
P1 10.00 usec
PLW1 21.89999962 W

F2 - Processing parameters
SI 65536
SF 500.3000000 MHz
WDW EM
SSB 0
LB 0.30 Hz
GB 0
PC 1.00

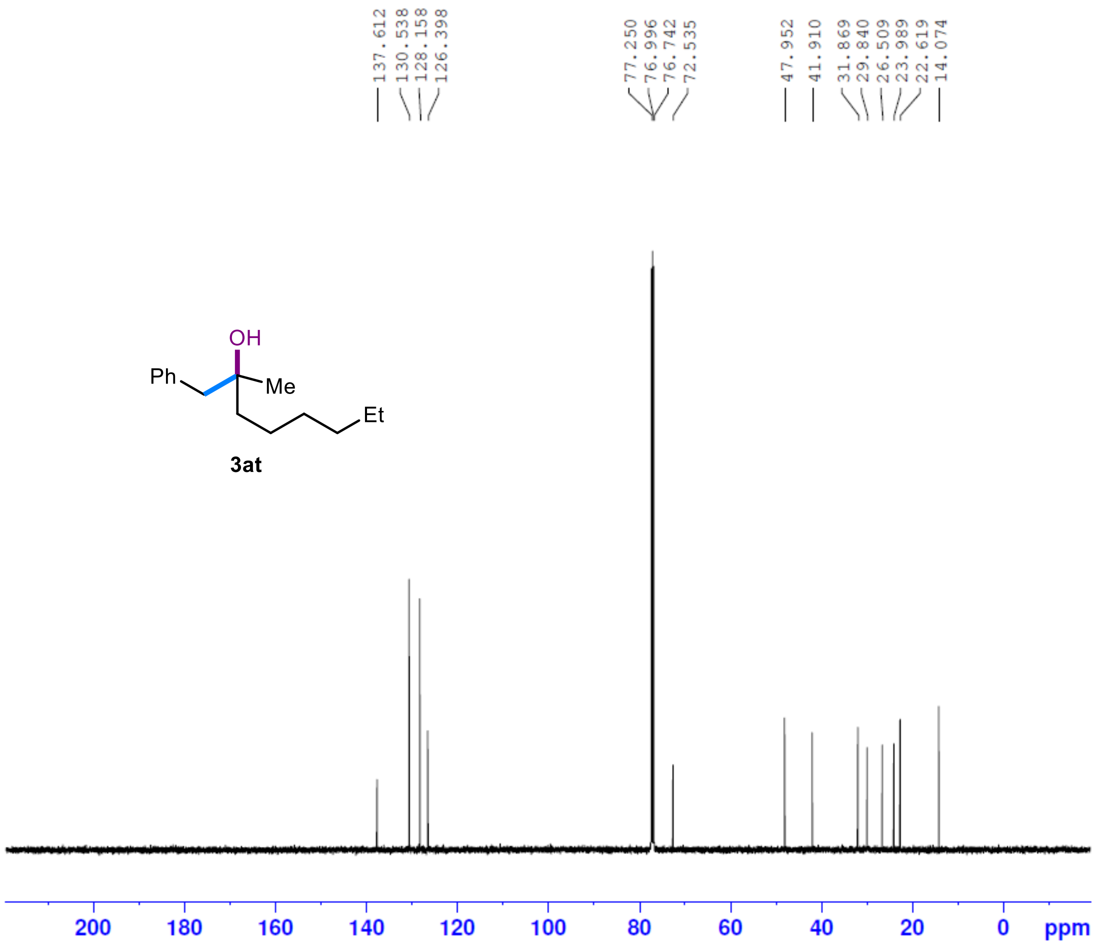


```
Current Data Parameters
NAME       lcc-2130-product
EXPNO                     2
PROCNO                    1

F2 - Acquisition Parameters
Date_              20200313
Time                   8.21 h
INSTRUM          AVIII500HD
PROBHD     Z119470_0125 (
PULPROG             zgpg30
TD                   65536
SOLVENT              CDCl3
NS                     256
DS                       4
SWH              30000.000 Hz
FIDRES            0.915527 Hz
AQ               1.0922667 sec
RG                  192.72
DW                  16.667 usec
DE                   20.34 usec
TE                   298.0 K
D1              2.00000000 sec
D11             0.03000000 sec
TD0                      1
SFO1           125.8131140 MHz
NUC1                   13C
P0                    3.41 usec
P1                   10.23 usec
PLW1           78.00000000 W
SFO2           500.3020012 MHz
NUC2                    1H
CPDPRG[2           waltz16
PCPD2                80.00 usec
PLW2           21.50000000 W
PLW12           0.57543999 W
PLW13           0.20446000 W

F2 - Processing parameters
SI                   32768
SF             125.8005389 MHz
WDW                     EM
SSB       0
LB                    1.00 Hz
GB        0
PC                    1.40
```

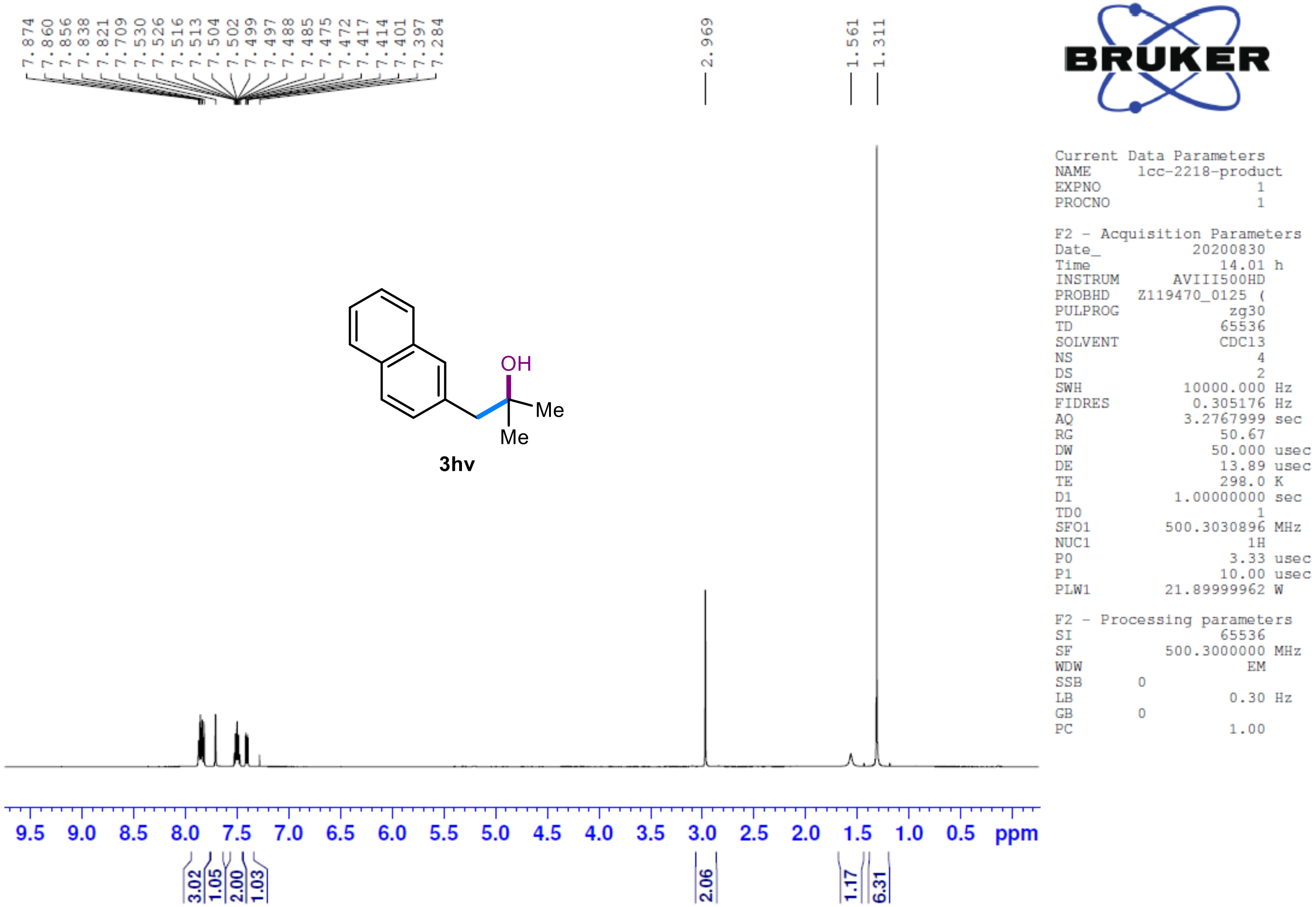
7.874
7.860
7.856
7.838
7.821
7.709
7.530
7.526
7.516
7.513
7.504
7.502
7.499
7.497
7.488
7.485
7.475
7.472
7.417
7.414
7.401
7.397
7.284
2.969
1.561
1.311
BRUKER
OH
Me
Me
3hv
Current Data Parameters
NAME lcc-2218-product
EXPNO 1
PROCNO 1
F2 - Acquisition Parameters
Date_ 20200830
Time 14.01 h
INSTRUM AVIII500HD
PROBHD Z119470_0125 (
PULPROG zg30
TD 65536
SOLVENT CDCl3
NS 4
DS 2
SWH 10000.000 Hz
FIDRES 0.305176 Hz
AQ 3.2767999 sec
RG 50.67
DW 50.000 usec
DE 13.89 usec
TE 298.0 K
D1 1.00000000 sec
TD0 1
SFO1 500.3030896 MHz
NUC1 1H
P0 3.33 usec
P1 10.00 usec
PLW1 21.89999962 W
F2 - Processing parameters
SI 65536
SF 500.3000000 MHz
WDW EM
SSB 0
LB 0.30 Hz
GB 0
PC 1.00
9.5 9.0 8.5 8.0 7.5 7.0 6.5 6.0 5.5 5.0 4.5 4.0 3.5 3.0 2.5 2.0 1.5 1.0 0.5 ppm
3.02
1.05
2.00
1.03
2.06
1.17
6.31

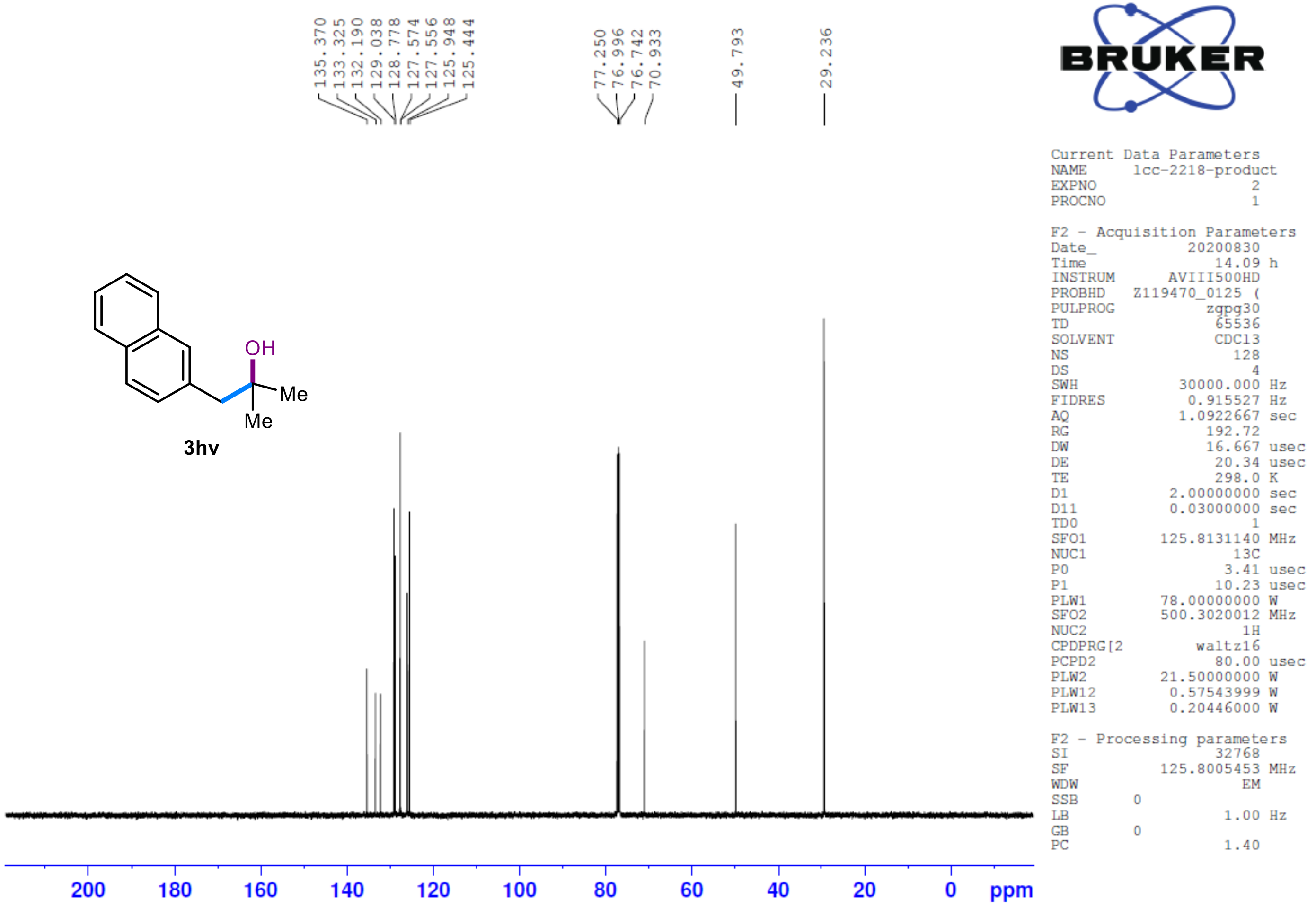
135.370
133.325
132.190
129.038
128.778
127.574
127.556
125.948
125.444
77.250
76.996
76.742
70.933
49.793
29.236
OH
Me
Me
3hv
200
180
160
140
120
100
80
60
40
20
0
ppm
Current Data Parameters
NAME lcc-2218-product
EXPNO 2
PROCNO 1
F2 - Acquisition Parameters
Date_ 20200830
Time 14.09 h
INSTRUM AVIII500HD
PROBHD Z119470_0125 (
PULPROG zgpg30
TD 65536
SOLVENT CDCl3
NS 128
DS 4
SWH 30000.000 Hz
FIDRES 0.915527 Hz
AQ 1.0922667 sec
RG 192.72
DW 16.667 usec
DE 20.34 usec
TE 298.0 K
D1 2.00000000 sec
D11 0.03000000 sec
TD0 1
SFO1 125.8131140 MHz
NUC1 13C
P0 3.41 usec
P1 10.23 usec
PLW1 78.00000000 W
SFO2 500.3020012 MHz
NUC2 1H
CPDPRG[2 waltz16
PCPD2 80.00 usec
PLW2 21.50000000 W
PLW12 0.57543999 W
PLW13 0.20446000 W
F2 - Processing parameters
SI 32768
SF 125.8005453 MHz
WDW EM
SSB 0
LB 1.00 Hz
GB 0
PC 1.40

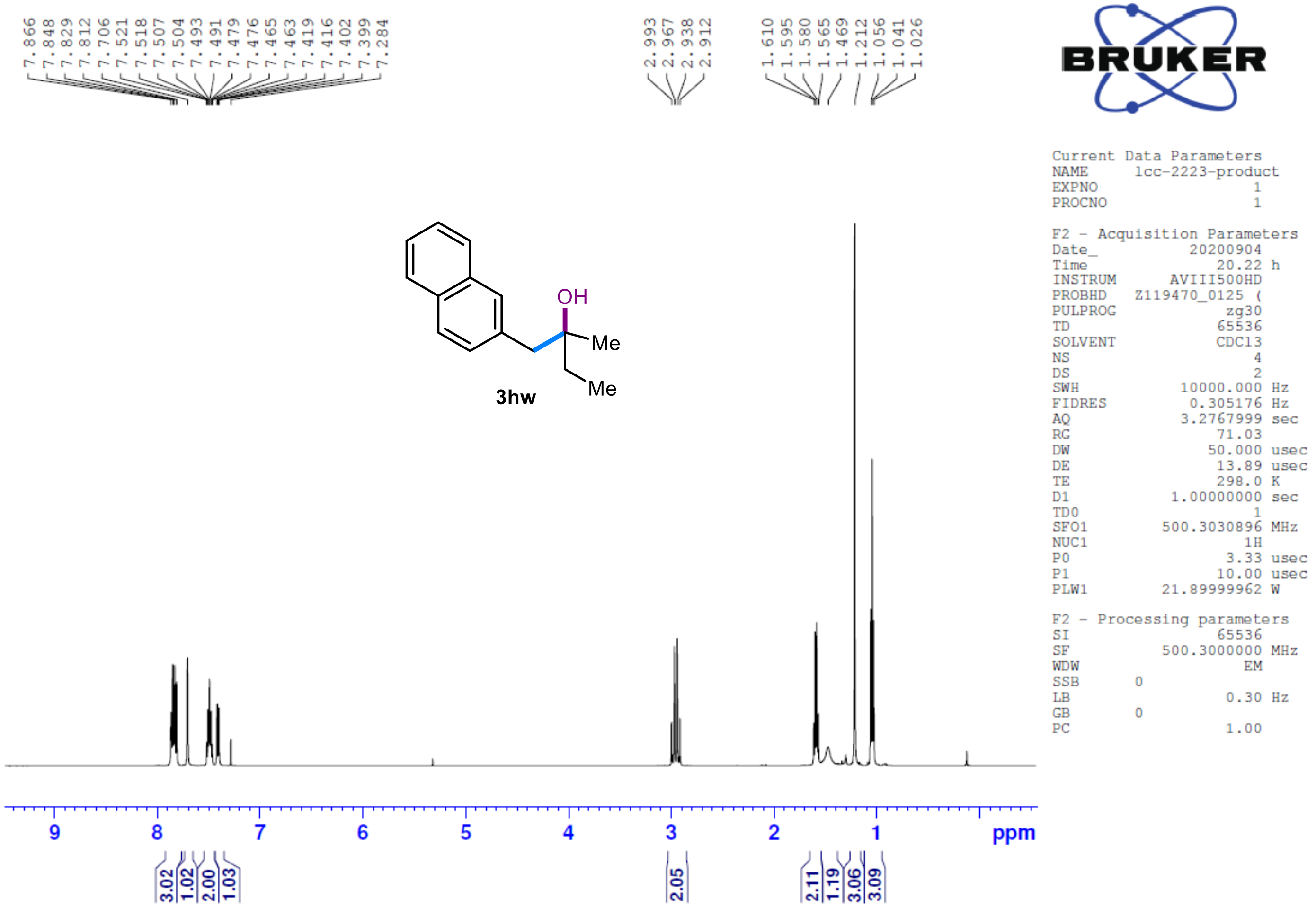
7.866
7.848
7.829
7.812
7.706
7.521
7.518
7.507
7.504
7.493
7.491
7.479
7.476
7.465
7.463
7.419
7.416
7.402
7.399
7.284
2.993
2.967
2.938
2.912
1.610
1.595
1.580
1.565
1.469
1.212
1.056
1.041
1.026
BRUKER
OH
Me
Me
3hw
Current Data Parameters
NAME lcc-2223-product
EXPNO 1
PROCNO 1
F2 - Acquisition Parameters
Date_ 20200904
Time 20.22 h
INSTRUM AVIII500HD
PROBHD Z119470_0125 (
PULPROG zg30
TD 65536
SOLVENT CDCl3
NS 4
DS 2
SWH 10000.000 Hz
FIDRES 0.305176 Hz
AQ 3.2767999 sec
RG 71.03
DW 50.000 usec
DE 13.89 usec
TE 298.0 K
D1 1.00000000 sec
TD0 1
SFO1 500.3030896 MHz
NUC1 1H
P0 3.33 usec
P1 10.00 usec
PLW1 21.89999962 W
F2 - Processing parameters
SI 65536
SF 500.3000000 MHz
WDW EM
SSB 0
LB 0.30 Hz
GB 0
PC 1.00
9
8
7
6
5
4
3
2
1
ppm
3.02
1.02
2.00
1.03
2.05
2.11
1.19
3.06
3.09

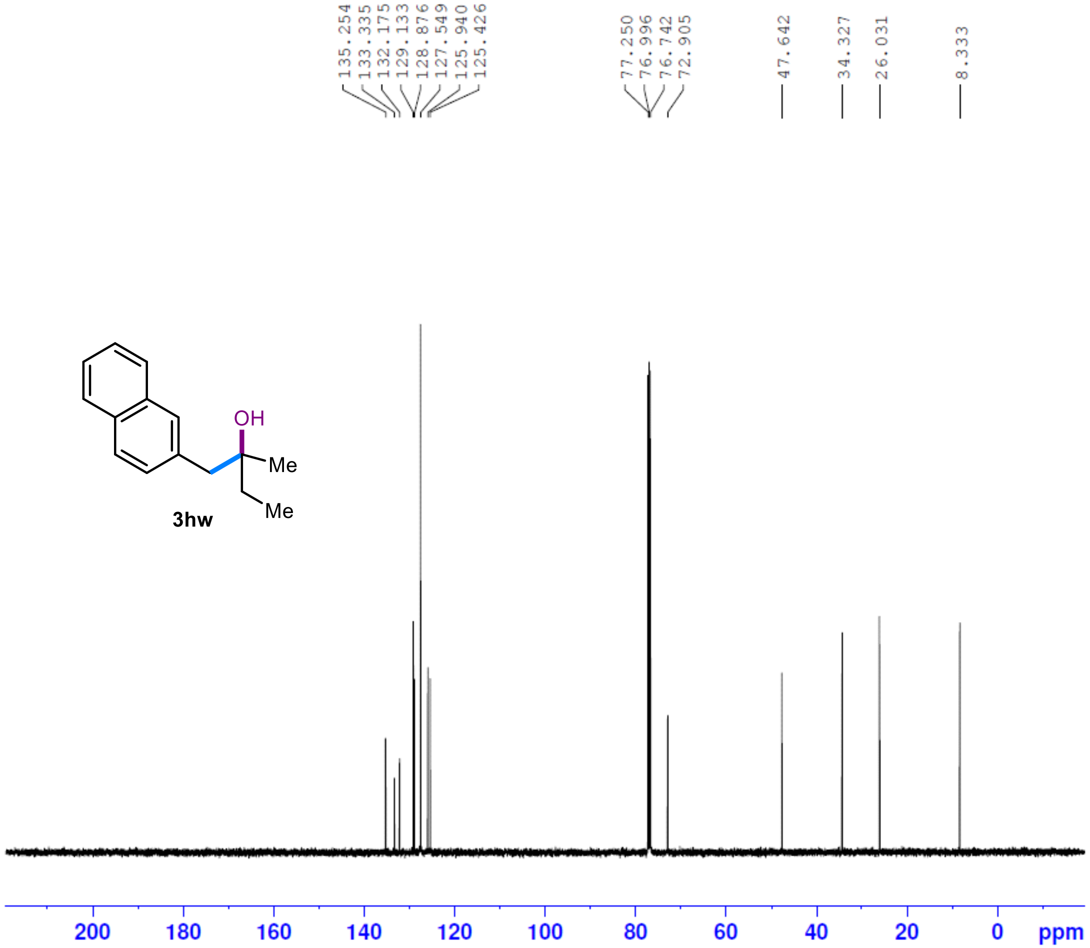


```
Current Data Parameters
NAME        lcc-2223-product
EXPNO                      2
PROCNO                     1

F2 - Acquisition Parameters
Date_               20200904
Time                   20.30 h
INSTRUM           AVIII500HD
PROBHD      Z119470_0125 (
PULPROG              zgpg30
TD                     65536
SOLVENT                CDCl3
NS                       128
DS                         4
SWH                30000.000 Hz
FIDRES              0.915527 Hz
AQ                 1.0922667 sec
RG                    192.72
DW                    16.667 usec
DE                     20.34 usec
TE                     298.0 K
D1                2.00000000 sec
D11               0.03000000 sec
TD0                        1
SFO1             125.8131140 MHz
NUC1                     13C
P0                      3.41 usec
P1                     10.23 usec
PLW1             78.00000000 W
SFO2             500.3020012 MHz
NUC2                      1H
CPDPRG[2             waltz16
PCPD2                  80.00 usec
PLW2             21.50000000 W
PLW12             0.57543999 W
PLW13             0.20446000 W

F2 - Processing parameters
SI                     32768
SF               125.8005425 MHz
WDW                       EM
SSB      0
LB                      1.00 Hz
GB       0
PC                      1.40
```

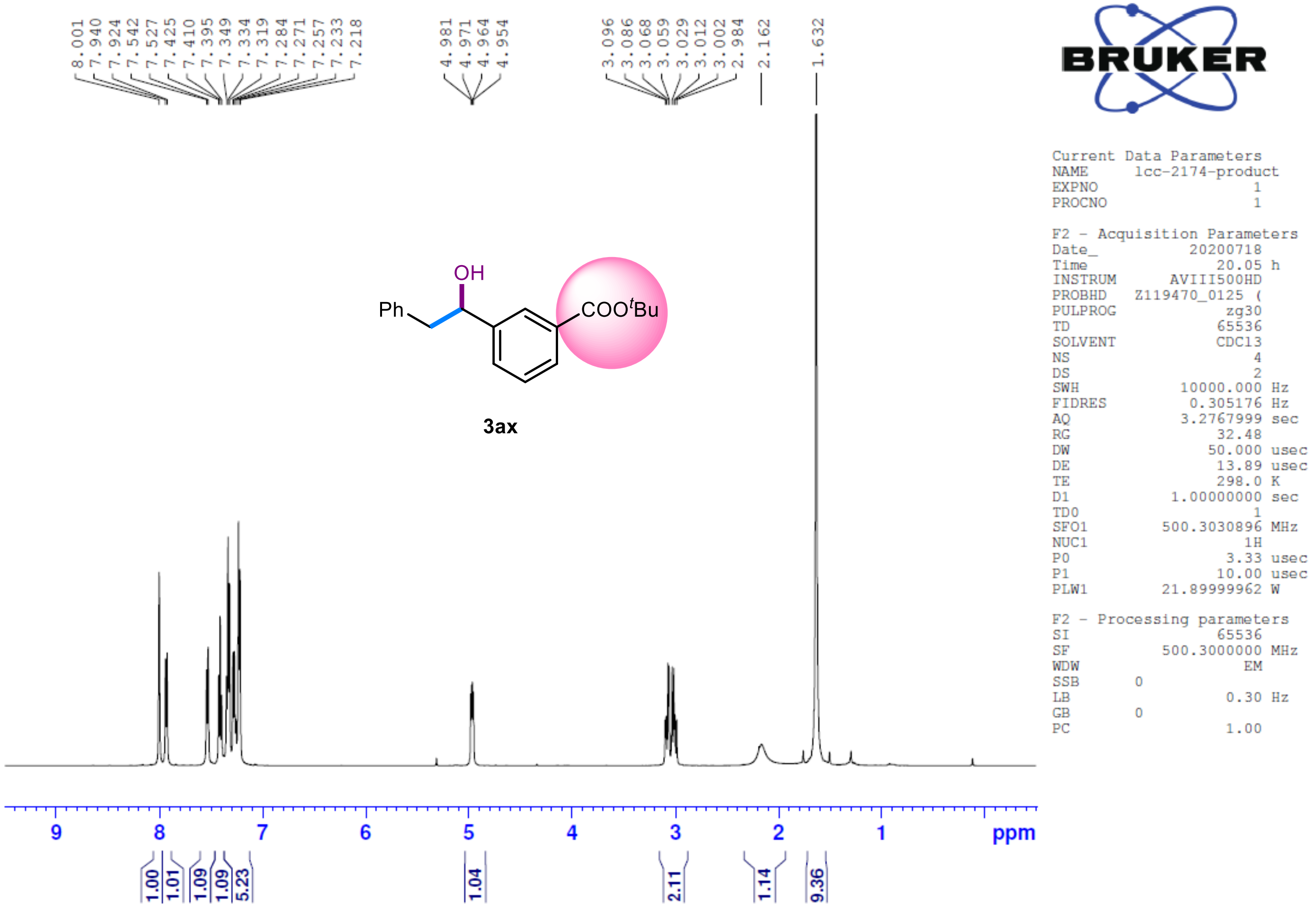
8.001 7.940 7.924 7.542 7.527 7.425 7.410 7.395 7.349 7.334 7.319 7.284 7.271 7.257 7.233 7.218
4.981 4.971 4.964 4.954
3.096 3.086 3.068 3.059 3.029 3.012 3.002 2.984
2.162
1.632
OH
Ph
COO^{t}Bu
3ax
BRUKER
Current Data Parameters
NAME lcc-2174-product
EXPNO 1
PROCNO 1
F2 - Acquisition Parameters
Date_ 20200718
Time 20.05 h
INSTRUM AVIII500HD
PROBHD Z119470_0125 (
PULPROG zg30
TD 65536
SOLVENT CDCl3
NS 4
DS 2
SWH 10000.000 Hz
FIDRES 0.305176 Hz
AQ 3.2767999 sec
RG 32.48
DW 50.000 usec
DE 13.89 usec
TE 298.0 K
D1 1.00000000 sec
TD0 1
SFO1 500.3030896 MHz
NUC1 1H
P0 3.33 usec
P1 10.00 usec
PLW1 21.89999962 W
F2 - Processing parameters
SI 65536
SF 500.3000000 MHz
WDW EM
SSB 0
LB 0.30 Hz
GB 0
PC 1.00
9 8 7 6 5 4 3 2 1 ppm
1.00 1.01 1.09 1.09 5.23 1.04 2.11 1.14 9.36

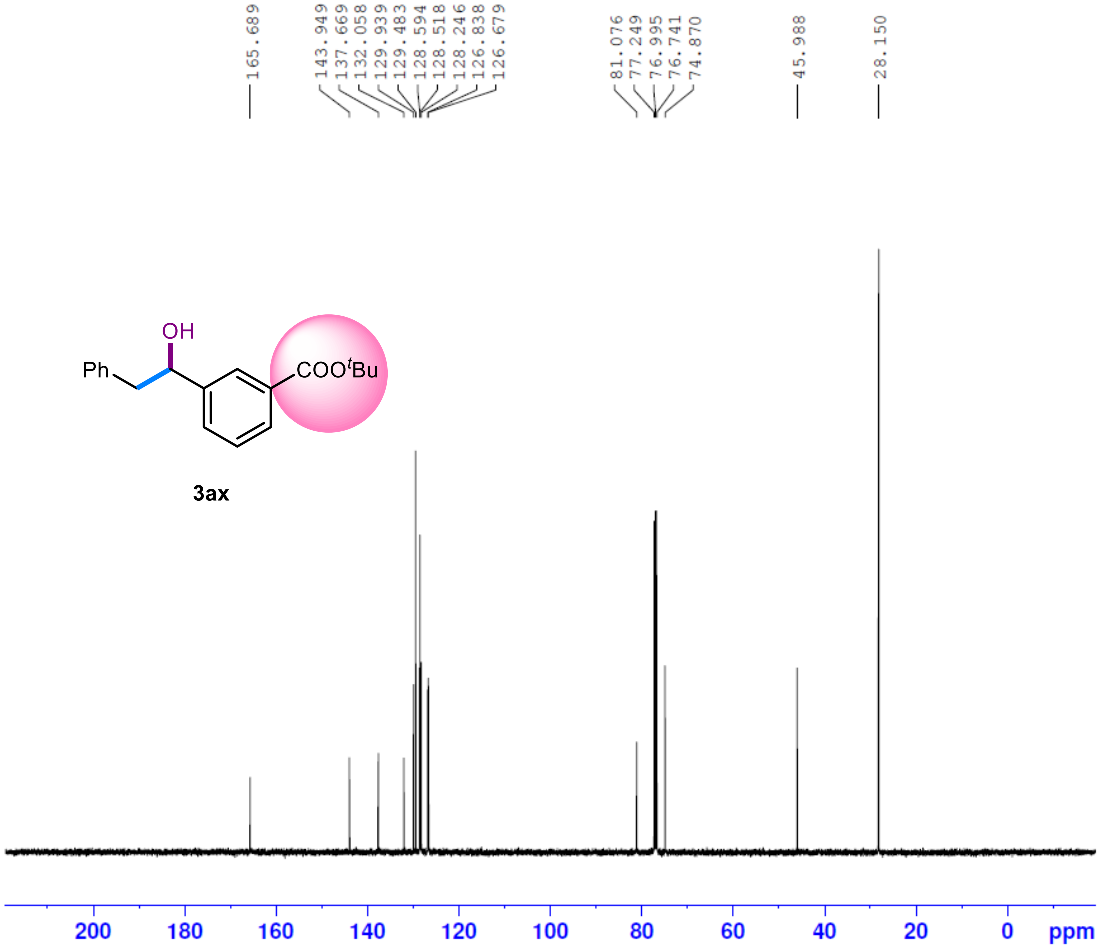

3ax

BRUKER

Current Data Parameters
NAME lcc-2174-product
EXPNO 2
PROCNO 1

F2 - Acquisition Parameters
Date_ 20200718
Time 20.13 h
INSTRUM AVIII500HD
PROBHD Z119470_0125 (
PULPROG zgpg30
TD 65536
SOLVENT CDCl3
NS 128
DS 4
SWH 30000.000 Hz
FIDRES 0.915527 Hz
AQ 1.0922667 sec
RG 192.72
DW 16.667 usec
DE 20.34 usec
TE 298.0 K
D1 2.00000000 sec
D11 0.03000000 sec
TD0 1
SFO1 125.8131140 MHz
NUC1 13C
P0 3.41 usec
P1 10.23 usec
PLW1 78.00000000 W
SFO2 500.3020012 MHz
NUC2 1H
CPDPRG[2 waltz16
PCPD2 80.00 usec
PLW2 21.50000000 W
PLW12 0.57543999 W
PLW13 0.20446000 W

F2 - Processing parameters
SI 32768
SF 125.8005443 MHz
WDW EM
SSB 0
LB 1.00 Hz
GB 0
PC 1.40

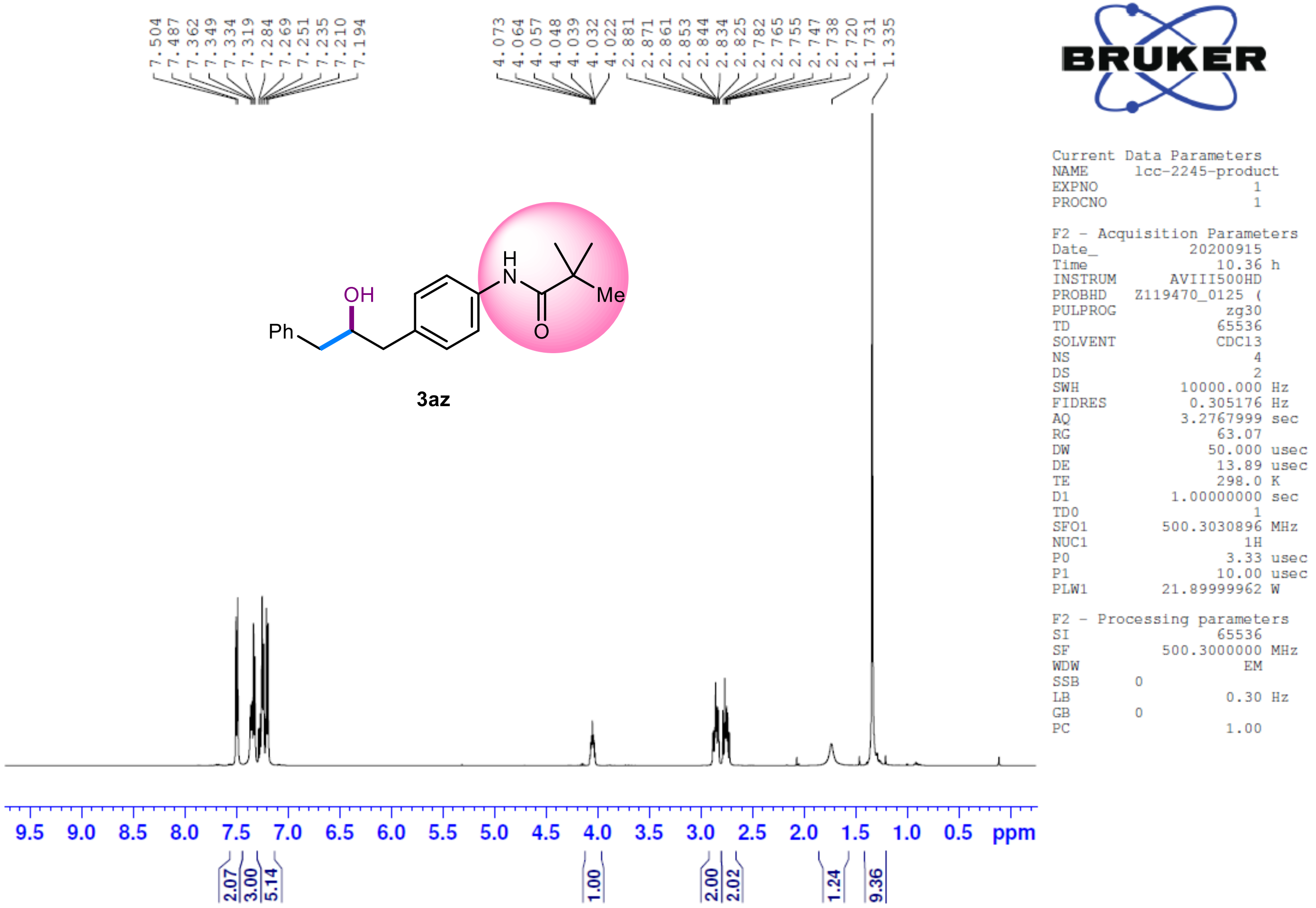
7.504
7.487
7.362
7.349
7.334
7.319
7.284
7.269
7.251
7.235
7.210
7.194
4.073
4.064
4.057
4.048
4.039
4.032
4.022
2.881
2.871
2.861
2.853
2.844
2.834
2.825
2.782
2.765
2.755
2.747
2.738
2.720
1.731
1.335
OH
Ph
H
N
O
Me
3az
Current Data Parameters
NAME lcc-2245-product
EXPNO 1
PROCNO 1
F2 - Acquisition Parameters
Date_ 20200915
Time 10.36 h
INSTRUM AVIII500HD
PROBHD Z119470_0125 (
PULPROG zg30
TD 65536
SOLVENT CDCl3
NS 4
DS 2
SWH 10000.000 Hz
FIDRES 0.305176 Hz
AQ 3.2767999 sec
RG 63.07
DW 50.000 usec
DE 13.89 usec
TE 298.0 K
D1 1.00000000 sec
TD0 1
SFO1 500.3030896 MHz
NUC1 1H
P0 3.33 usec
P1 10.00 usec
PLW1 21.89999962 W
F2 - Processing parameters
SI 65536
SF 500.3000000 MHz
WDW EM
SSB 0
LB 0.30 Hz
GB 0
PC 1.00
9.5 9.0 8.5 8.0 7.5 7.0 6.5 6.0 5.5 5.0 4.5 4.0 3.5 3.0 2.5 2.0 1.5 1.0 0.5 ppm
2.07
3.00
5.14
1.00
2.00
2.02
1.24
9.36

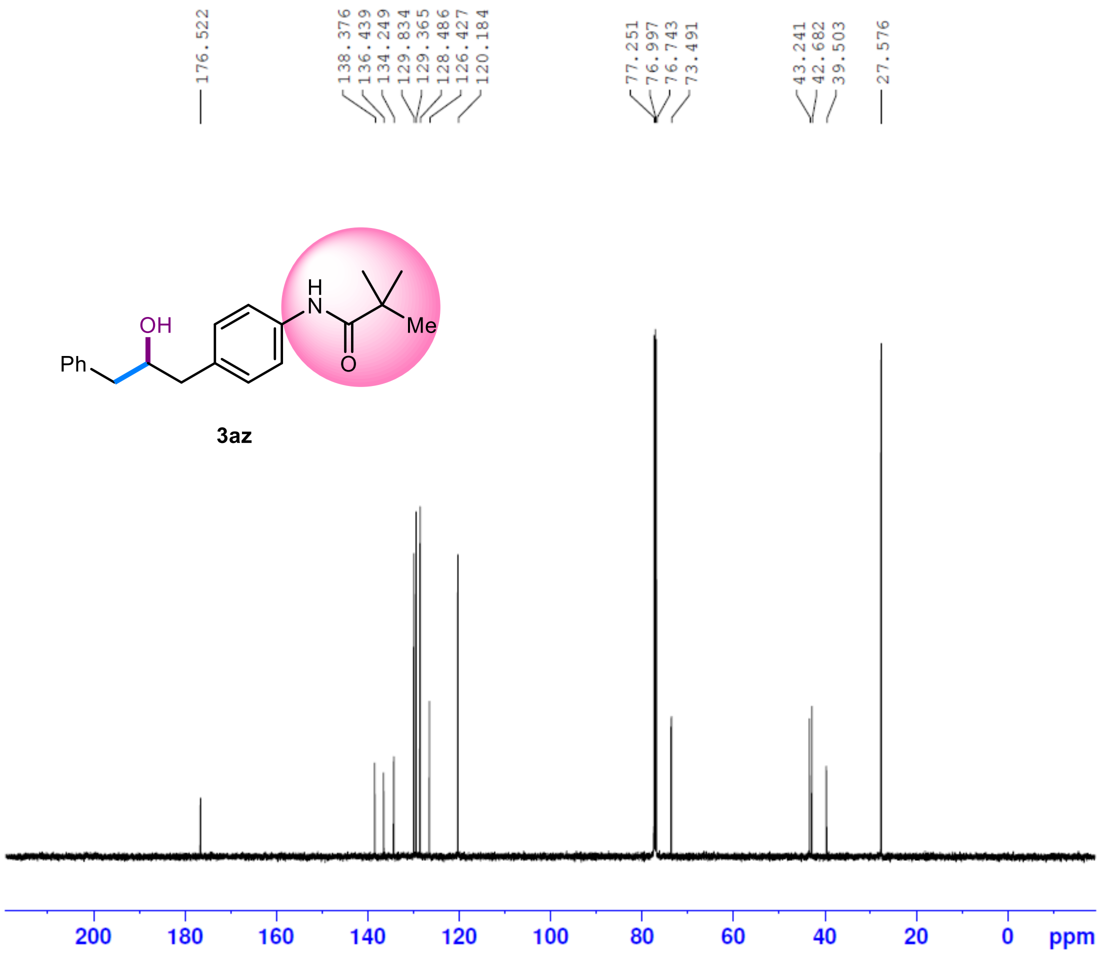


```
Current Data Parameters
NAME        lcc-2245-product
EXPNO                      2
PROCNO                     1

F2 - Acquisition Parameters
Date_               20200915
Time                   10.43 h
INSTRUM           AVIII500HD
PROBHD      Z119470_0125 (
PULPROG              zgpg30
TD                    65536
SOLVENT               CDCl3
NS                      128
DS                        4
SWH               30000.000 Hz
FIDRES             0.915527 Hz
AQ                1.0922667 sec
RG                   192.72
DW                   16.667 usec
DE                    20.34 usec
TE                    298.0 K
D1               2.00000000 sec
D11              0.03000000 sec
TD0                       1
SFO1            125.8131140 MHz
NUC1                    13C
P0                     3.41 usec
P1                    10.23 usec
PLW1            78.00000000 W
SFO2            500.3020012 MHz
NUC2                     1H
CPDPRG[2            waltz16
PCPD2                 80.00 usec
PLW2            21.50000000 W
PLW12            0.57543999 W
PLW13            0.20446000 W

F2 - Processing parameters
SI                    32768
SF              125.8005443 MHz
WDW                      EM
SSB      0
LB                     1.00 Hz
GB       0
PC                     1.40
```

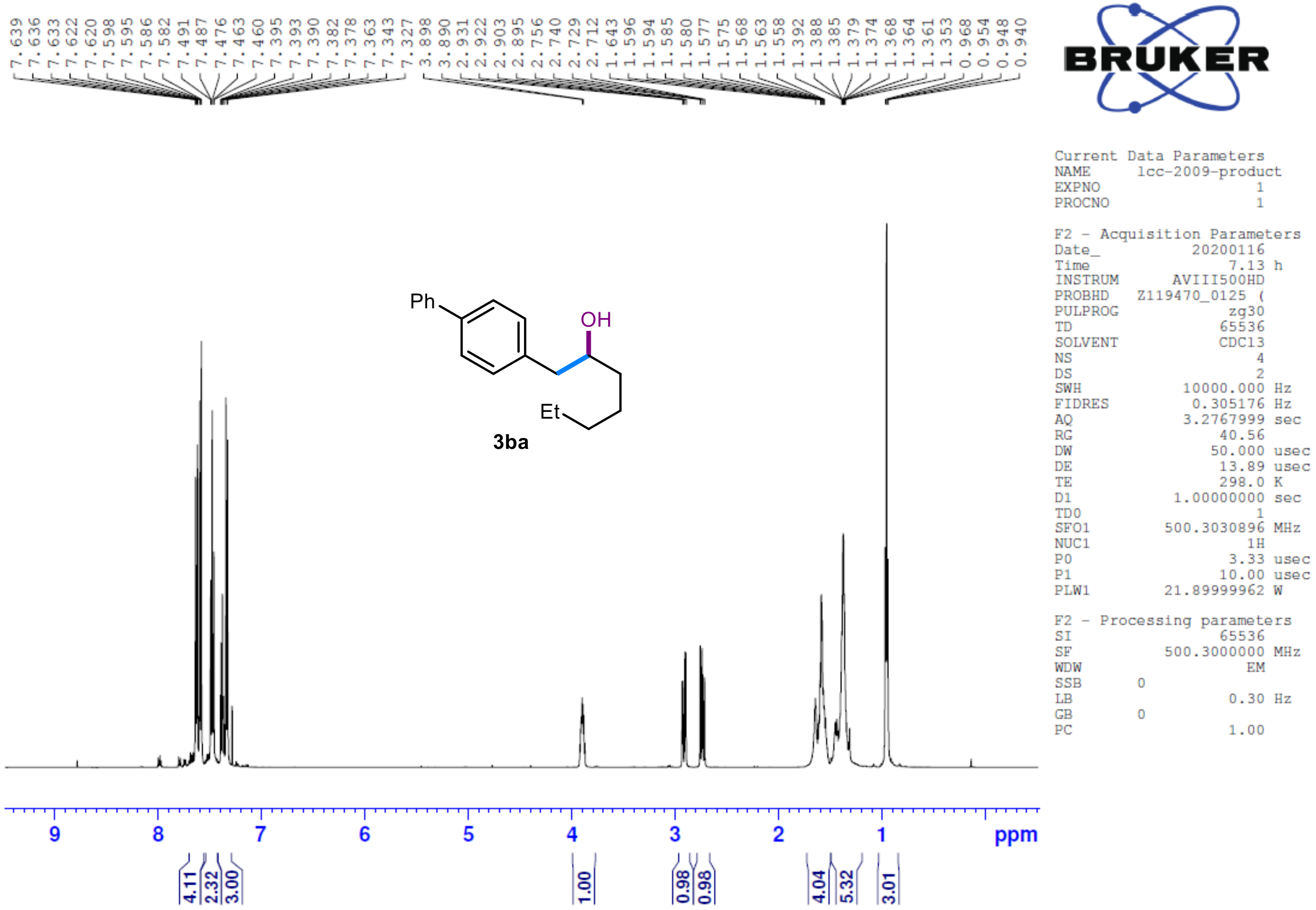
7.639 7.636 7.633 7.622 7.620 7.598 7.595 7.586 7.582 7.491 7.487 7.476 7.463 7.460 7.395 7.393 7.390 7.382 7.378 7.363 7.343 7.327 3.898 3.890 2.931 2.922 2.903 2.895 2.756 2.740 2.729 2.712 1.643 1.596 1.594 1.585 1.580 1.577 1.575 1.568 1.563 1.558 1.392 1.388 1.385 1.379 1.374 1.368 1.364 1.361 1.353 0.968 0.954 0.948 0.940
Ph
OH
Et
3ba
9 8 7 6 5 4 3 2 1 ppm
4.11 2.32 3.00 1.00 0.98 0.98 4.04 5.32 3.01
BRUKER
Current Data Parameters
NAME lcc-2009-product
EXPNO 1
PROCNO 1
F2 - Acquisition Parameters
Date_ 20200116
Time 7.13 h
INSTRUM AVIII500HD
PROBHD Z119470_0125 (
PULPROG zg30
TD 65536
SOLVENT CDCl3
NS 4
DS 2
SWH 10000.000 Hz
FIDRES 0.305176 Hz
AQ 3.2767999 sec
RG 40.56
DW 50.000 usec
DE 13.89 usec
TE 298.0 K
D1 1.00000000 sec
TD0 1
SFO1 500.3030896 MHz
NUC1 1H
P0 3.33 usec
P1 10.00 usec
PLW1 21.89999962 W
F2 - Processing parameters
SI 65536
SF 500.3000000 MHz
WDW EM
SSB 0
LB 0.30 Hz
GB 0
PC 1.00

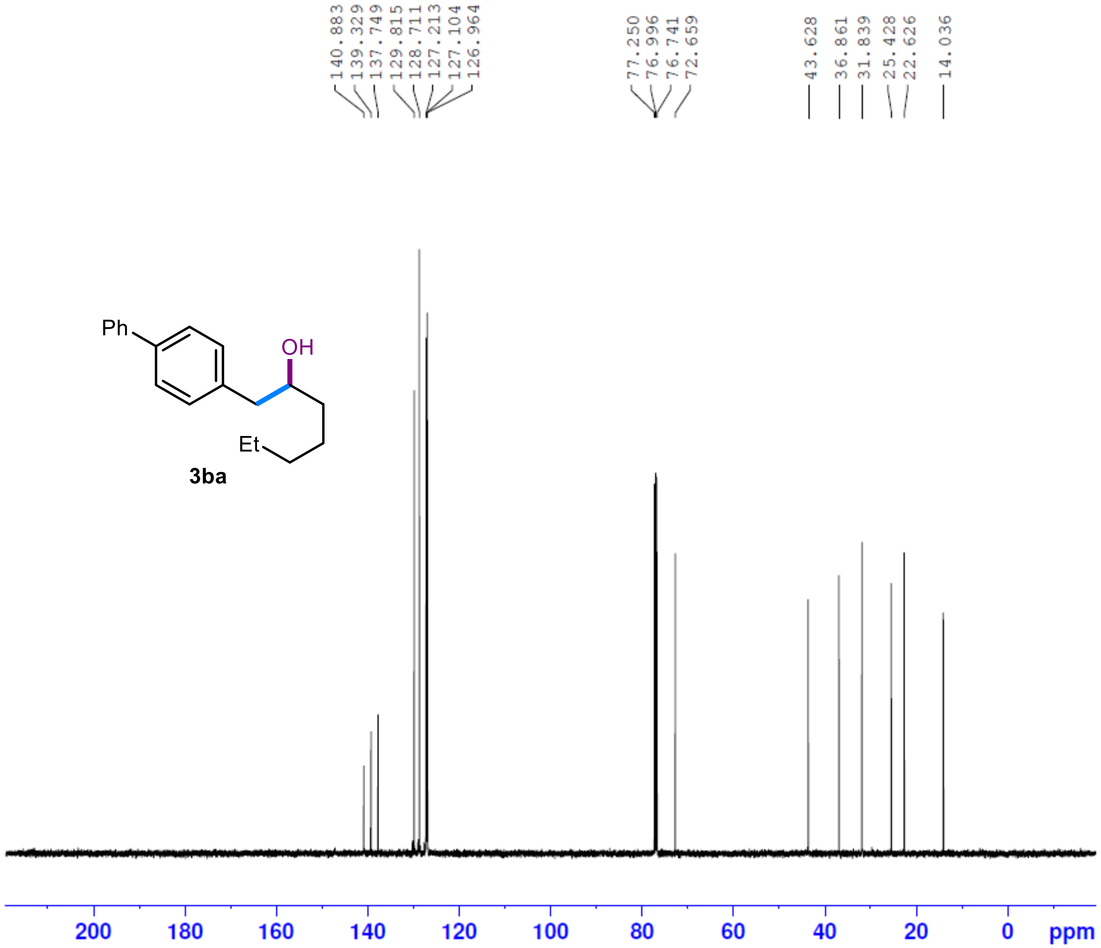

BRUKER

```
Current Data Parameters
NAME      lcc-2009-product
EXPNO                    2
PROCNO                   1

F2 - Acquisition Parameters
Date_           20200116
Time                7.20 h
INSTRUM       AVIII500HD
PROBHD    Z119470_0125 (
PULPROG          zgpg30
TD                65536
SOLVENT           CDCl3
NS                  128
DS                    4
SWH           30000.000 Hz
FIDRES         0.915527 Hz
AQ            1.0922667 sec
RG               192.72
DW               16.667 usec
DE                20.34 usec
TE                298.0 K
D1           2.00000000 sec
D11          0.03000000 sec
TD0                   1
SFO1        125.8131140 MHz
NUC1                13C
P0                 3.41 usec
P1                10.23 usec
PLW1        78.00000000 W
SFO2        500.3020012 MHz
NUC2                 1H
CPDPRG[2        waltz16
PCPD2             80.00 usec
PLW2        21.50000000 W
PLW12        0.57543999 W
PLW13        0.20446000 W

F2 - Processing parameters
SI                32768
SF          125.8005443 MHz
WDW                  EM
SSB       0
LB                 1.00 Hz
GB        0
PC                 1.40
```

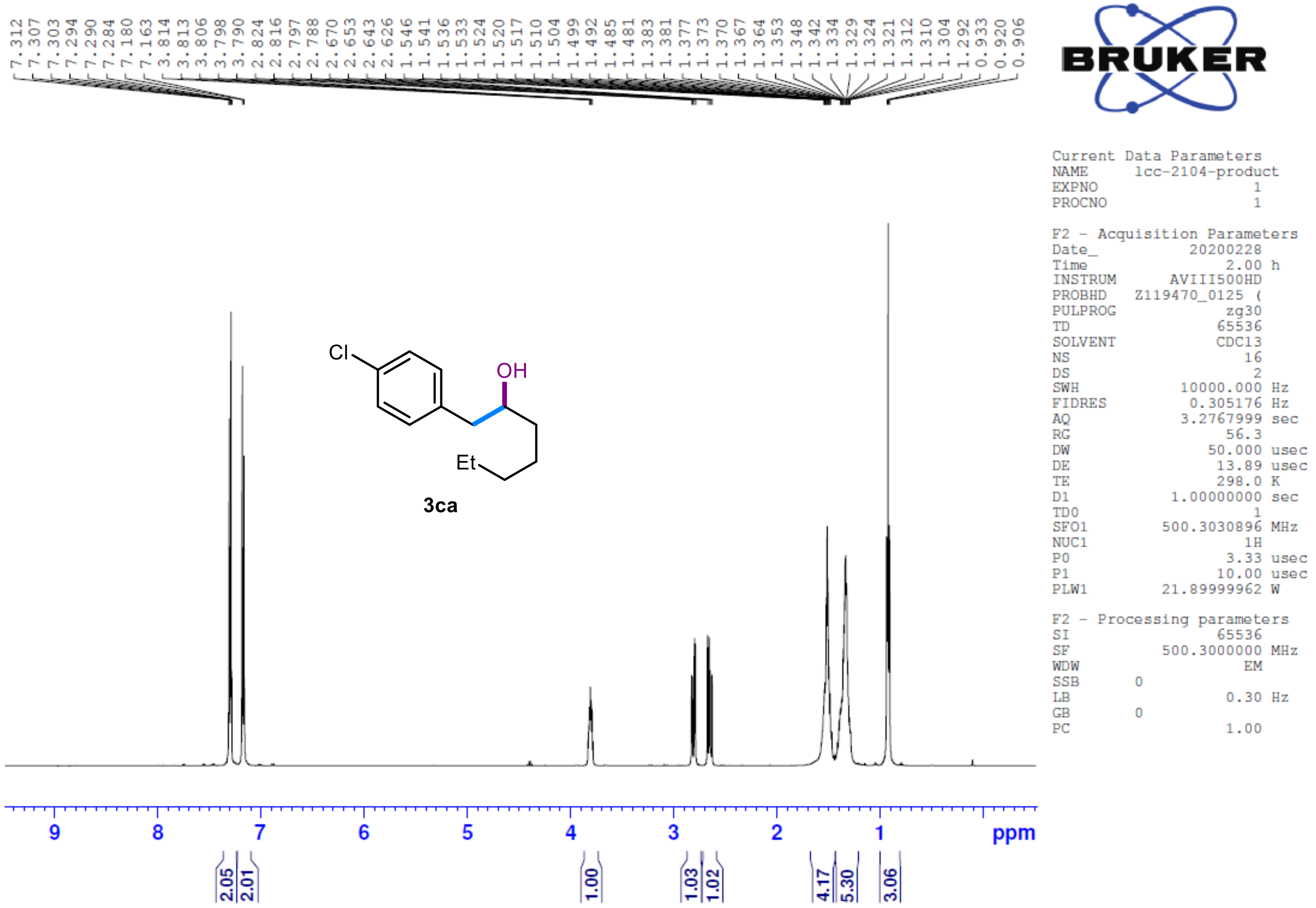
7.312
7.307
7.303
7.294
7.290
7.284
7.180
7.163
3.814
3.813
3.806
3.798
3.790
2.824
2.816
2.797
2.788
2.670
2.653
2.643
2.626
1.546
1.541
1.536
1.533
1.524
1.520
1.517
1.510
1.504
1.499
1.492
1.485
1.481
1.383
1.381
1.377
1.373
1.370
1.367
1.364
1.353
1.348
1.342
1.334
1.329
1.324
1.321
1.312
1.310
1.304
1.292
0.933
0.920
0.906
BRUKER
Current Data Parameters
NAME lcc-2104-product
EXPNO 1
PROCNO 1
F2 - Acquisition Parameters
Date_ 20200228
Time 2.00 h
INSTRUM AVIII500HD
PROBHD Z119470_0125 (
PULPROG zg30
TD 65536
SOLVENT CDCl3
NS 16
DS 2
SWH 10000.000 Hz
FIDRES 0.305176 Hz
AQ 3.2767999 sec
RG 56.3
DW 50.000 usec
DE 13.89 usec
TE 298.0 K
D1 1.00000000 sec
TD0 1
SFO1 500.3030896 MHz
NUC1 1H
P0 3.33 usec
P1 10.00 usec
PLW1 21.89999962 W
F2 - Processing parameters
SI 65536
SF 500.3000000 MHz
WDW EM
SSB 0
LB 0.30 Hz
GB 0
PC 1.00
Cl
OH
Et
3ca
9
8
7
6
5
4
3
2
1
ppm
2.05
2.01
1.00
1.03
1.02
4.17
5.30
3.06

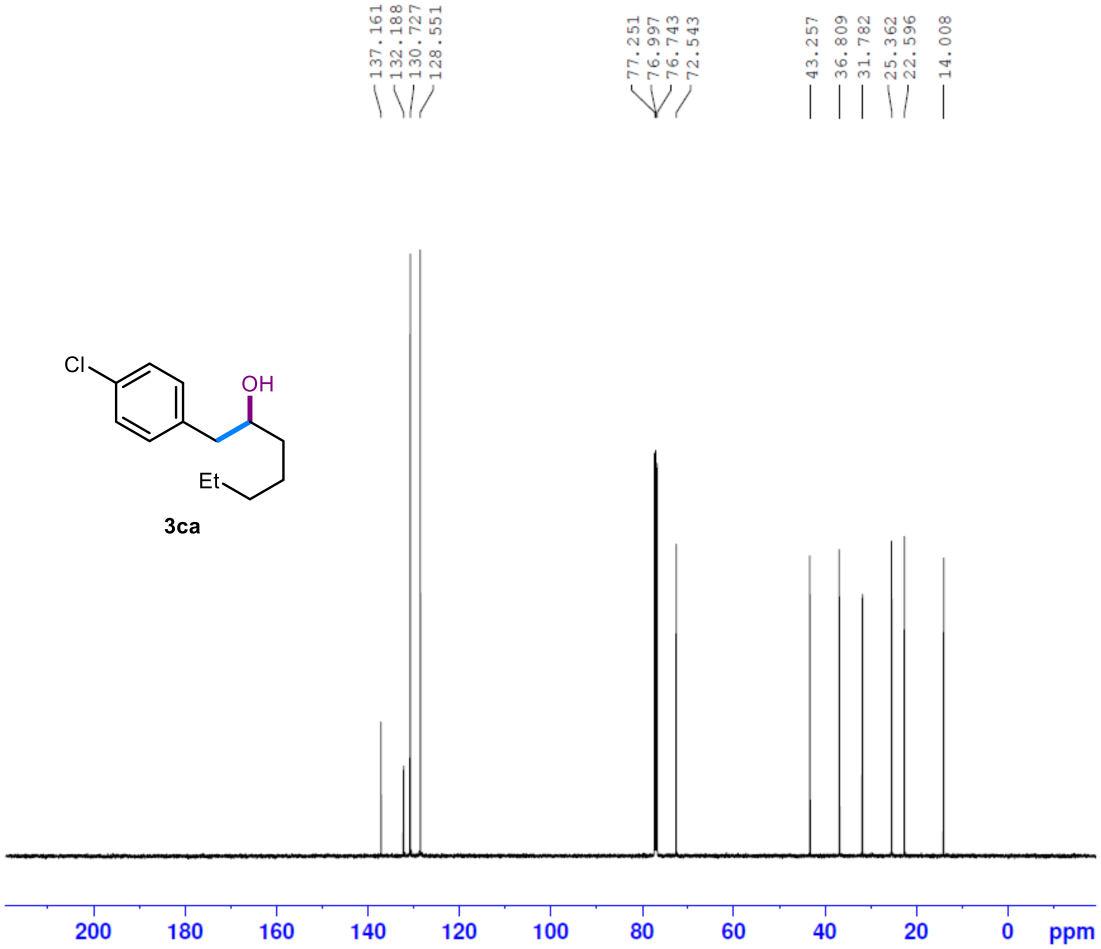

Current Data Parameters

NAME	lcc-2104-product
EXPNO	2
PROCNO	1

F2 - Acquisition Parameters

Date_	20200228
Time	2.28 h
INSTRUM	AVIII500HD
PROBHD	Z119470_0125 (
PULPROG	zgpg30
TD	65536
SOLVENT	CDCl3
NS	512
DS	4
SWH	30000.000 Hz
FIDRES	0.915527 Hz
AQ	1.0922667 sec
RG	192.72
DW	16.667 usec
DE	20.34 usec
TE	298.0 K
D1	2.00000000 sec
D11	0.03000000 sec
TD0	1
SFO1	125.8131140 MHz
NUC1	13C
P0	3.41 usec
P1	10.23 usec
PLW1	78.00000000 W
SFO2	500.3020012 MHz
NUC2	1H
CPDPRG[2	waltz16
PCPD2	80.00 usec
PLW2	21.50000000 W
PLW12	0.57543999 W
PLW13	0.20446000 W

F2 - Processing parameters

SI	32768
SF	125.8005407 MHz
WDW	EM
SSB	0
LB	1.00 Hz
GB	0
PC	1.40

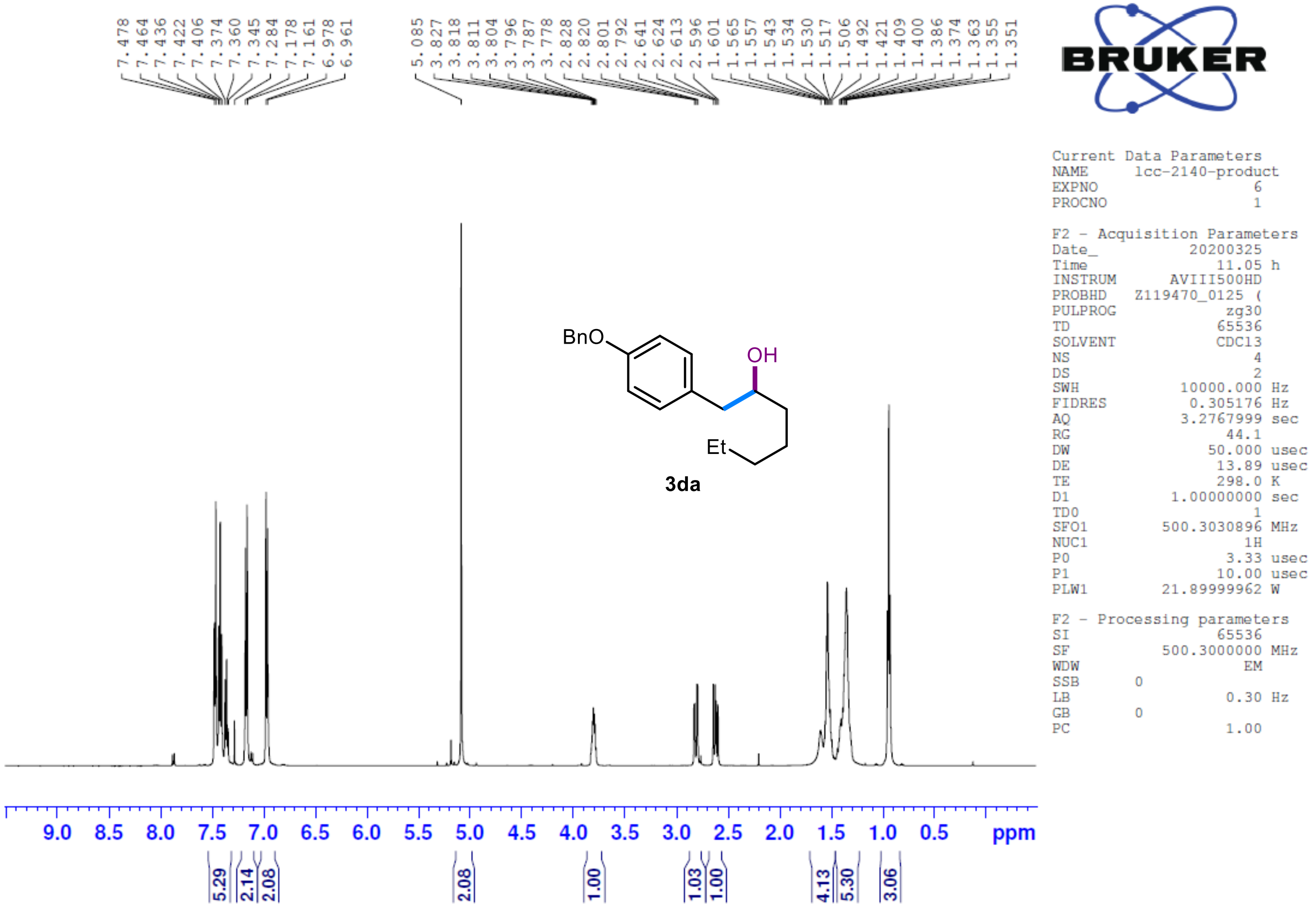
BnO
OH
Et
3da
Current Data Parameters
NAME lcc-2140-product
EXPNO 6
PROCNO 1
F2 - Acquisition Parameters
Date_ 20200325
Time 11.05 h
INSTRUM AVIII500HD
PROBHD Z119470_0125 (
PULPROG zg30
TD 65536
SOLVENT CDCl3
NS 4
DS 2
SWH 10000.000 Hz
FIDRES 0.305176 Hz
AQ 3.2767999 sec
RG 44.1
DW 50.000 usec
DE 13.89 usec
TE 298.0 K
D1 1.00000000 sec
TD0 1
SFO1 500.3030896 MHz
NUC1 1H
P0 3.33 usec
P1 10.00 usec
PLW1 21.89999962 W
F2 - Processing parameters
SI 65536
SF 500.3000000 MHz
WDW EM
SSB 0
LB 0.30 Hz
GB 0
PC 1.00
ppm

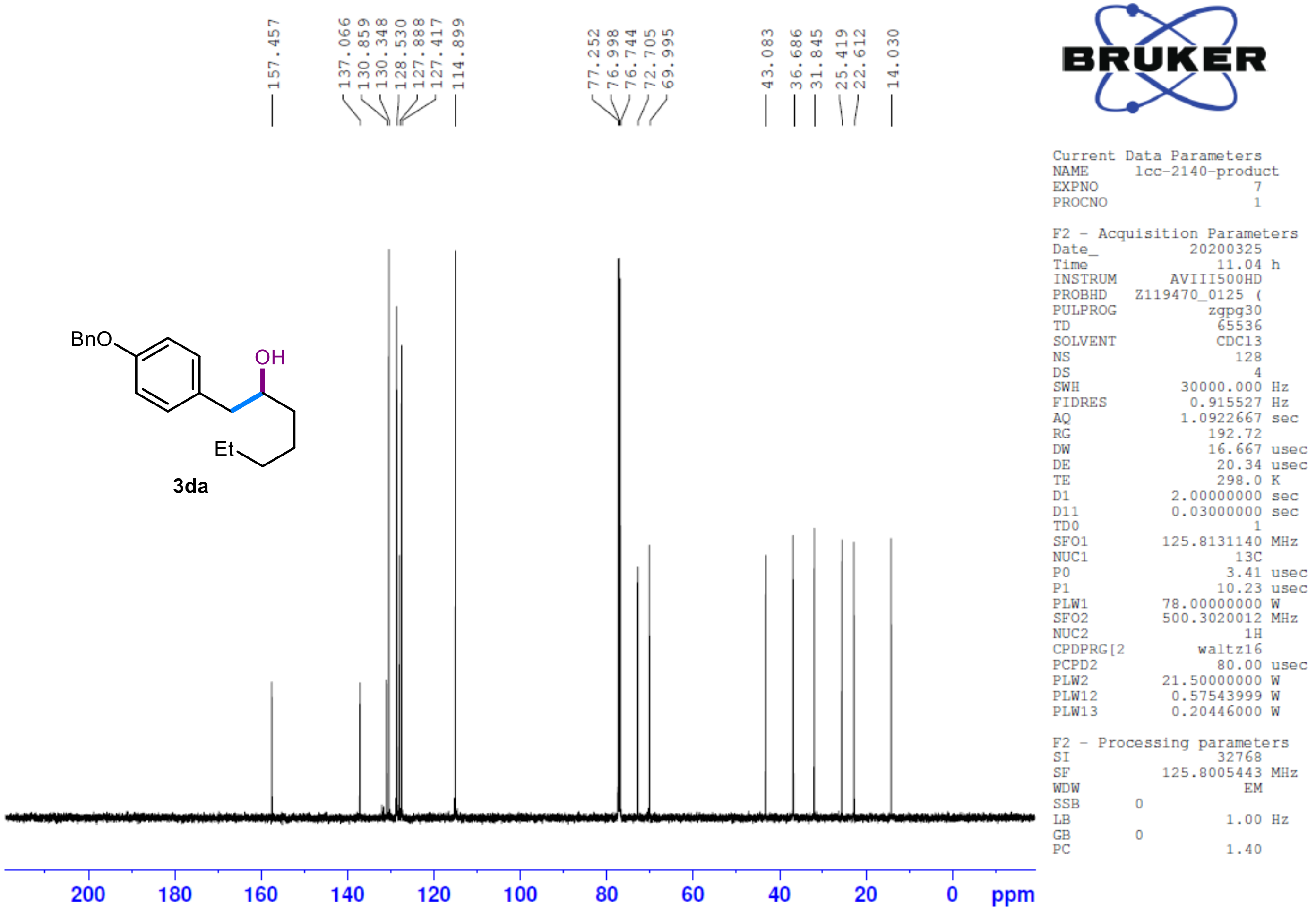
157.457
137.066
130.859
130.348
128.530
127.888
127.417
114.899
77.252
76.998
76.744
72.705
69.995
43.083
36.686
31.845
25.419
22.612
14.030
BnO
OH
Et
3da
BRUKER
Current Data Parameters
NAME lcc-2140-product
EXPNO 7
PROCNO 1
F2 - Acquisition Parameters
Date_ 20200325
Time 11.04 h
INSTRUM AVIII500HD
PROBHD Z119470_0125 (
PULPROG zgpg30
TD 65536
SOLVENT CDCl3
NS 128
DS 4
SWH 30000.000 Hz
FIDRES 0.915527 Hz
AQ 1.0922667 sec
RG 192.72
DW 16.667 usec
DE 20.34 usec
TE 298.0 K
D1 2.00000000 sec
D11 0.03000000 sec
TD0 1
SFO1 125.8131140 MHz
NUC1 13C
P0 3.41 usec
P1 10.23 usec
PLW1 78.00000000 W
SFO2 500.3020012 MHz
NUC2 1H
CPDPRG[2 waltz16
PCPD2 80.00 usec
PLW2 21.50000000 W
PLW12 0.57543999 W
PLW13 0.20446000 W
F2 - Processing parameters
SI 32768
SF 125.8005443 MHz
WDW EM
SSB 0
LB 1.00 Hz
GB 0
PC 1.40
200
180
160
140
120
100
80
60
40
20
0
ppm

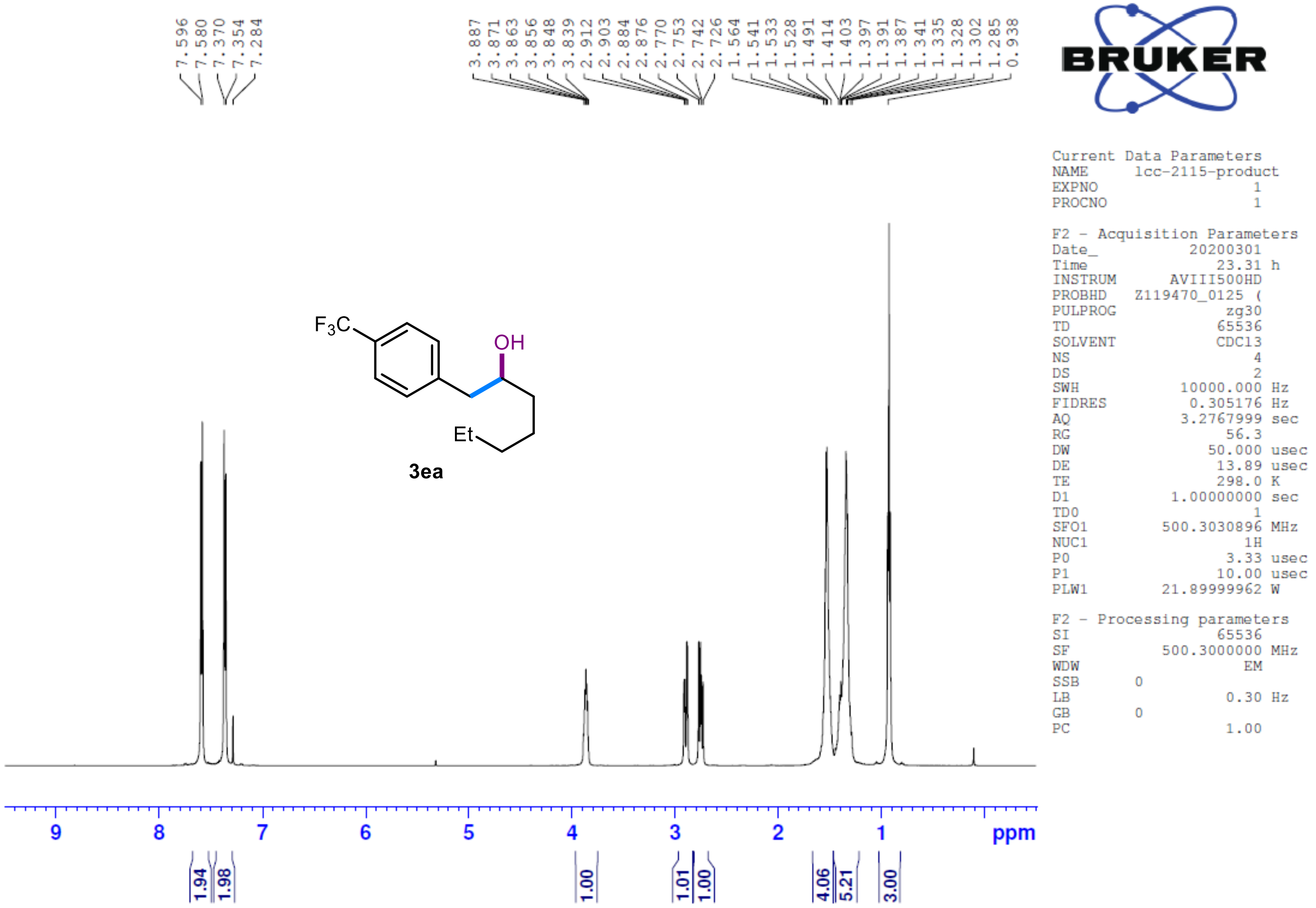
7.596
7.580
7.370
7.354
7.284
3.887
3.871
3.863
3.856
3.848
3.839
2.912
2.903
2.884
2.876
2.770
2.753
2.742
2.726
1.564
1.541
1.533
1.528
1.491
1.414
1.403
1.397
1.391
1.387
1.341
1.335
1.328
1.302
1.285
0.938
BRUKER
F3C
OH
Et
3ea
Current Data Parameters
NAME lcc-2115-product
EXPNO 1
PROCNO 1
F2 - Acquisition Parameters
Date_ 20200301
Time 23.31 h
INSTRUM AVIII500HD
PROBHD Z119470_0125 (
PULPROG zg30
TD 65536
SOLVENT CDCl3
NS 4
DS 2
SWH 10000.000 Hz
FIDRES 0.305176 Hz
AQ 3.2767999 sec
RG 56.3
DW 50.000 usec
DE 13.89 usec
TE 298.0 K
D1 1.00000000 sec
TD0 1
SFO1 500.3030896 MHz
NUC1 1H
P0 3.33 usec
P1 10.00 usec
PLW1 21.89999962 W
F2 - Processing parameters
SI 65536
SF 500.3000000 MHz
WDW EM
SSB 0
LB 0.30 Hz
GB 0
PC 1.00
9
8
7
6
5
4
3
2
1
ppm
1.94
1.98
1.00
1.01
1.00
4.06
5.21
3.00

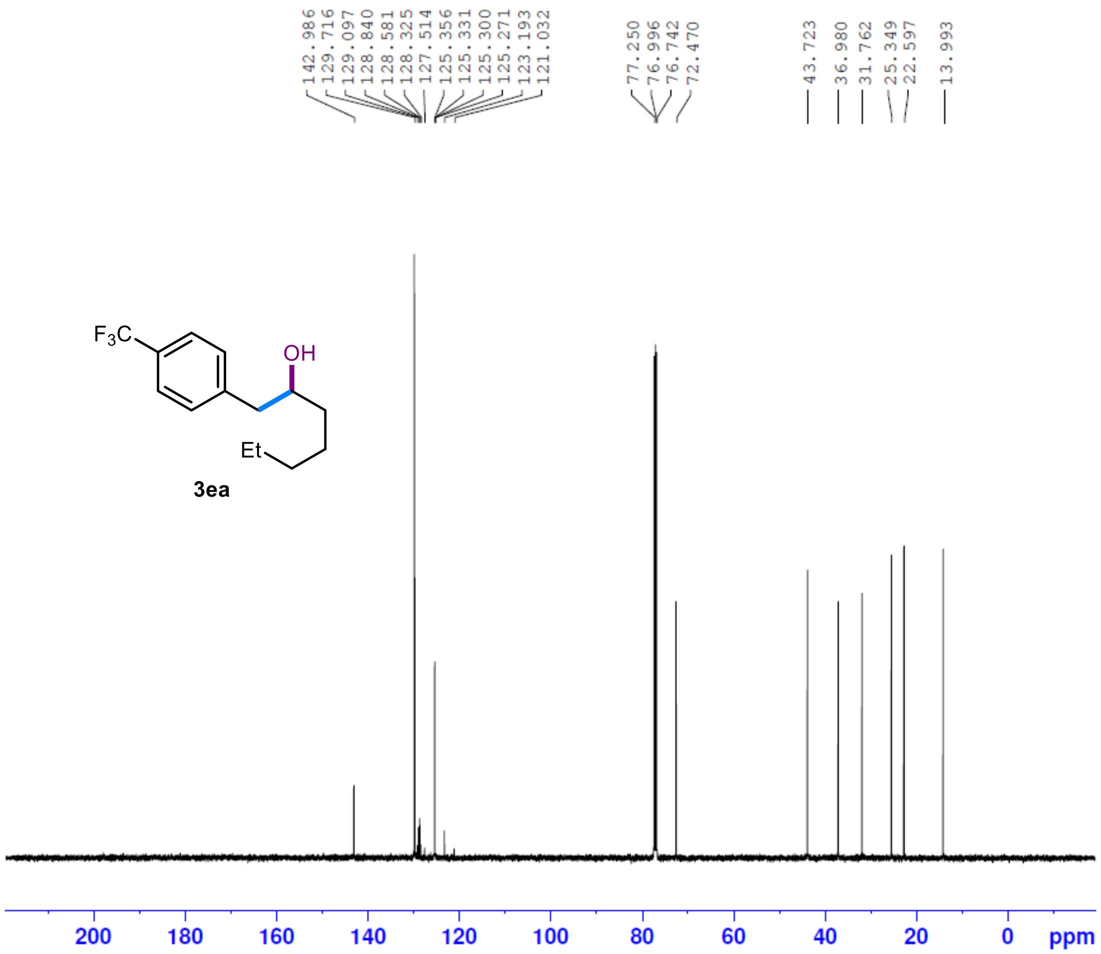

Current Data Parameters
NAME lcc-2115-product
EXPNO 3
PROCNO 1

F2 - Acquisition Parameters
Date_ 20200301
Time 23.47 h
INSTRUM AVIII500HD
PROBHD Z119470_0125 (
PULPROG zgpg30
TD 65536
SOLVENT CDCl3
NS 256
DS 4
SWH 30000.000 Hz
FIDRES 0.915527 Hz
AQ 1.0922667 sec
RG 192.72
DW 16.667 usec
DE 20.34 usec
TE 298.0 K
D1 2.00000000 sec
D11 0.03000000 sec
TD0 1
SFO1 125.8131140 MHz
NUC1 13C
P0 3.41 usec
P1 10.23 usec
PLW1 78.00000000 W
SFO2 500.3020012 MHz
NUC2 1H
CPDPRG[2 waltz16
PCPD2 80.00 usec
PLW2 21.50000000 W
PLW12 0.57543999 W
PLW13 0.20446000 W

F2 - Processing parameters
SI 32768
SF 125.8005388 MHz
WDW EM
SSB 0
LB 1.00 Hz
GB 0
PC 1.40

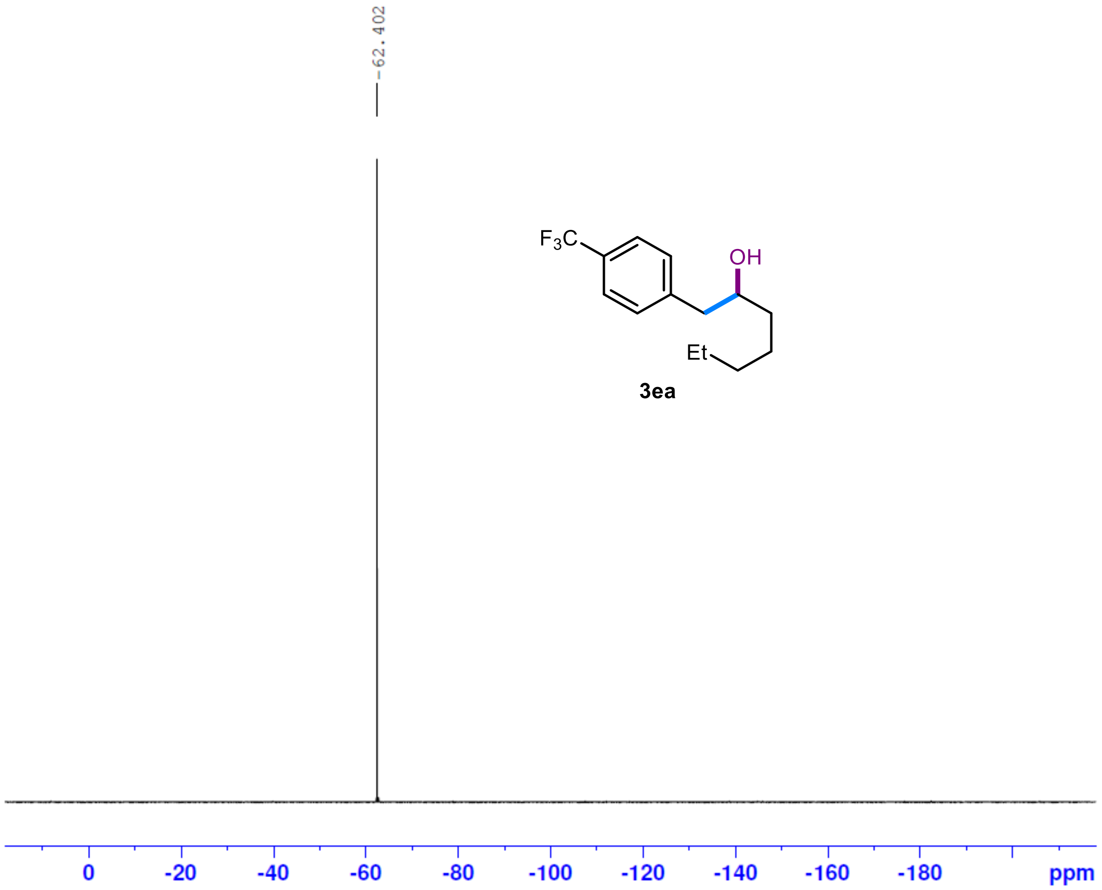

Current Data Parameters

NAME	lcc-2115-product
EXPNO	2
PROCNO	1

F2 - Acquisition Parameters

Date_	20200301
Time	23.32 h
INSTRUM	AVIII500HD
PROBHD	Z119470_0125 (
PULPROG	zgbsfhigqn.baseopt
TD	131072
SOLVENT	CDCl3
NS	16
DS	4
SWH	111111.109 Hz
FIDRES	1.695421 Hz
AQ	0.5898240 sec
RG	192.72
DW	4.500 usec
DE	35.33 usec
TE	298.0 K
D1	1.00000000 sec
D11	0.03000000 sec
D12	0.00002000 sec
TD0	1
SFO1	470.7052618 MHz
NUC1	19F
P1	15.00 usec
P2	30.00 usec
PLW1	39.50000000 W
SFO2	500.3025015 MHz
NUC2	1H
CPDPRG[2	waltz16
PCPD2	80.00 usec
PLW2	21.50000000 W
PLW12	0.57543999 W

F2 - Processing parameters

SI	65536
SF	470.7523370 MHz
WDW	EM
SSB	0
LB	0.30 Hz
GB	0
PC	1.00

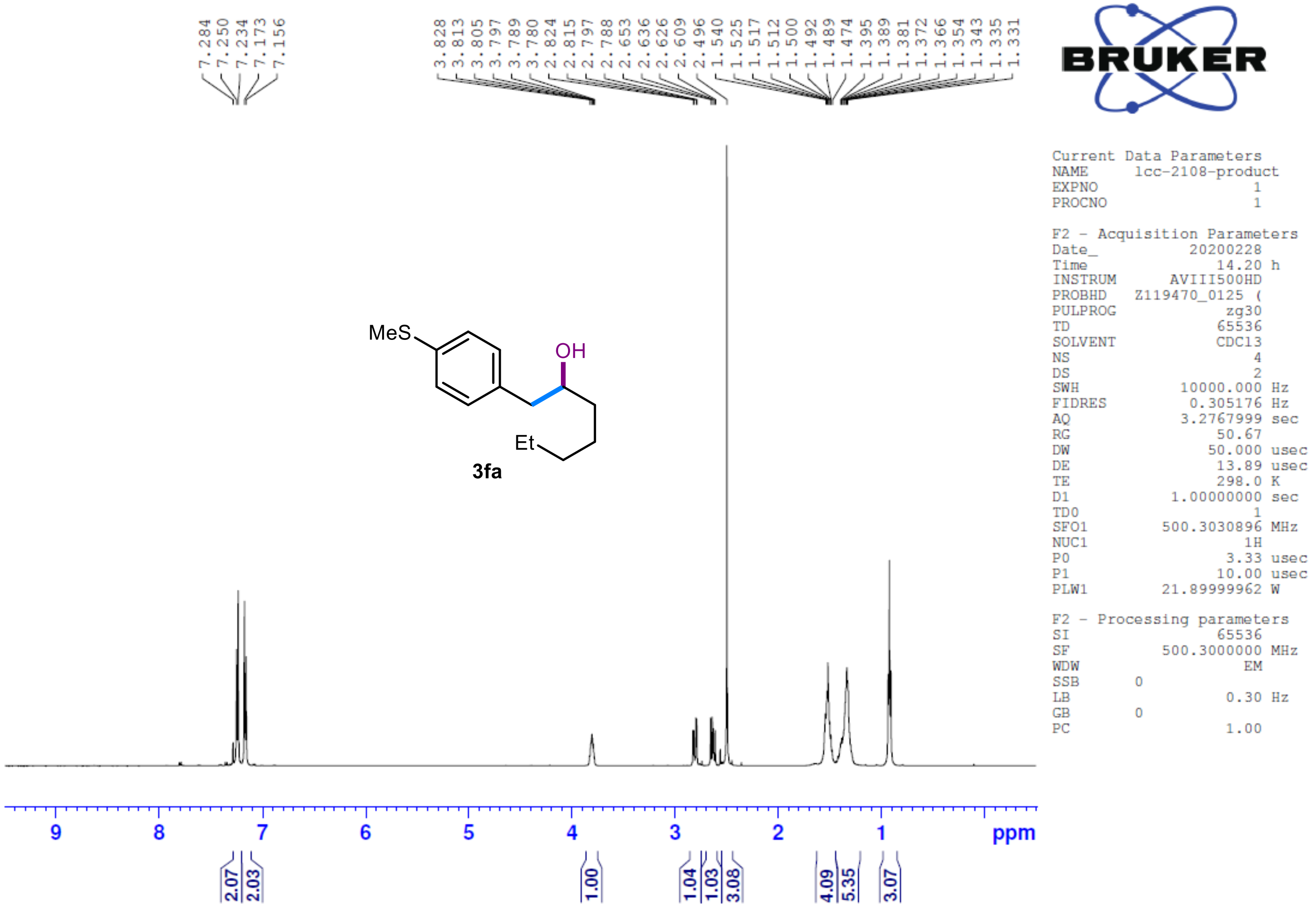

7.284
7.250
7.234
7.173
7.156
3.828
3.813
3.805
3.797
3.789
3.780
2.824
2.815
2.797
2.788
2.653
2.636
2.626
2.609
2.496
1.540
1.525
1.517
1.512
1.500
1.492
1.489
1.474
1.395
1.389
1.381
1.372
1.366
1.354
1.343
1.335
1.331
BRUKER
MeS
OH
Et
3fa
Current Data Parameters
NAME lcc-2108-product
EXPNO 1
PROCNO 1
F2 - Acquisition Parameters
Date_ 20200228
Time 14.20 h
INSTRUM AVIII500HD
PROBHD Z119470_0125 (
PULPROG zg30
TD 65536
SOLVENT CDCl3
NS 4
DS 2
SWH 10000.000 Hz
FIDRES 0.305176 Hz
AQ 3.2767999 sec
RG 50.67
DW 50.000 usec
DE 13.89 usec
TE 298.0 K
D1 1.00000000 sec
TD0 1
SFO1 500.3030896 MHz
NUC1 1H
P0 3.33 usec
P1 10.00 usec
PLW1 21.89999962 W
F2 - Processing parameters
SI 65536
SF 500.3000000 MHz
WDW EM
SSB 0
LB 0.30 Hz
GB 0
PC 1.00
9
8
7
6
5
4
3
2
1
ppm
2.07
2.03
1.00
1.04
1.03
3.08
4.09
5.35
3.07

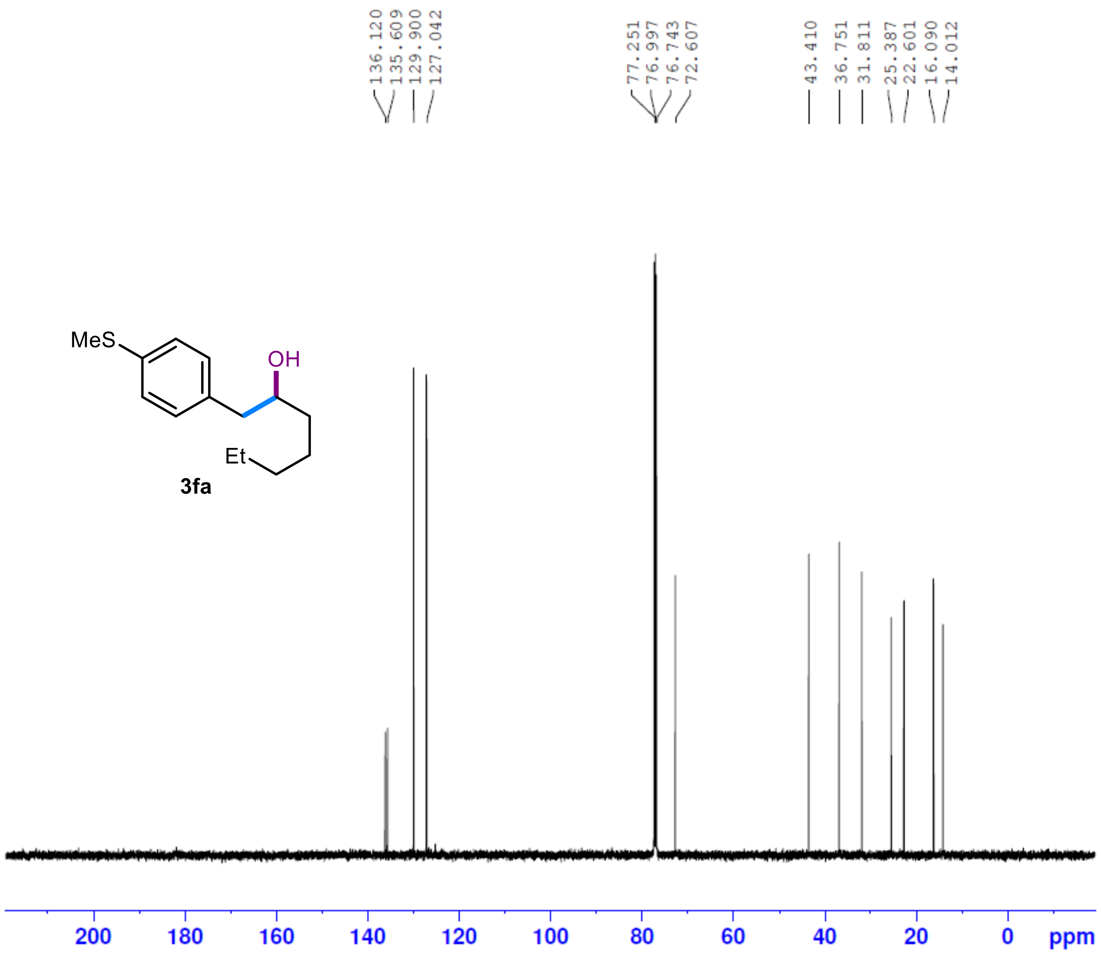

Current Data Parameters
NAME lcc-2108-product
EXPNO 2
PROCNO 1

F2 - Acquisition Parameters
Date_ 20200228
Time 14.27 h
INSTRUM AVIII500HD
PROBHD Z119470_0125 (
PULPROG zgpg30
TD 65536
SOLVENT CDCl3
NS 128
DS 4
SWH 30000.000 Hz
FIDRES 0.915527 Hz
AQ 1.0922667 sec
RG 192.72
DW 16.667 usec
DE 20.34 usec
TE 298.0 K
D1 2.00000000 sec
D11 0.03000000 sec
TD0 1
SFO1 125.8131140 MHz
NUC1 13C
P0 3.41 usec
P1 10.23 usec
PLW1 78.00000000 W
SFO2 500.3020012 MHz
NUC2 1H
CPDPRG[2 waltz16
PCPD2 80.00 usec
PLW2 21.50000000 W
PLW12 0.57543999 W
PLW13 0.20446000 W

F2 - Processing parameters
SI 32768
SF 125.8005425 MHz
WDW EM
SSB 0
LB 1.00 Hz
GB 0
PC 1.40

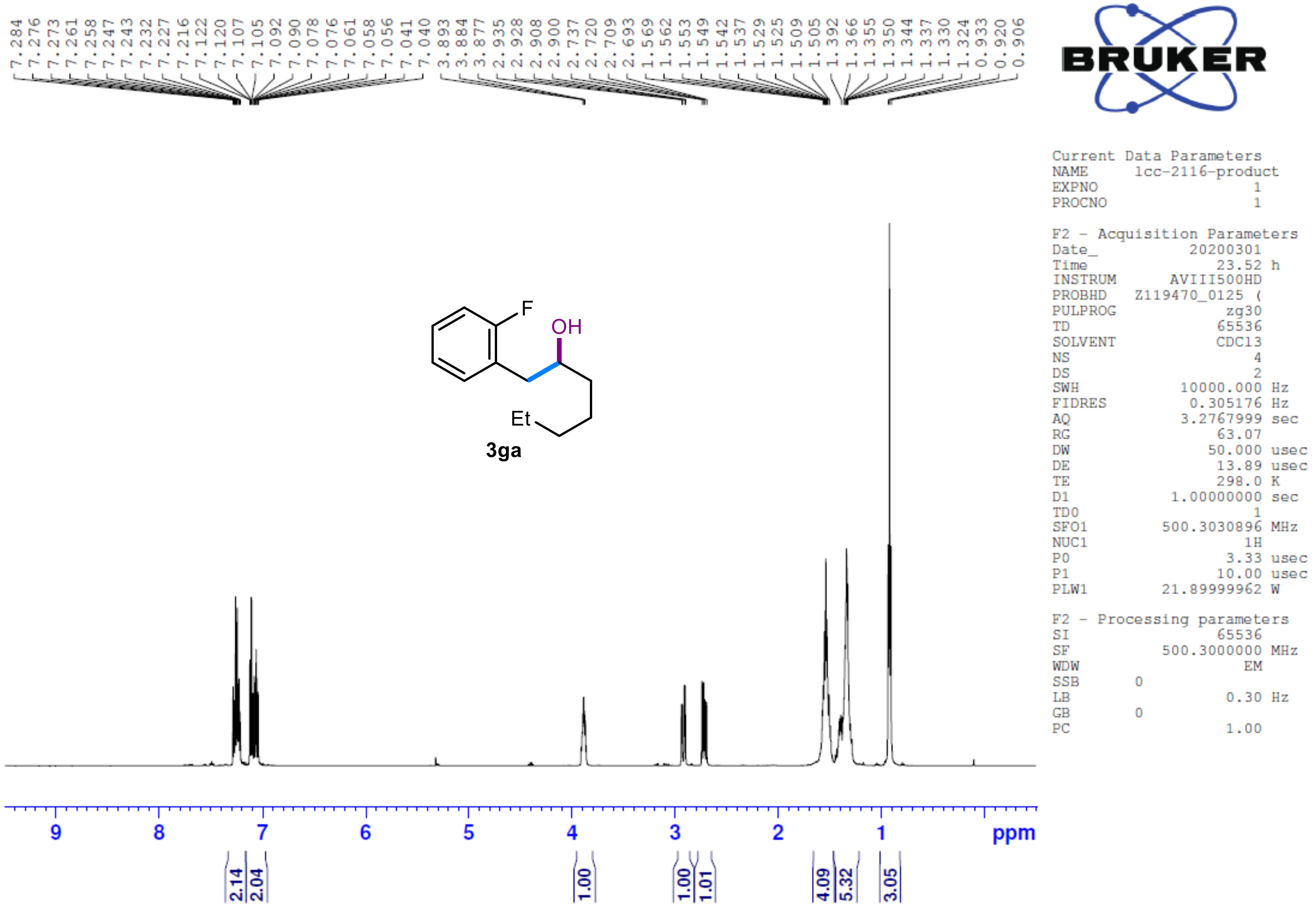
7.284 7.276 7.273 7.261 7.258 7.247 7.243 7.232 7.227 7.216 7.122 7.120 7.107 7.105 7.092 7.090 7.078 7.076 7.061 7.058 7.056 7.041 7.040 3.893 3.884 3.877 2.935 2.928 2.908 2.900 2.737 2.720 2.709 2.693 1.569 1.562 1.553 1.549 1.542 1.537 1.529 1.525 1.509 1.505 1.392 1.366 1.355 1.350 1.344 1.337 1.330 1.324 0.933 0.920 0.906
BRUKER
F
OH
Et
3ga
Current Data Parameters
NAME lcc-2116-product
EXPNO 1
PROCNO 1
F2 - Acquisition Parameters
Date_ 20200301
Time 23.52 h
INSTRUM AVIII500HD
PROBHD Z119470_0125 (
PULPROG zg30
TD 65536
SOLVENT CDCl3
NS 4
DS 2
SWH 10000.000 Hz
FIDRES 0.305176 Hz
AQ 3.2767999 sec
RG 63.07
DW 50.000 usec
DE 13.89 usec
TE 298.0 K
D1 1.00000000 sec
TD0 1
SFO1 500.3030896 MHz
NUC1 1H
P0 3.33 usec
P1 10.00 usec
PLW1 21.89999962 W
F2 - Processing parameters
SI 65536
SF 500.3000000 MHz
WDW EM
SSB 0
LB 0.30 Hz
GB 0
PC 1.00
9 8 7 6 5 4 3 2 1 ppm
2.14 2.04 1.00 1.00 1.01 4.09 5.32 3.05

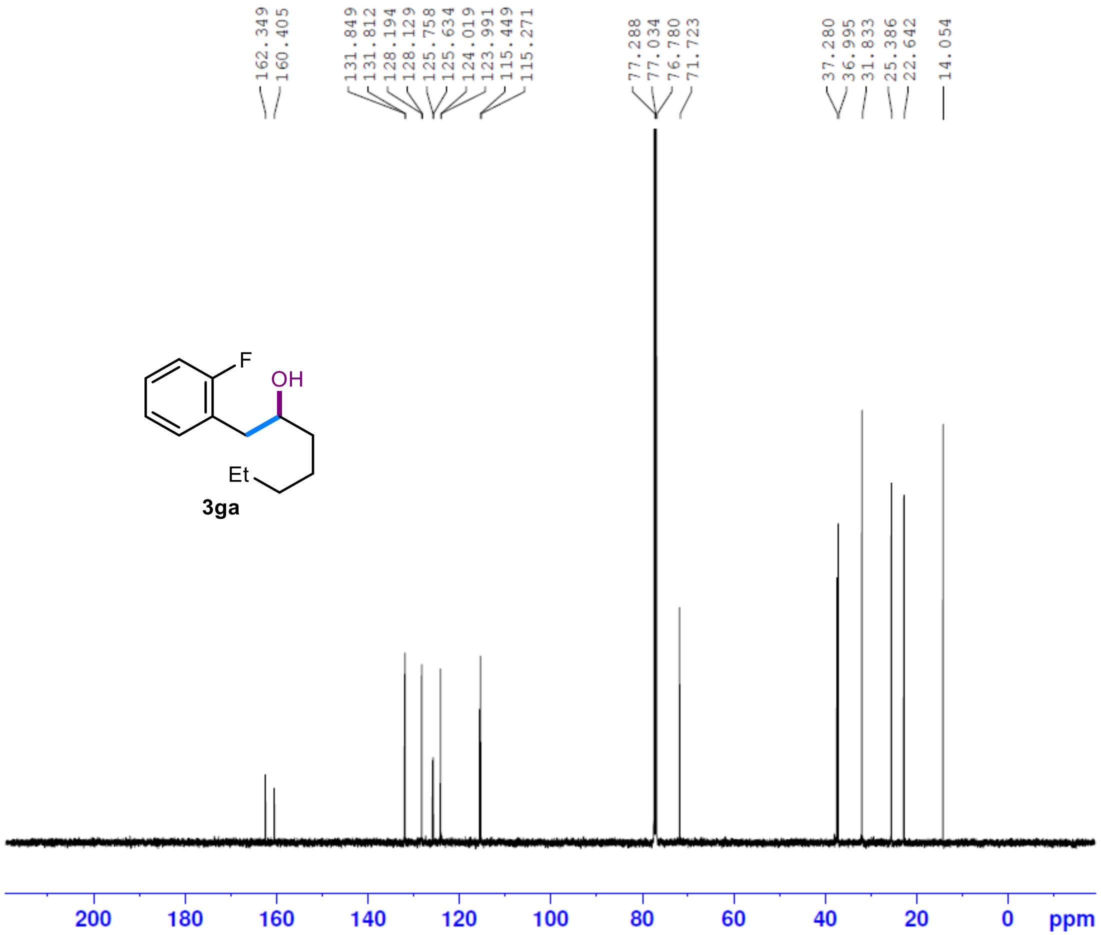

BRUKER

```
Current Data Parameters
NAME       lcc-2116-product
EXPNO                     3
PROCNO                    1

F2 - Acquisition Parameters
Date_              20200302
Time                 0.08 h
INSTRUM          AVIII500HD
PROBHD     Z119470_0125 (
PULPROG              zgpg30
TD                    65536
SOLVENT               CDCl3
NS                      256
DS                        4
SWH           30000.000 Hz
FIDRES         0.915527 Hz
AQ            1.0922667 sec
RG                   192.72
DW               16.667 usec
DE                20.34 usec
TE                298.0 K
D1           2.00000000 sec
D11          0.03000000 sec
TD0                       1
SFO1        125.8131140 MHz
NUC1                    13C
P0                 3.41 usec
P1                10.23 usec
PLW1        78.00000000 W
SFO2        500.3020012 MHz
NUC2                     1H
CPDPRG[2            waltz16
PCPD2             80.00 usec
PLW2        21.50000000 W
PLW12        0.57543999 W
PLW13        0.20446000 W

F2 - Processing parameters
SI                    32768
SF          125.8005351 MHz
WDW                      EM
SSB      0
LB                   1.00 Hz
GB       0
PC                     1.40
```

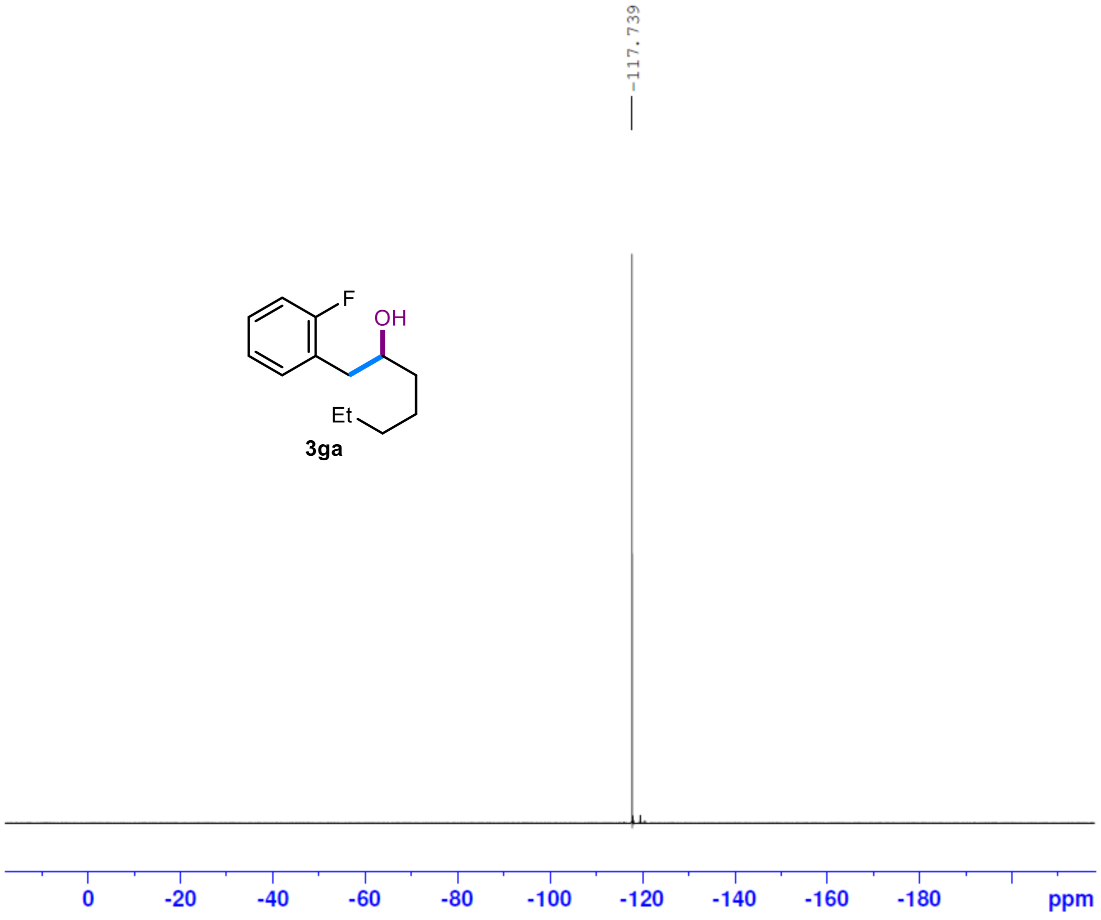

Current Data Parameters
NAME lcc-2116-product
EXPNO 2
PROCNO 1

F2 - Acquisition Parameters
Date_ 20200301
Time 23.53 h
INSTRUM AVIII500HD
PROBHD Z119470_0125 (
PULPROG zgbsfhigqn.baseopt
TD 131072
SOLVENT CDCl3
NS 16
DS 4
SWH 111111.109 Hz
FIDRES 1.695421 Hz
AQ 0.5898240 sec
RG 192.72
DW 4.500 usec
DE 35.33 usec
TE 298.0 K
D1 1.00000000 sec
D11 0.03000000 sec
D12 0.00002000 sec
TD0 1
SFO1 470.7052618 MHz
NUC1 19F
P1 15.00 usec
P2 30.00 usec
PLW1 39.50000000 W
SFO2 500.3025015 MHz
NUC2 1H
CPDPRG[2 waltz16
PCPD2 80.00 usec
PLW2 21.50000000 W
PLW12 0.57543999 W

F2 - Processing parameters
SI 65536
SF 470.7523370 MHz
WDW EM
SSB 0
LB 0.30 Hz
GB 0
PC 1.00

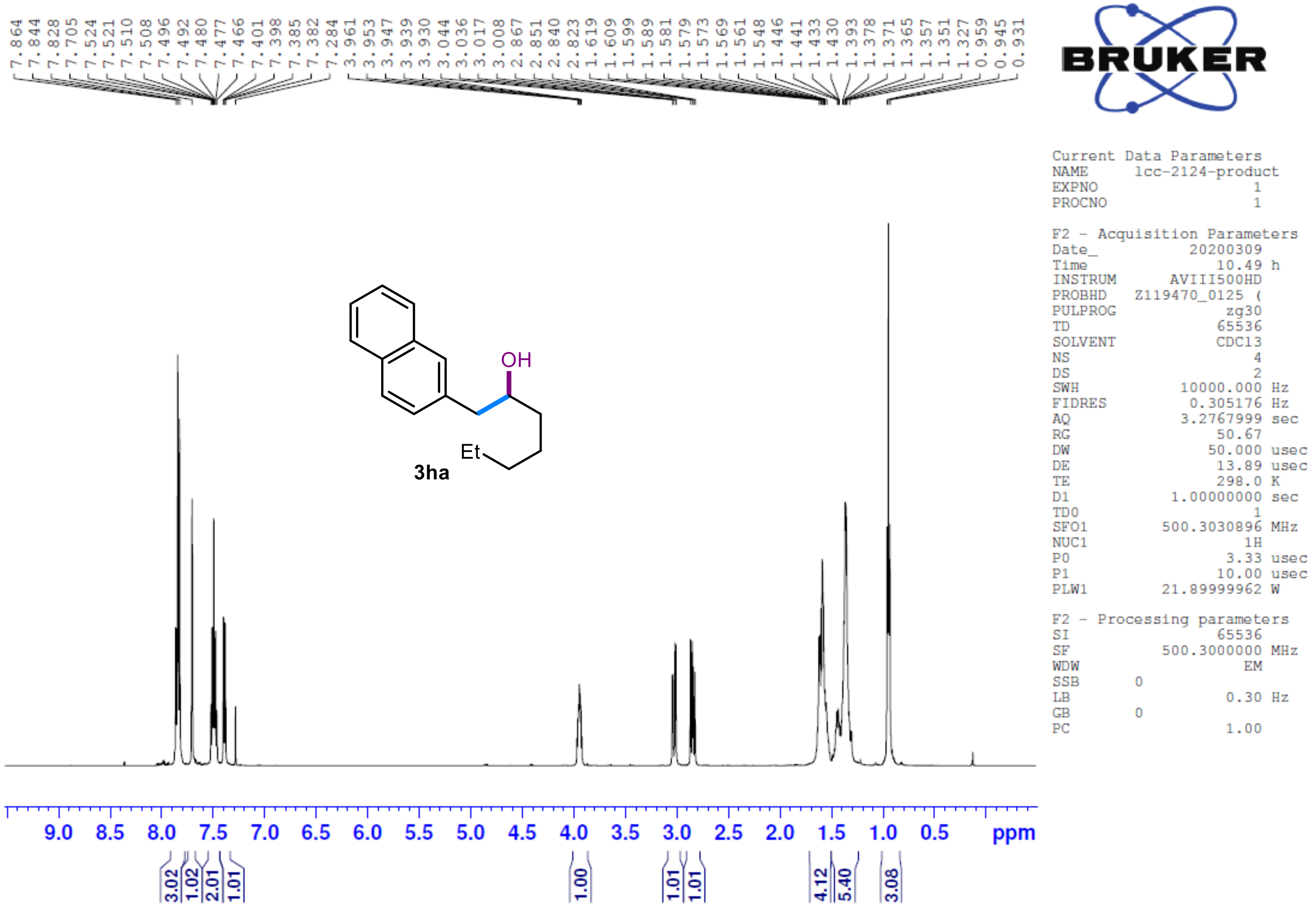

7.864
7.844
7.828
7.705
7.524
7.521
7.510
7.508
7.496
7.492
7.480
7.477
7.466
7.401
7.398
7.385
7.382
7.284
3.961
3.953
3.947
3.939
3.930
3.044
3.036
3.017
3.008
2.867
2.851
2.840
2.823
1.619
1.609
1.599
1.589
1.581
1.579
1.573
1.569
1.561
1.548
1.446
1.441
1.433
1.430
1.393
1.378
1.371
1.365
1.357
1.351
1.327
0.959
0.945
0.931
BRUKER
OH
Et
3ha
Current Data Parameters
NAME lcc-2124-product
EXPNO 1
PROCNO 1
F2 - Acquisition Parameters
Date_ 20200309
Time 10.49 h
INSTRUM AVIII500HD
PROBHD Z119470_0125 (
PULPROG zg30
TD 65536
SOLVENT CDCl3
NS 4
DS 2
SWH 10000.000 Hz
FIDRES 0.305176 Hz
AQ 3.2767999 sec
RG 50.67
DW 50.000 usec
DE 13.89 usec
TE 298.0 K
D1 1.00000000 sec
TD0 1
SFO1 500.3030896 MHz
NUC1 1H
P0 3.33 usec
P1 10.00 usec
PLW1 21.89999962 W
F2 - Processing parameters
SI 65536
SF 500.3000000 MHz
WDW EM
SSB 0
LB 0.30 Hz
GB 0
PC 1.00
9.0 8.5 8.0 7.5 7.0 6.5 6.0 5.5 5.0 4.5 4.0 3.5 3.0 2.5 2.0 1.5 1.0 0.5 ppm
3.02
1.02
2.01
1.01
1.00
1.01
1.01
4.12
5.40
3.08

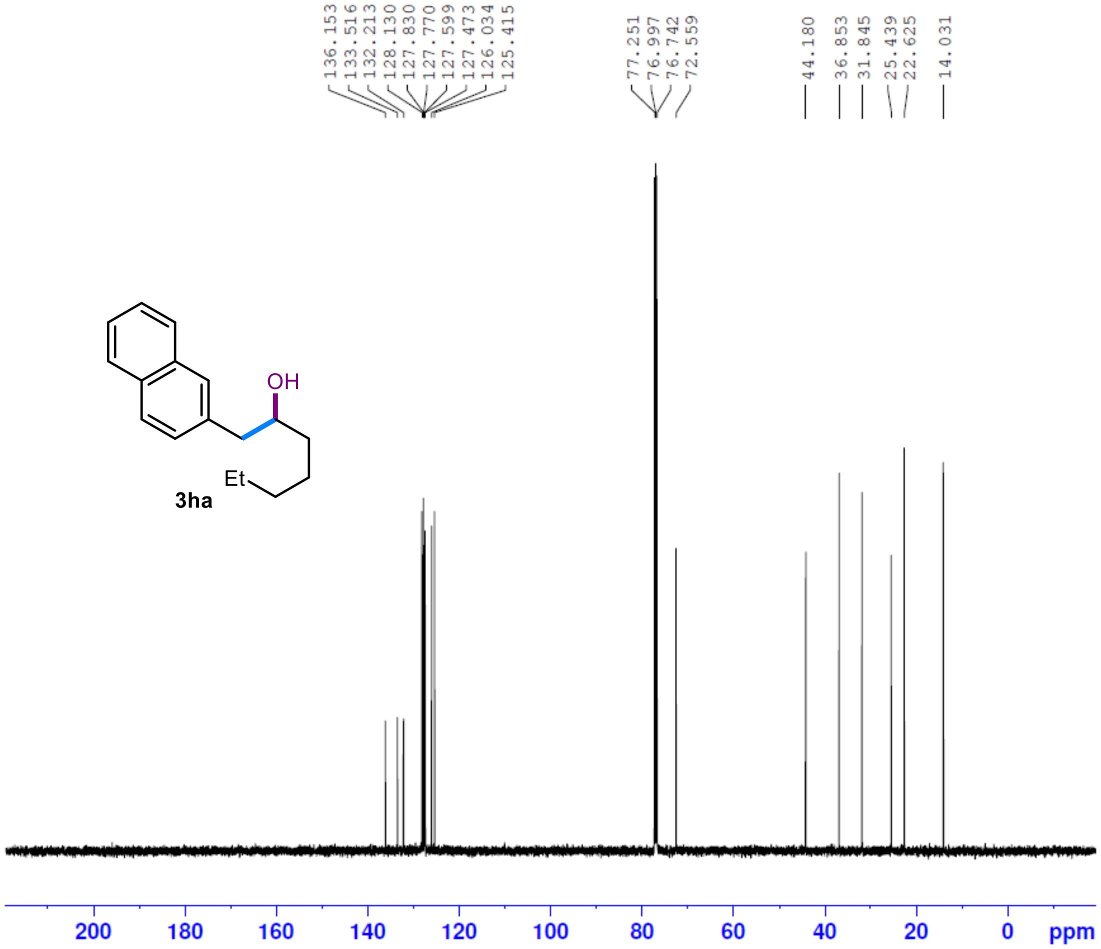

BRUKER

Current Data Parameters
NAME lcc-2124-product
EXPNO 2
PROCNO 1

F2 - Acquisition Parameters
Date_ 20200309
Time 10.57 h
INSTRUM AVIII500HD
PROBHD Z119470_0125 (
PULPROG zgpg30
TD 65536
SOLVENT CDCl3
NS 128
DS 4
SWH 30000.000 Hz
FIDRES 0.915527 Hz
AQ 1.0922667 sec
RG 192.72
DW 16.667 usec
DE 20.34 usec
TE 298.0 K
D1 2.00000000 sec
D11 0.03000000 sec
TD0 1
SFO1 125.8131140 MHz
NUC1 13C
P0 3.41 usec
P1 10.23 usec
PLW1 78.00000000 W
SFO2 500.3020012 MHz
NUC2 1H
CPDPRG[2 waltz16
PCPD2 80.00 usec
PLW2 21.50000000 W
PLW12 0.57543999 W
PLW13 0.20446000 W

F2 - Processing parameters
SI 32768
SF 125.8005434 MHz
WDW EM
SSB 0
LB 1.00 Hz
GB 0
PC 1.40

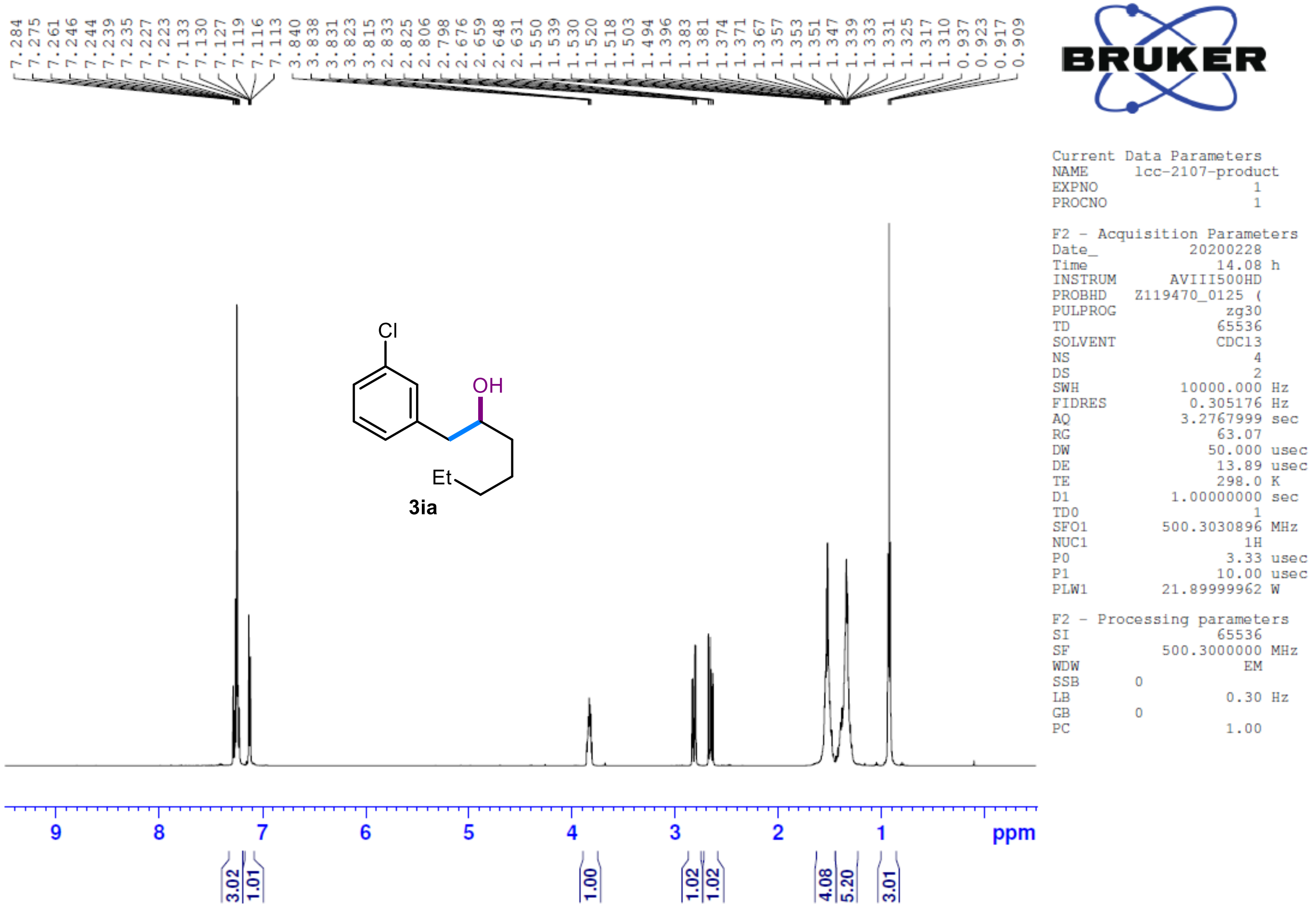
7.284 7.275 7.261 7.246 7.244 7.239 7.235 7.227 7.223 7.133 7.130 7.127 7.119 7.116 7.113 3.840 3.838 3.831 3.823 3.815 2.833 2.825 2.806 2.798 2.676 2.659 2.648 2.631 1.550 1.539 1.530 1.520 1.518 1.503 1.494 1.396 1.383 1.381 1.374 1.371 1.367 1.357 1.353 1.351 1.347 1.339 1.333 1.331 1.325 1.317 1.310 0.937 0.923 0.917 0.909
Cl
OH
Et
3ia
Current Data Parameters
NAME lcc-2107-product
EXPNO 1
PROCNO 1
F2 - Acquisition Parameters
Date_ 20200228
Time 14.08 h
INSTRUM AVIII500HD
PROBHD Z119470_0125 (
PULPROG zg30
TD 65536
SOLVENT CDCl3
NS 4
DS 2
SWH 10000.000 Hz
FIDRES 0.305176 Hz
AQ 3.2767999 sec
RG 63.07
DW 50.000 usec
DE 13.89 usec
TE 298.0 K
D1 1.00000000 sec
TD0 1
SFO1 500.3030896 MHz
NUC1 1H
P0 3.33 usec
P1 10.00 usec
PLW1 21.89999962 W
F2 - Processing parameters
SI 65536
SF 500.3000000 MHz
WDW EM
SSB 0
LB 0.30 Hz
GB 0
PC 1.00
9 8 7 6 5 4 3 2 1 ppm
3.02 1.01 1.00 1.02 1.02 4.08 5.20 3.01

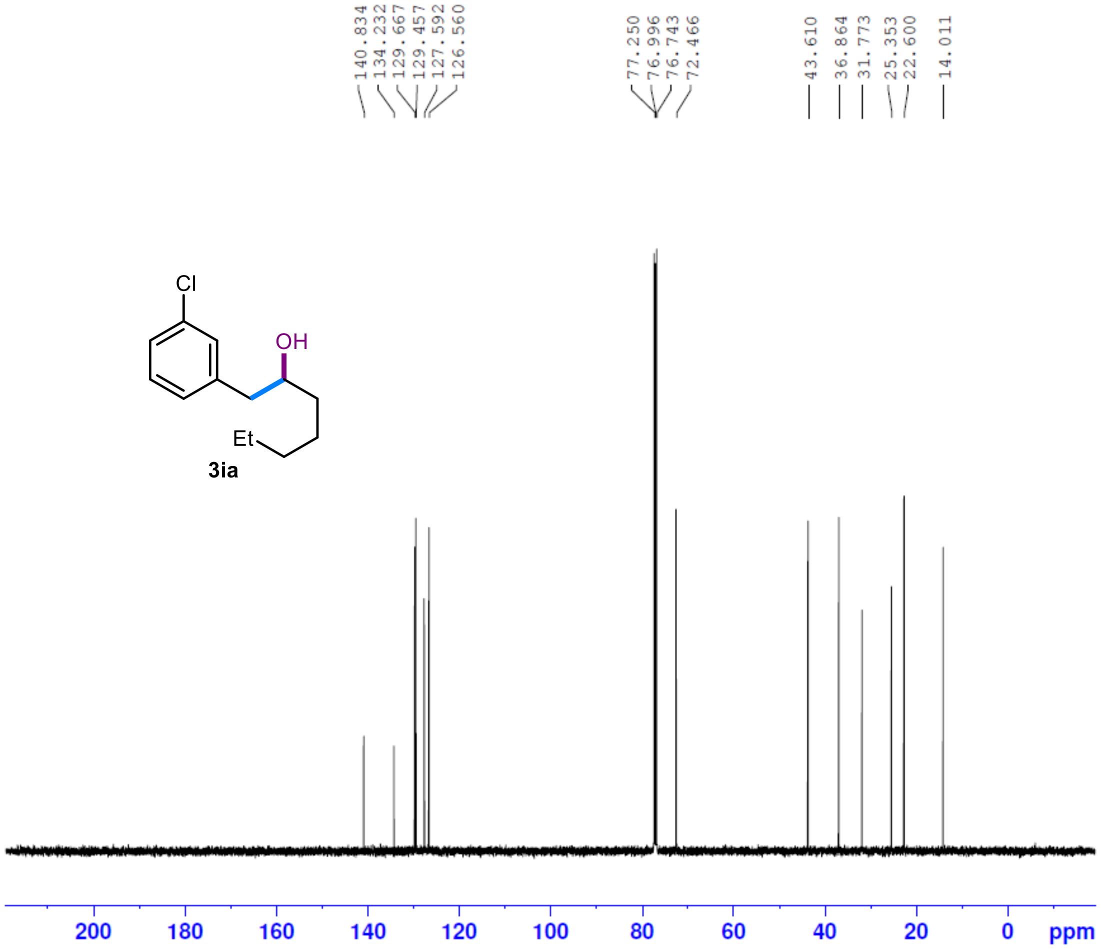

3ia

Current Data Parameters

NAME	lcc-2107-product
EXPNO	2
PROCNO	1

F2 - Acquisition Parameters

Date_	20200228
Time	14.15 h
INSTRUM	AVIII500HD
PROBHD	Z119470_0125 (
PULPROG	zgpg30
TD	65536
SOLVENT	CDCl3
NS	128
DS	4
SWH	30000.000 Hz
FIDRES	0.915527 Hz
AQ	1.0922667 sec
RG	192.72
DW	16.667 usec
DE	20.34 usec
TE	298.0 K
D1	2.00000000 sec
D11	0.03000000 sec
TD0	1
SFO1	125.8131140 MHz
NUC1	13C
P0	3.41 usec
P1	10.23 usec
PLW1	78.00000000 W
SFO2	500.3020012 MHz
NUC2	1H
CPDPRG[2	waltz16
PCPD2	80.00 usec
PLW2	21.50000000 W
PLW12	0.57543999 W
PLW13	0.20446000 W

F2 - Processing parameters

SI	32768
SF	125.8005403 MHz
WDW	EM
SSB	0
LB	1.00 Hz
GB	0
PC	1.40

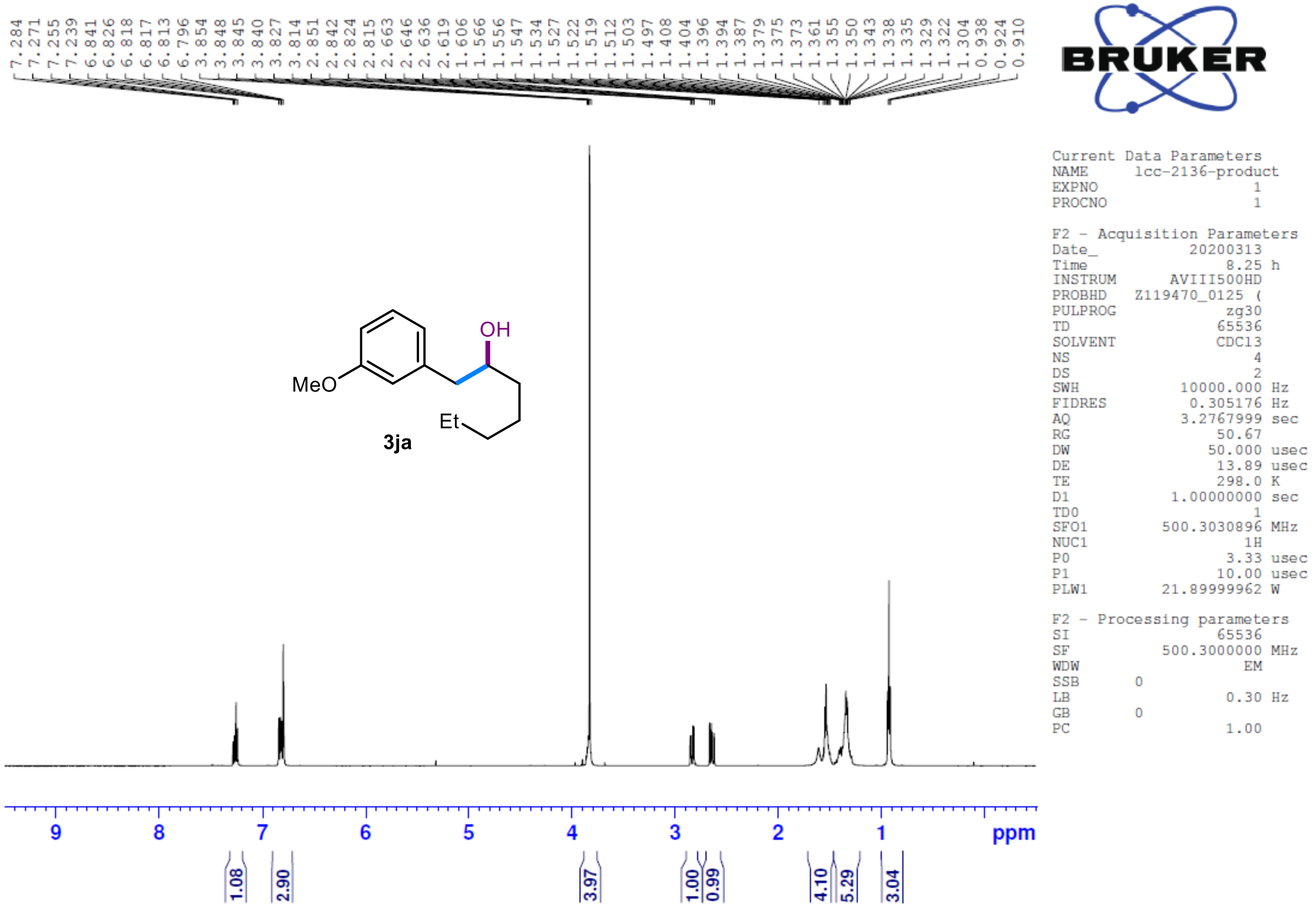
7.284 7.271 7.255 7.239 6.841 6.826 6.818 6.817 6.813 6.796 3.854 3.848 3.845 3.840 3.827 3.814 2.851 2.842 2.824 2.815 2.663 2.646 2.636 2.619 1.606 1.566 1.556 1.547 1.534 1.527 1.522 1.519 1.512 1.503 1.497 1.408 1.404 1.396 1.394 1.387 1.379 1.375 1.373 1.361 1.355 1.350 1.343 1.338 1.335 1.329 1.322 1.304 0.938 0.924 0.910
BRUKER
OH
MeO
Et
3ja
Current Data Parameters
NAME lcc-2136-product
EXPNO 1
PROCNO 1
F2 - Acquisition Parameters
Date_ 20200313
Time 8.25 h
INSTRUM AVIII500HD
PROBHD Z119470_0125 (
PULPROG zg30
TD 65536
SOLVENT CDCl3
NS 4
DS 2
SWH 10000.000 Hz
FIDRES 0.305176 Hz
AQ 3.2767999 sec
RG 50.67
DW 50.000 usec
DE 13.89 usec
TE 298.0 K
D1 1.00000000 sec
TD0 1
SFO1 500.3030896 MHz
NUC1 1H
P0 3.33 usec
P1 10.00 usec
PLW1 21.89999962 W
F2 - Processing parameters
SI 65536
SF 500.3000000 MHz
WDW EM
SSB 0
LB 0.30 Hz
GB 0
PC 1.00
9 8 7 6 5 4 3 2 1 ppm
1.08 2.90 3.97 1.00 0.99 4.10 5.29 3.04

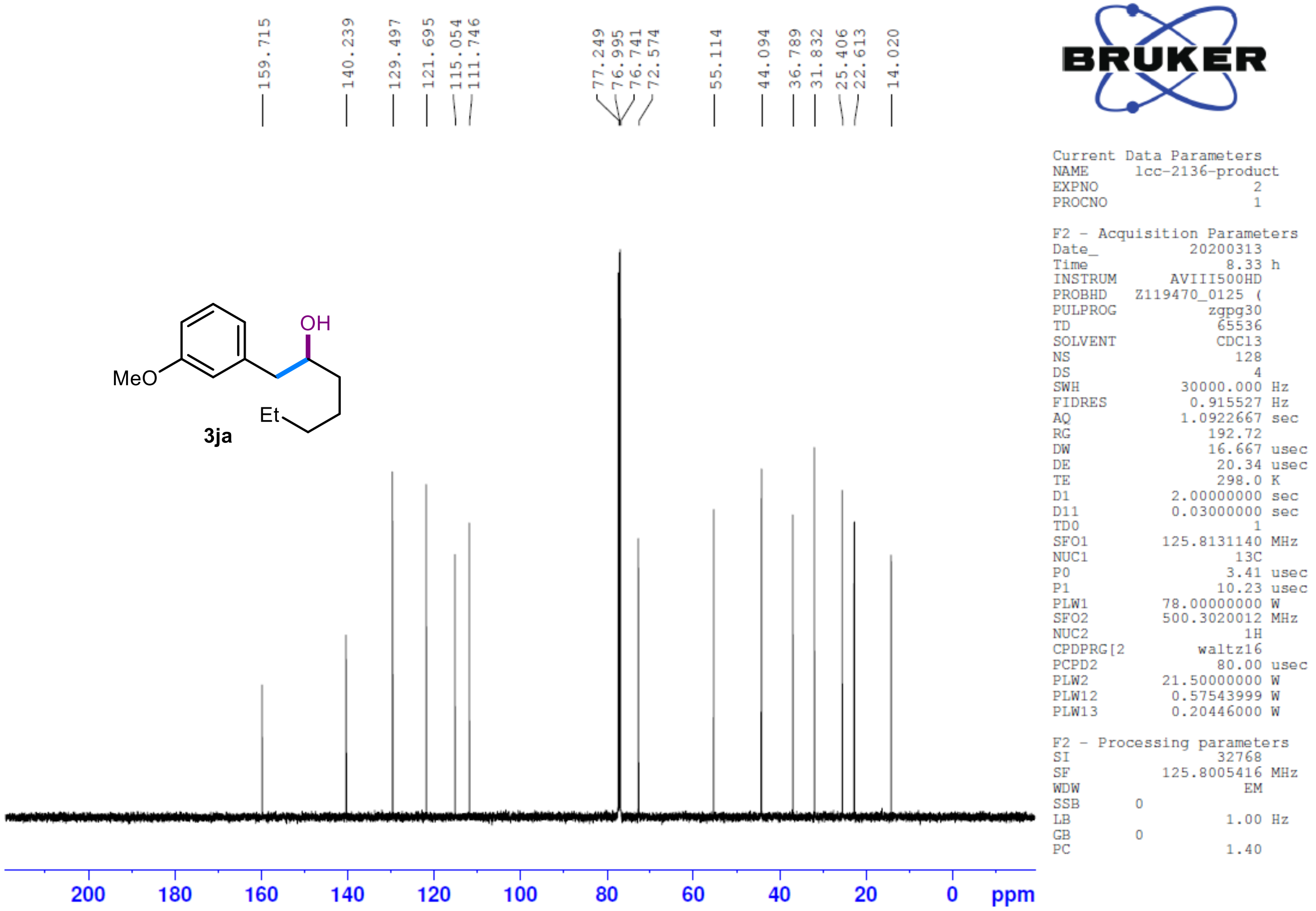
159.715
140.239
129.497
121.695
115.054
111.746
77.249
76.995
76.741
72.574
55.114
44.094
36.789
31.832
25.406
22.613
14.020
OH
MeO
Et
3ja
200 180 160 140 120 100 80 60 40 20 0 ppm
Current Data Parameters
NAME lcc-2136-product
EXPNO 2
PROCNO 1
F2 - Acquisition Parameters
Date_ 20200313
Time 8.33 h
INSTRUM AVIII500HD
PROBHD Z119470_0125 (
PULPROG zgpg30
TD 65536
SOLVENT CDCl3
NS 128
DS 4
SWH 30000.000 Hz
FIDRES 0.915527 Hz
AQ 1.0922667 sec
RG 192.72
DW 16.667 usec
DE 20.34 usec
TE 298.0 K
D1 2.00000000 sec
D11 0.03000000 sec
TD0 1
SFO1 125.8131140 MHz
NUC1 13C
P0 3.41 usec
P1 10.23 usec
PLW1 78.00000000 W
SFO2 500.3020012 MHz
NUC2 1H
CPDPRG[2 waltz16
PCPD2 80.00 usec
PLW2 21.50000000 W
PLW12 0.57543999 W
PLW13 0.20446000 W
F2 - Processing parameters
SI 32768
SF 125.8005416 MHz
WDW EM
SSB 0
LB 1.00 Hz
GB 0
PC 1.40

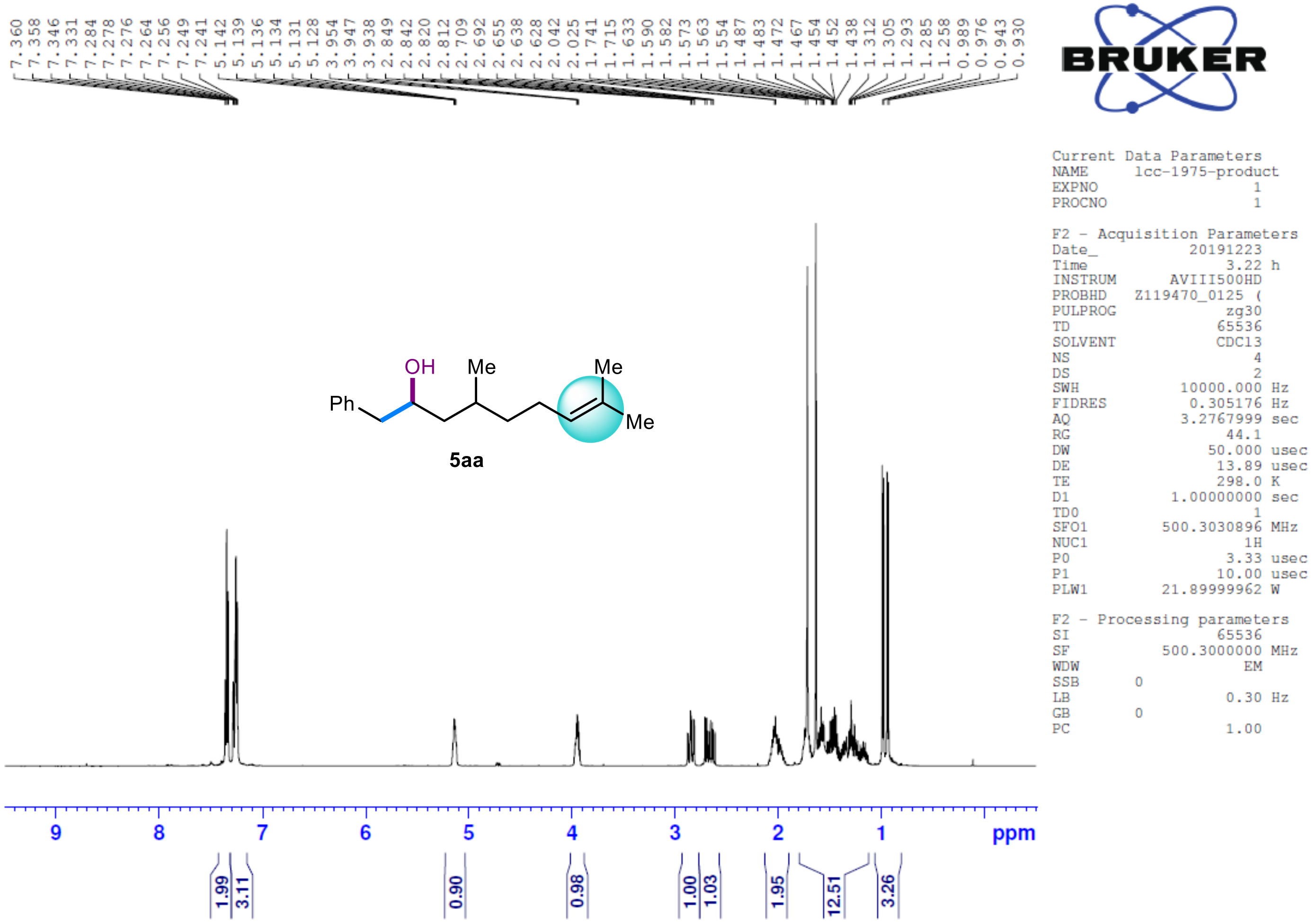
7.360
7.358
7.346
7.331
7.284
7.278
7.276
7.264
7.256
7.249
7.241
5.142
5.139
5.136
5.134
5.131
5.128
3.954
3.947
3.938
2.849
2.842
2.820
2.812
2.709
2.692
2.655
2.638
2.628
2.042
2.025
1.741
1.715
1.633
1.590
1.582
1.573
1.563
1.554
1.487
1.483
1.472
1.467
1.454
1.452
1.438
1.312
1.305
1.293
1.285
1.258
0.989
0.976
0.943
0.930
BRUKER
Current Data Parameters
NAME lcc-1975-product
EXPNO 1
PROCNO 1
F2 - Acquisition Parameters
Date_ 20191223
Time 3.22 h
INSTRUM AVIII500HD
PROBHD Z119470_0125 (
PULPROG zg30
TD 65536
SOLVENT CDCl3
NS 4
DS 2
SWH 10000.000 Hz
FIDRES 0.305176 Hz
AQ 3.2767999 sec
RG 44.1
DW 50.000 usec
DE 13.89 usec
TE 298.0 K
D1 1.00000000 sec
TD0 1
SFO1 500.3030896 MHz
NUC1 1H
P0 3.33 usec
P1 10.00 usec
PLW1 21.89999962 W
F2 - Processing parameters
SI 65536
SF 500.3000000 MHz
WDW EM
SSB 0
LB 0.30 Hz
GB 0
PC 1.00
OH
Me
Ph
Me
Me
5aa
9
8
7
6
5
4
3
2
1
ppm
1.99
3.11
0.90
0.98
1.00
1.03
1.95
12.51
3.26

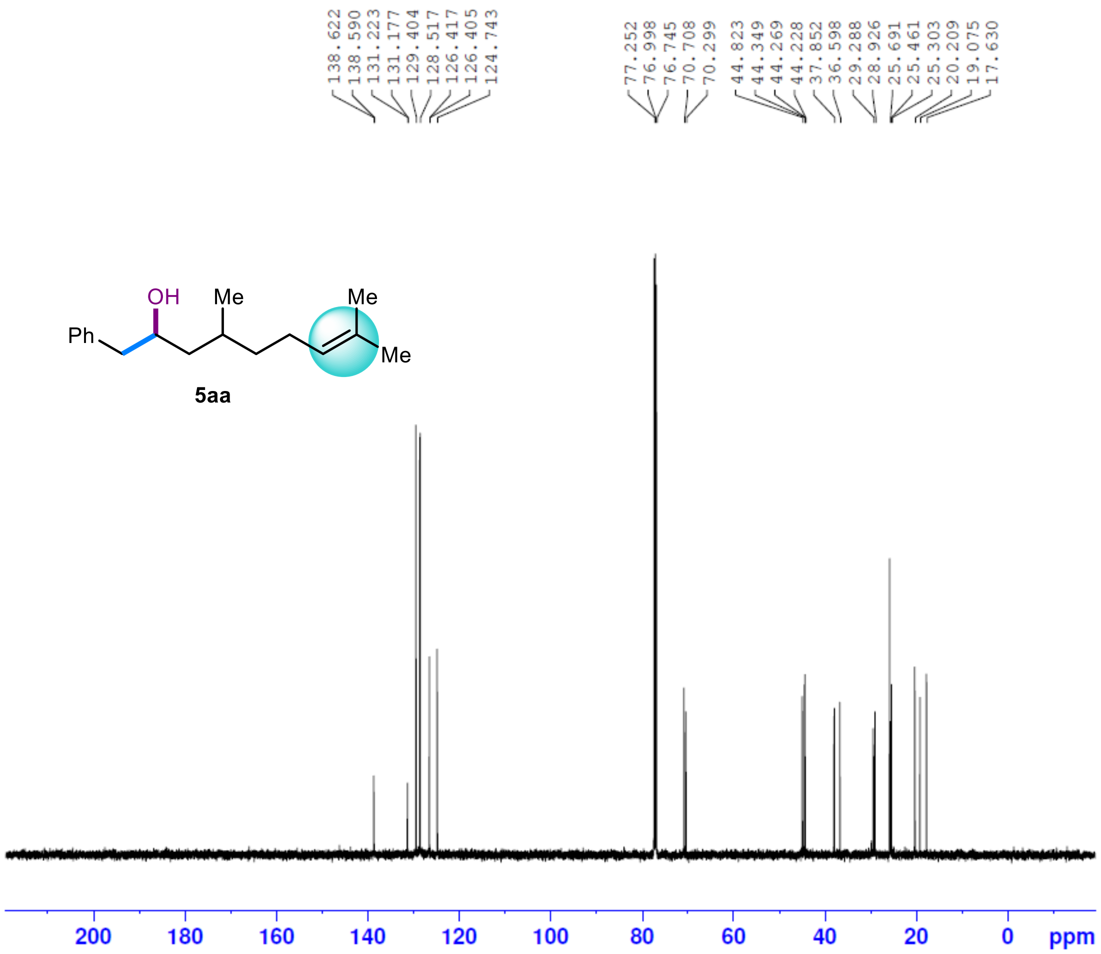


```
Current Data Parameters
NAME      lcc-1975-product
EXPNO                    2
PROCNO                   1

F2 - Acquisition Parameters
Date_             20191223
Time                  3.29 h
INSTRUM          AVIII500HD
PROBHD    Z119470_0125 (
PULPROG             zgpg30
TD                   65536
SOLVENT              CDCl3
NS                     128
DS                       4
SWH              30000.000 Hz
FIDRES            0.915527 Hz
AQ               1.0922667 sec
RG                  192.72
DW                  16.667 usec
DE                   20.34 usec
TE                   298.0 K
D1              2.00000000 sec
D11             0.03000000 sec
TD0                      1
SFO1           125.8131140 MHz
NUC1                   13C
P0                    3.41 usec
P1                   10.23 usec
PLW1           78.00000000 W
SFO2           500.3020012 MHz
NUC2                    1H
CPDPRG[2           waltz16
PCPD2                80.00 usec
PLW2           21.50000000 W
PLW12           0.49000001 W
PLW13           0.20446000 W

F2 - Processing parameters
SI                   32768
SF             125.8005416 MHz
WDW                     EM
SSB       0
LB                    1.00 Hz
GB        0
PC                    1.40
```

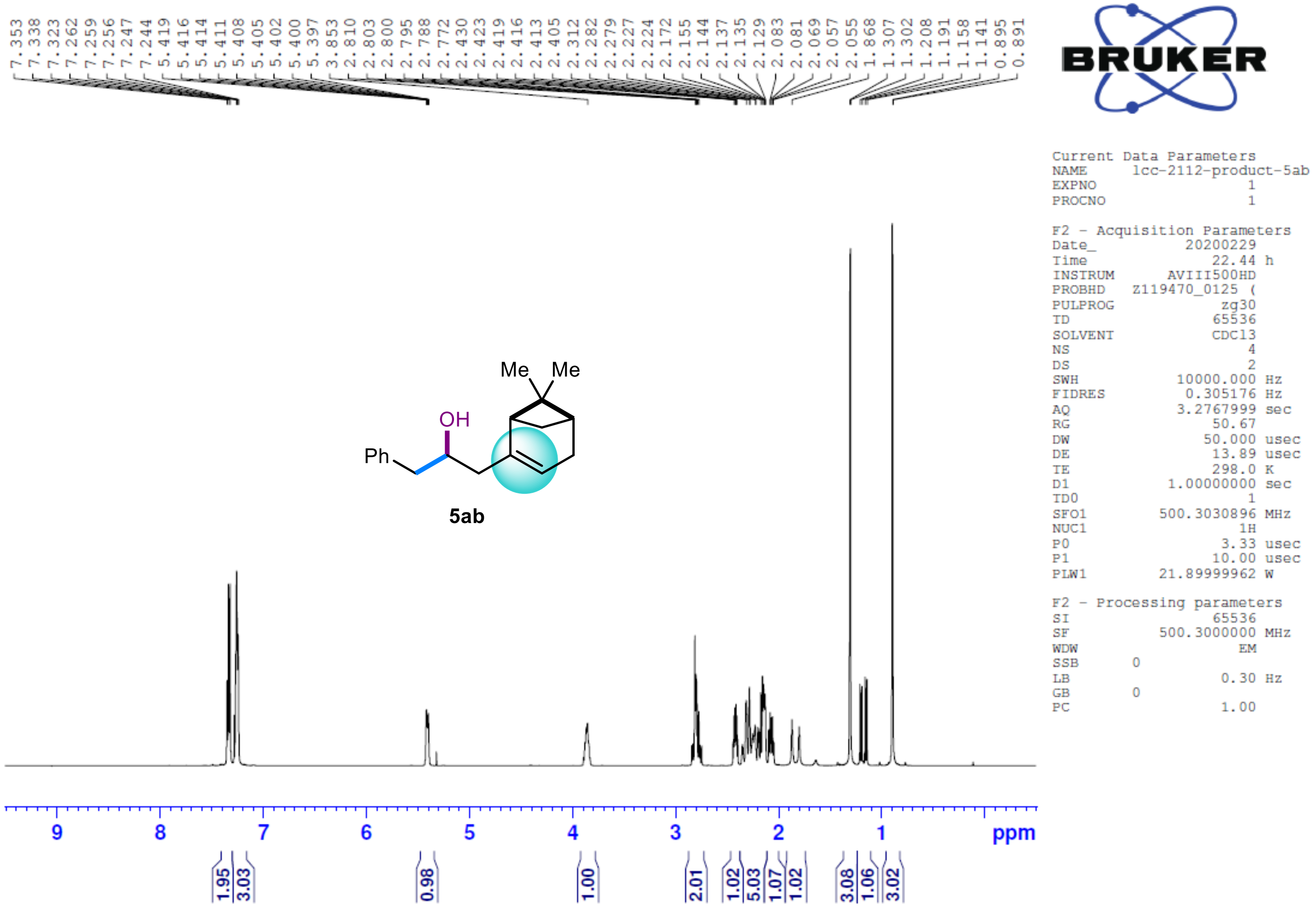
7.353 7.338 7.323 7.262 7.259 7.256 7.247 7.244 5.419 5.416 5.414 5.411 5.408 5.405 5.402 5.400 5.397 3.853 2.810 2.803 2.800 2.795 2.788 2.772 2.430 2.423 2.419 2.416 2.413 2.405 2.312 2.282 2.279 2.227 2.224 2.172 2.155 2.144 2.137 2.135 2.129 2.083 2.081 2.069 2.057 2.055 1.868 1.307 1.302 1.208 1.191 1.158 1.141 0.895 0.891
BRUKER
Me Me
OH
Ph
5ab
Current Data Parameters
NAME lcc-2112-product-5ab
EXPNO 1
PROCNO 1
F2 - Acquisition Parameters
Date_ 20200229
Time 22.44 h
INSTRUM AVIII500HD
PROBHD Z119470_0125 (
PULPROG zg30
TD 65536
SOLVENT CDCl3
NS 4
DS 2
SWH 10000.000 Hz
FIDRES 0.305176 Hz
AQ 3.2767999 sec
RG 50.67
DW 50.000 usec
DE 13.89 usec
TE 298.0 K
D1 1.00000000 sec
TD0 1
SFO1 500.3030896 MHz
NUC1 1H
P0 3.33 usec
P1 10.00 usec
PLW1 21.89999962 W
F2 - Processing parameters
SI 65536
SF 500.3000000 MHz
WDW EM
SSB 0
LB 0.30 Hz
GB 0
PC 1.00
9 8 7 6 5 4 3 2 1 ppm
1.95 3.03 0.98 1.00 2.01 1.02 5.03 1.07 1.02 3.08 1.06 3.02

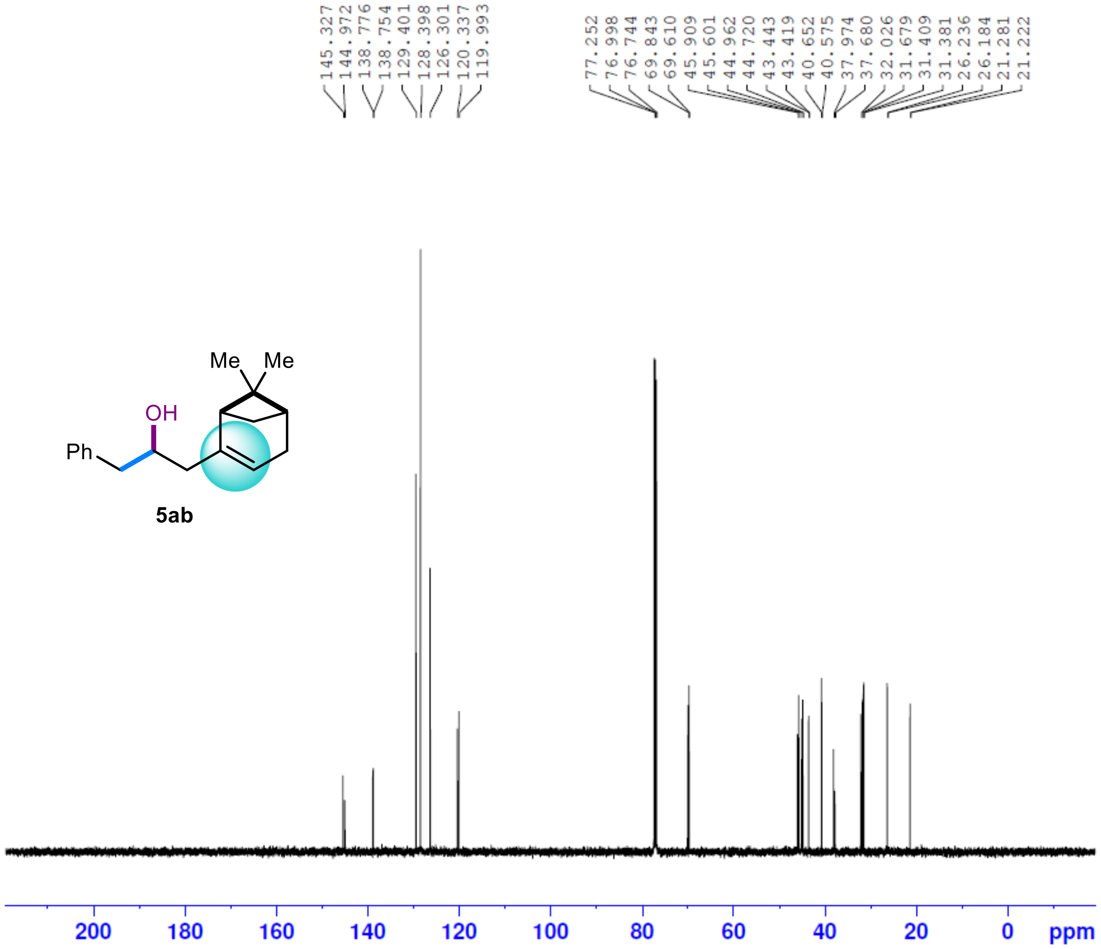

Current Data Parameters

NAME	lcc-2112-product-5ab	
EXPNO	2	
PROCNO	1	

F2 - Acquisition Parameters

Date_	20200229	
Time	22.52	h
INSTRUM	AVIII500HD	
PROBHD	Z119470_0125 (	
PULPROG	zgpg30	
TD	65536	
SOLVENT	CDCl3	
NS	128	
DS	4	
SWH	30000.000	Hz
FIDRES	0.915527	Hz
AQ	1.0922667	sec
RG	192.72	
DW	16.667	usec
DE	20.34	usec
TE	298.0	K
D1	2.00000000	sec
D11	0.03000000	sec
TD0	1	
SFO1	125.8131140	MHz
NUC1	13C	
P0	3.41	usec
P1	10.23	usec
PLW1	78.00000000	W
SFO2	500.3020012	MHz
NUC2	1H	
CPDPRG[2	waltz16	
PCPD2	80.00	usec
PLW2	21.50000000	W
PLW12	0.57543999	W
PLW13	0.20446000	W

F2 - Processing parameters

SI	32768	
SF	125.8005416	MHz
WDW	EM	
SSB	0	
LB	1.00	Hz
GB	0	
PC	1.40	

VII. Supplementary references

1 M. J. Frisch, G. W. Trucks, H. B. Schlegel, G. E. Scuseria, M. A. Robb, J. R. Cheeseman, G. Scalmani, V. Barone, G. A. Petersson, H. Nakatsuji, X. Li, M. Caricato, A. V. Marenich, J. Bloino, B. G. Janesko, R. Gomperts, B. Mennucci, H. P. Hratchian, J. V. Ortiz, A. F. Izmaylov, J. L. Sonnenberg, D. Williams-Young, F. Ding, F. Lipparini, F. Egidi, J. Goings, B. Peng, A. Petrone, T. Henderson, D. Ranasinghe, V. G. Zakrzewski, J. Gao, N. Rega, G. Zheng, W. Liang, M. Hada, M. Ehara, K. Toyota, R. Fukuda, J. Hasegawa, M. Ishida, T. Nakajima, Y. Honda, O. Kitao, H. Nakai, T. Vreven, K. Throssell, J. A. Montgomery, Jr., J. E. Peralta, F. Ogliaro, M. J. Bearpark, J. J. Heyd, E. N. Brothers, K. N. Kudin, V. N. Staroverov, T. A. Keith, R. Kobayashi, J. Normand, K. Raghavachari, A. P. Rendell, J. C. Burant, S. S. Iyengar, J. Tomasi, M. Cossi, J. M. Millam, M. Klene, C. Adamo, R. Cammi, J. W. Ochterski, R. L. Martin, K. Morokuma, O. Farkas, J. B. Foresman, and D. J. Fox, Gaussian, Inc., Wallingford CT, 2016.

2 Wang, H., Dai, X.-J. & Li, C.-J. Aldehydes as Alkyl Carbanion Equivalents for Additions to Carbonyl Compounds. *Nat. Chem.* **9**, 374-378 (2017)

3 Too, P. C., Tnay, Y. L. & Chiba, S. Copper-Catalyzed Aerobic Aliphatic C–H Oxygenation with Hydroperoxides. *Beilstein J. Org. Chem.* **9**, 1217-1225 (2013).

4 Abdur-Rashid, K.; Graham, T.; Tsang, C.-W.; Chen, X.; Guo, R.; Jia, W.; Amoroso, D.; Sui-Seng, C. Method for the Production of Hydrogen from Ammonia Borane. US Patent WO 2008/141439 (2008).

5 Yan, S.-S. *et al.* Ruthenium-Catalyzed Umpolung Carboxylation of Hydrazones with Co2. *Chem. Sci.* **9**, 4873-4878 (2018).

6 Askevold, B., Khusniyarov, M. M., Herdtweck, E., Meyer, K. & Schneider, S. A Square-Planar Ruthenium(II) Complex with a Low-Spin Configuration. *Angew. Chem. Int. Ed.* **49**, 7566-7569 (2010).

7 Sandoval, C. A., Ohkuma, T., Muñiz, K. & Noyori, R. Mechanism of Asymmetric Hydrogenation of Ketones Catalyzed by BINAP/1,2-Diamine−Ruthenium(II) Complexes. *J. Am. Chem. Soc.* **125**, 13490-13503 (2003).

8 Das, U. K., Chakraborty, S., Diskin-Posner, Y. & Milstein, D. Direct Conversion of Alcohols into Alkenes by Dehydrogenative Coupling with Hydrazine/Hydrazone Catalyzed by Manganese. *Angew. Chem.* **130**, 13632-13636 (2018).

9 Garbe, M. *et al.* Manganese(I)-Catalyzed Enantioselective Hydrogenation of Ketones Using a Defined Chiral PNP Pincer Ligand. *Angew. Chem. Int. Ed.* **56**, 11237-11241 (2017).
